# The Transcriptomic Signature of RacA Activation and Inactivation Provides New Insights into the Morphogenetic Network of *Aspergillus niger*

**DOI:** 10.1371/journal.pone.0068946

**Published:** 2013-07-24

**Authors:** Min Jin Kwon, Benjamin M. Nitsche, Mark Arentshorst, Thomas R. Jørgensen, Arthur F. J. Ram, Vera Meyer

**Affiliations:** 1 Leiden University, Institute of Biology Leiden, Department Molecular Microbiology and Biotechnology, Leiden, The Netherlands; 2 Kluyver Centre for Genomics of Industrial Fermentation, Delft, The Netherlands; 3 Institute of Biotechnology, Department Applied and Molecular Microbiology, Berlin University of Technology, Berlin, Germany; Universidade de Sao Paulo, Brazil

## Abstract

RacA is the main Rho GTPase in *Aspergillus niger* regulating polarity maintenance via controlling actin dynamics. Both deletion and dominant activation of RacA (Rac^G18V^) provoke an actin localization defect and thereby loss of polarized tip extension, resulting in frequent dichotomous branching in the Δ*racA* strain and an apolar growing phenotype for Rac^G18V^. In the current study the transcriptomics and physiological consequences of these morphological changes were investigated and compared with the data of the morphogenetic network model for the dichotomous branching mutant *ramosa-1*. This integrated approach revealed that polar tip growth is most likely orchestrated by the concerted activities of phospholipid signaling, sphingolipid signaling, TORC2 signaling, calcium signaling and CWI signaling pathways. The transcriptomic signatures and the reconstructed network model for all three morphology mutants (Δ*racA*, Rac^G18V^, *ramosa-1*) imply that these pathways become integrated to bring about different physiological adaptations including changes in sterol, zinc and amino acid metabolism and changes in ion transport and protein trafficking. Finally, the fate of exocytotic (SncA) and endocytotic (AbpA, SlaB) markers in the dichotomous branching mutant Δ*racA* was followed, demonstrating that hyperbranching does not *per se* result in increased protein secretion.

## Introduction

Filamentous fungi such as *Aspergillus niger* are widely used in biotechnology for the production of various proteins, enzymes, food ingredients and pharmaceuticals [1]–[4]. During recent years, *A. niger* became an industrial model fungus, due to its well annotated genome sequence, sophisticated transcriptomics and proteomics technologies and newly established gene transfer systems allowing efficient and targeted genetic and metabolic engineering approaches [3], [5]–[7].

The morphology of filamentous fungi strongly affects the productivity of industrial fermentations [8]–[10]. Basically, *Aspergilli* and all other filamentous fungi grow either as pellets or as freely dispersed mycelium during submerged growth. Both macromorphologies depend among other things on hyphal branching frequencies – pellets are formed when hyphae branch with a high frequency, dispersed mycelia are a result of low branching frequencies. Whereas the formation of pellets is less desirable because of the high proportion of biomass in a pellet that does not contribute to product formation, long, unbranched hyphae are sensitive to shear forces in a bioreactor. Lysis of hyphae and the subsequent release of intracellular proteases have thus a negative effect on protein production. Hence, from an applied point of view, the preferred fungal macromorphology would consist of dispersed mycelia with short filaments derived from an optimum branching frequency. It is generally accepted that protein secretion occurs mainly at the hyphal apex [11]–[14]. Some studies suggested a positive correlation between the amount of hyphal branches and protein secretion yields [13], [15]–[17], whereas other reports demonstrated no correlation [9], [18]. Therefore, it is still a matter of debate whether a hyperbranching production strain would considerably improve protein secretion rates.

Different mutations can lead to a hyperbranching phenotype in filamentous fungi. For example, dichotomous branching (tip splitting) is a characteristic of the actin (*act^1^*) and actinin mutants in *Neurospora crassa* and *A. nidulans* [19], [20], a consequence of deleting the formin SepA in *A. nidulans* [21] or the polarisome component SpaA in *A. nidulans* and *A. niger* [22], [23] and a consequence of inactivating the Rho GTPase RacA or the TORC2 complex component RmsA protein in *A. niger* [24], [25]. Common to these different gene mutations is not only the phenotype they provoke but that they also disturb the dynamics of the actin cytoskeleton. Actin is crucial for polarized hyphal growth in filamentous fungi and controls many cellular processes, including intracellular movement of organelles, protein secretion, endocytosis and cytokinesis [26], [27].

We have recently analyzed the function of all six Rho GTPase encoded in the genome of *A. niger* (RacA, CftA, RhoA, RhoB, RhoC, RhoD) and uncovered that apical dominance in young germlings and mature hyphae of *A. niger* is predominantly controlled by RacA [24]. Both RacA and CftA are not essential for *A. niger* (in contrast to RhoA) but share related functions which are executed in unicellular fungi only by Cdc42p [24]. The data showed that RacA localizes to the apex of actively growing filaments, where it is crucial for actin distribution. Both deletion and dominant activation of RacA (RacA^G18V^ expressed under control of the maltose-inducible glucoamlylase promoter *glaA*) provoke an actin localization defect and thereby loss of polarized tip extension. In the case of RacA inactivation, actin becomes hyperpolarized, leading to frequent dichotomous branching. Dominant activation of RacA, however, causes actin depolarization, leading to a swollen-tip phenotype and the formation of bulbous lateral branches. Interestingly, the dichotomous branching phenotype suggested that loss of apical dominance in Δ*racA* can frequently be overcome by the establishment of two new sites of polarized growth. This phenotype resembles the phenotype of the *ramosa-1* mutant of *A. niger*, which harbors an temperature-sensitive mutation in the TORC2 component RmsA causing a transient contraction of the actin cytoskeleton [25], [28], [29].

Altogether, the data supported the model that RacA is important to stabilize polarity axes of *A. niger* hyphae via controlling actin (de)polymerization at the hyphal apex. The aim of the present study was to unravel the genetic network into which RacA is embedded and which, when disturbed due to deletion or dominant activation of RacA, leads to loss of polarity maintenance and in the case of Δ*racA*, to reestablishment of two new polarity axes. To determine whether the hyperbranching phenotype of Δ*racA* leads to an increase in the amount of secreted proteins, the transcriptomes of our previously established RacA mutant strains (Δ*racA*, P*glaA*::*racA^G18V^*) were compared with the transcriptomes of the respective reference strains (wt, P*glaA*::*racA*). By applying defined culture conditions in bioreactor cultivations, branching morphologies as well as physiological parameters including specific growth rate and protein production rate were characterized. Finally, the implication of Δ*racA* on endocytosis and exocytosis in *A. niger* was examined by analyzing reporter strains harboring fluorescently tagged SlaB and AbpA (markers for endocytotic actin) and SncA (marker for secretory vesicles). The data obtained were compared with transcriptomic and physiological data of the dichotomous branching mutant *ramosa-1* [25], thereby providing new insights into the morphogenetic network of *A. niger*.

## Results

### Physiological consequences of RacA inactivation

**Figure 1:**
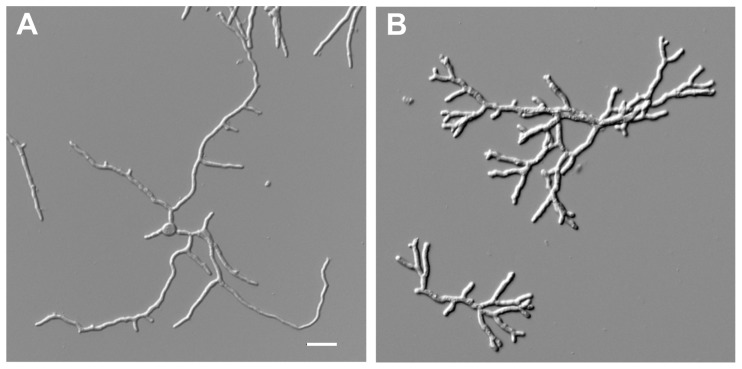
Hyphal morphology during dispersed growth. Mycelial samples of the wild-type strain N402 (A) and the Δ*racA* mutant strain (B) were taken during the mid-exponential phase when approximately 75% of the carbon source was consumed. Bar, 20 µm.

**Figure 2:**
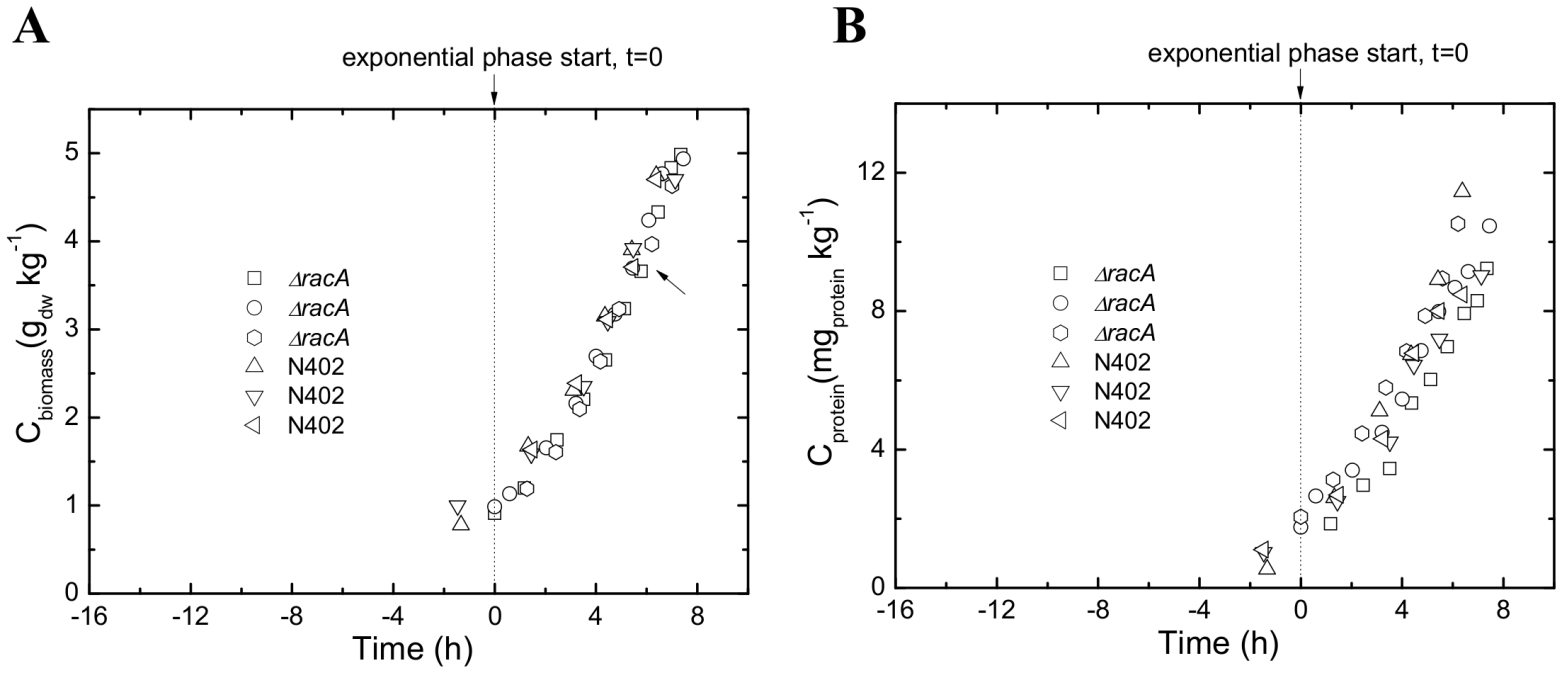
Biomass (A) and extracellular protein (B) accumulation for the wild-type strain N402 and the Δ*racA* strain. The arrow indicates the time point when biomass samples were harvested for transcriptomics analyses. The graphs represent data for three independent biological replicate cultures per strain.

As previously reported, deletion of *racA* in *A. niger* provokes hyperbranching germ tubes and hyphae, which are shorter in length but wider in hyphal diameter. This frequent branching results on solid medium in a more compact colony with a reduced diameter due to slower tip extension rates [24]. In order to further characterize the implications of loss of RacA function, the reference strain (wild-type N402) and the Δ*racA* strain were cultivated in triplicate batch cultures using maltose as growth-limiting carbon source. Propagation of both strains gave rise to homogeneous cultures of dispersed mycelia, whereby loss of RacA resulted in an about 30% higher branching frequency (Fig. 1 and Table 1). Physiological profiles including growth curves, maximum specific growth rates and specific protein secretion rates were obtained with high reproducibility and were nearly identical for both strains despite the significant difference in their morphology (Fig. 2 and Table 2). This result might come with surprise because of the negative effect of the *racA* deletion on radial colony growth on solid medium [24]. However, growth on solid media can only be assessed based on colony diameter (reflecting tip extension) and not on biomass accumulation (i.e. increase in cell volume per time). During exponential growth, growth yield on substrate (Y_x/s_) was comparable in both strains; 0.63±0.03 and 0.60±0.02 g_biomass_ g_maltose_^−1^ for Δ*racA* and N402, respectively. Notably, the amount of extracellular protein was not altered in Δ*racA* strain compared to N402 (Table 2). Hence, an increased branching frequency is the only highly significant consequence of *racA* disruption, which, however, does not *per se* result in higher protein secretion rates.

**Table 1:** Comparative image analysis of branching morphologies.

	N402 (n = 38)	*ΔracA* (n = 13)
Mycelium length (µm)	510±177	507±184
No. of hyphal apices	**17±6**	**22±8**
Branch length (µm)	**32±6**	**23±2**
Central hyphal length (µm)	**257±63**	**162±41**

Morphological samples were taken from the exponential growth phase and the individual mycelium was randomly selected to measure the length of the mycelium and the number of branching tips using imageJ. Mean values ± standard deviations are given. Bold letters indicate significant differences (two tailed t-test, p<0.01).

**Table 2:** Physiological characterization of N402 and Δ*racA* strains.

	N402	*ΔracA*
Maximum specific growth rate (h^−1^)	0.22±0.01	0.24±0.01
Yield (g_dw_ g_maltose_^−1^)	0.60±0.02	0.63±0.03
Respiratory quotient (RQ)	0.97±0.05	1.03±0.03
Acidification (mmol_base_ g_dw_^−1^ h^−1^)	1.19±0.06	1.17±0.02
Specific protein secretion rate (mg_protein_ g_dw_^−1^ h^−1^)	0.49±0.07	0.53±0.02

Biomass samples were taken from triplicate independent batch cultivations using maltose as carbon source (Fig. 2). Mean values ± standard deviations are given. No significant difference was observed with any of the variables (two tailed t-test, p<0.01). RQ, respiratory quotient calculated as the ratio of CO_2_ production and O_2_ consumption rates.

### Consequences of RacA inactivation on exo- and endocytosis

We previously showed that RacA is important for actin localization at the hyphal tip [24]. As actin is important for both exo- and endocytosis, the consequences of *racA* deletion on both was assessed in *A. niger* by following the localization of fluorescently-labelled reporter proteins SncA, AbpA and SlaB, respectively. SncA is the vesicular-SNARE that is specific for the fusion of Golgi derived secretory vesicles with the plasma membrane [30] and used as marker for exocytosis in *A. nidulans* [31], [32]. Abp1/AbpA and Sla2/SlaB are actin binding proteins and well characterized endocytic markers in yeast and filamentous fungi [31], [33], [34]. Screening of the genome sequence of *A. niger* [5] predicted for each of the established marker proteins a single orthologue for *A. niger*: An12g07570 for SncA, An03g06960 for Abp1/AbpA and An11g10320 for Sla2/SlaB.

**Figure 3:**
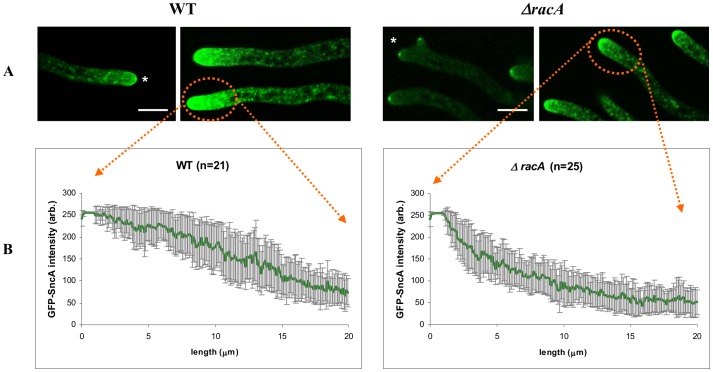
Localization of secretory vesicles and fluorescent intensity distributions in the wild-type strain N402 and the Δ*racA* mutant strain using GFP-SncA as fluorescent marker. (A) CLSM images showing the localization of GFP-SncA in hyphal tips. The Spitzenkörper is indicated with a star. (B) Fluorescent intensity distributions along hyphal tip compartments (n>20) within a region of 20 µm. Bar, 10 µm.

We constructed a reporter strain expressing a fusion of GFP with the v-SNARE SncA as described elsewhere (Kwon *et al.*, manuscript submitted). In brief, physiological expression levels of GFP-SncA was ensured by fusing GFP between the N-terminus and the promoter of *sncA* and used this cassette to replace it with the endogenous *sncA* gene (giving strain FG7). As depicted in Figure 3A, GFP-SncA is visible as punctuate intracellular structures representing secretory vesicles. These vesicles accumulate towards the hyphal tip, overlap with the Spitzenkörper and are highest at the extreme apex, which is proposed to be the site of exocytosis in filamentous fungi [31]. This localization of GFP-SncA in *A. niger* was very similar to the localization previously reported for other filamentous fungi [14], [31], [35]–[37]. Importantly, the amount of secretory vesicles per hyphal tip was affected in the Δ*racA* strain. Although the localization of GFP-SncA was similar to the wild-type strain, the intensity of the signal was considerably lower. Quantification of the GFP signal intensities in both strains revealed that the tips of wild-type hyphae display a GFP-SncA gradient of ∼20–25 µm but only about ∼10 µm in the *racA* deletion strain (Fig. 3B). Both strains, however, do not differ in their specific protein secretion rates (Table 2), implying that the total amount of secretory vesicles is the same in both strains. This discrepancy can most easily be explained by the assumption that secretory vesicles in the hyperbranching Δ*racA* strain are merely distributed to more hyphal tips, which in consequence lowers the amount of vesicles per individual hyphal tip.

**Figure 4:**
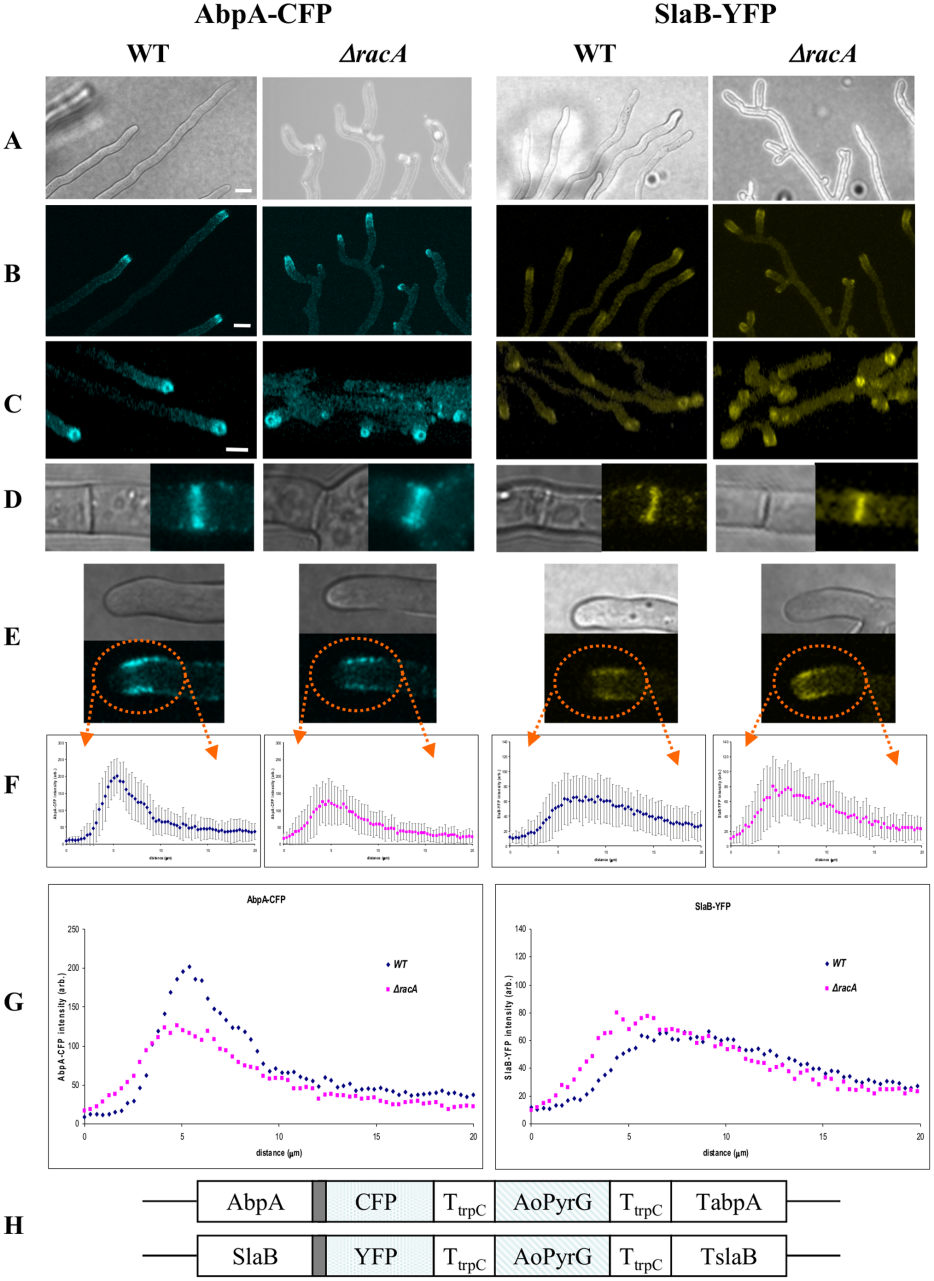
Localization of endocytic ring structures in the wild-type strain N402 and the Δ*racA* mutant strain using AbpA-CFP and SlaB-YFP as fluorescent markers. The cultures were grown for two days at 22°C on MM agar. (A) Transmission light images, (B) two dimensional fluorescent confocal images and (C) three dimensional reconstructions from z-sectional confocal images are shown for both strains. (D, E) depict selected light and fluorescent images at sites of septation (D) and at sites of endocytosis (E). (F, G) Fluorescent intensity distribution of endocytotic ring structures. Fluorescence was measured in at least 25 hyphal tips of each strain. Mean values with (F) or without (G) standard deviation is shown. (H) Schematic representation of the AbpA-CFP and SlaB-YFP constructs designed for integration at the endogenous *abpA* and *slaB* loci, respectively. The *A. oryzae pyrG* gene served as selection marker, a sequence encoding 5× Gly-Ala as peptide linker (grey box) and the 3′ region from the *A. nidulans trpC* gene as terminator. Bar, 10 µm.

To follow the effect of *racA* deletion on endocytosis, AbpA and SlaB were labeled using a C-terminal labeling strategy as previously reported for *A. nidulans* [31]. Importantly, both AbpA-CFP and SlaB-YFP were expressed at physiological levels by using the respective endogenous promoter and by replacing the constructs with the endogenous *abpA* and *slaB* gene, respectively (Fig. 4B). AbpA-CFP (strain MK6.1) and SlaB-YFP (strain MK5.1) transformants were phenotypically indistinguishable from the recipient strain, indicating that both AbpA-CFP and SlaB-YFP are functionally expressed (Fig. 4 and data not shown). Although AbpA-CFP and SlaB-YFP fluorescence signals were only weakly detectable (which is a direct consequence of their low endogenous expression level), both proteins were visible in the wild-type background as peripheral punctate structures and formed a subapical ring likely reflecting the endocytic machinery (Fig. 4A–C). Fluorescent signals were excluded from the hyphal apex which is in agreement with previous reports for *A. nidulans* showing that endocytosis occurs behind the tip [31], [34]. The signal of SlaB-YFP but not AbpA-CFP seemed to be intimately associated with the plasma membrane (data not shown), which would be in agreement with the function of both proteins - Sla2/SlaB is involved in early endocytic site initiation while Abp1/AbpA is important for invagination, scission and release of endocytotic vesicles [38]. SlaB-YFP and AbpA-CFP fluorescence was also occasionally observed at septa or sites destined for septum formation (Fig. 4D), probably suggesting an involvement of endocytotic events at septa as recently reported for *A. oryzae* [14]. Importantly, the intensity and distribution of SlaB-YFP and AbpA-CFP differed slightly in the wild-type and the Δ*racA* strain (Fig. 4E–G). The endocytotic actin ring as visualized by AbpA-CFP was sharper formed in the wild-type background but more diffuse in the Δ*racA* strain, an observation which, however, was not so evident for SlaB-YFP (Fig. 4 F–G). Still, the endocytotic ring formed by both SlaB-YFP and AbpA-CFP seemed to be positioned closer to the hyphal apex in the Δ*racA* strain (Fig. 4G), suggesting that deletion of RacA affected endocytotic processes and provoked a slight mislocalization of the endocytotic ring.

### The transcriptomic fingerprint of hyperbranching in Δ*racA*

To study the transcriptomic consequences due to deletion of *racA*, RNA samples from triplicate bioreactor cultivations were taken after both wt and Δ*racA* cultures reached the mid-exponential growth phase (biomass concentration 3.7 gr kg^−1^). A total of 139 genes out of 14,165 *A. niger* genes were identified as differentially expressed, 44 of which displayed increased and 95 genes decreased expression levels in Δ*racA* (FDR<0.05). The complete list of differentially expressed genes, including fold change and statistical significance is given in Tables S1 and S2. Interestingly, the modulated gene set in Δ*racA* is very small (139 genes, i.e. 1% of all *A. niger* genes) but in the same range as the differentially expressed gene set in the dichotomous branching mutant *ramosa-1* of *A. niger* (136 genes; [25]. Although the affected gene sets had opposite signs (44 up/95 down in Δ*racA*, 109 up/27 down in *ramosa-1*), similar processes were affected in both hyperbranching strains (Table 3): (i) (phospho)lipid signaling, (ii) calcium signaling, (iii) cell wall integrity (CWI) signaling and cell wall remodeling, (iv) γ-aminobutyric acid (GABA) metabolism and (v) transport phenomena. Specific responses for Δ*racA* but not *ramosa-1* included genes related to actin localization and protein trafficking.

**Table 3:** Selected genes whose expression profile respond to hyperbranching in Δ*racA* (this work) and *ramosa-1* [25]. Genes are ordered into different processes and functions.

Predicted protein function*	ΔracA *versus* wt			ramosa-1 *versus* wt		
	Open reading frame code	Up/Down	Closest *S. cerevisiae* ortholog	Open reading frame code	Up/Down	Closest *S. cerevisiae* ortholog
***(Phospho)lipid metabolism and signaling***						
phosphatidyl synthase, synthesis of phosphatidyl alcohols				An02g08050	**↑**	
sterol 24-C-methyltransferase, ergosterol synthesis	An04g04210	**↑**	Erg6			
C-14 sterol reductase, ergosterol synthesis	**An01g07000**	**↓**	**Erg24**	**An01g07000**	**↑**	**Erg24**
inositol hexaki-/heptaki-phosphate kinase, synthesis of IP6, IP7	An14g04590	**↓**	Kcs1	An16g05020	**↑**	Vip1
plasma membrane protein promoting PI4P synthesis				An18g06410	**↑**	Sfk1
phospholipase B, synthesis of glycerophosphocholine	An18g01090	**↓**	Plb3			
phospholipase B, synthesis of glycerophosphocholine	An02g13220	**↓**	Plb1			
phospholipase D, synthesis of phosphatidic acid				An15g07040	**↑**	Spo14
diacylglycerol pyrophosphate phosphatase, synthesis of DAG	**An04g03870**	**↓**	**Dpp1**	**An04g03870**	**↑**	**Dpp1**
diacylglycerol pyrophosphate phosphatase, synthesis of DAG	An02g01180	↓	Dpp1	An11g05330	↑	
choline/ethanolamine permease	An16g01200	**↑**	Hnm1			
choline/ethanolamine permease	An01g13290	**↓**	Hnm1			
transcription factor important for sterol uptake	An02g09780	**↓**	Upc2			
transcription factor important for sterol uptake	An12g00680	**↓**	Upc2			
mannosyl-inositol phosphorylceramide (MIPC) synthase	An05g02310	**↓**	Sur1			
***Calcium homeostasis and signaling***						
Ca^2+^/calmodulin dependent protein kinase				An02g05490	**↑**	Cmk2
Ca^2+^/calmodulin dependent protein kinase				An16g03050	**↑**	Cmk2
vacuolar Ca^2+^/H^+^ exchanger	**An01g03100**	**↓**	**Vcx1**	**An01g03100**	**↑**	**Vcx1**
vacuolar Ca^2+^/H^+^ exchanger	**An05g00170**	**↓**	**Vcx1**	**An05g00170**	**↑**	**Vcx1**
vacuolar Ca^2+^/H^+^ exchanger	An14g02010	**↓**	Vcx1			
Ca^2+^ transporting ATPase	An19g00350	**↓**	Pmc1	An02g06350	**↑**	Pmc1
Ca^2+^/phospholipid-transporting ATPase				An04g06840	**↑**	Drs2
***Cell wall remodeling and integrity***						
membrane receptor, CWI signaling	An01g14820	**↑**	Wsc2			
MAP kinase kinase, CWI signaling, MkkA				An18g03740	**↑**	Mkk1/2
plasma protein responding to CWI signaling	An07g08960	↓	Pun1			
plasma protein responding to CWI signaling	An08g01170	↓	Pun1			
α-1,3-glucanase	An04g03680	**↑**		An08g09610	**↑**	
β-1,3-glucanosyltransferase (GPI-anchored)				An16g06120	**↑**	Gas1
β-1,4-glucanase	**An03g05530**	**↓**		**An03g05530**	**↑**	
chitin synthase class II, similar to ChsA of *A. nidulans*				An07g05570	**↑**	
chitin transglycosidase (GPI-anchored)	An07g01160	↓	Crh2	An13g02510	**↑**	Crh1
chitinase (GPI-anchored), similar to ChiA of *A. nidulans*				An09g06400	**↓**	Cts1
β-mannosidase				An11g06540	**↑**	
endo-mannanase (GPI-anchored), DfgE	**An16g08090**	**↓**	**Dfg1**	**An16g08090**	**↑**	**Dfg1**
α-1,2-mannosyltransferase				An14g03910	**↑**	Kre2
α-1,2-mannosyltransferase				An18g05910	**↑**	Kre2
α-1,3-mannosyltransferase	An15g04810	↓	Mnt2			
α-1,6-mannosyltransferase	An05g02320	↓				
cell wall protein	An14g01840	↓	Tir3	An04g05550	**↑**	Flo11
cell wall protein	**An11g01190**	↓	**Sps22**	**An11g01190**	**↑**	**Sps22**
cell wall protein				An03g05560	**↑**	
cell wall protein				An04g03830	**↑**	
cell wall protein				An02g11620	**↓**	
plasma membrane protein	An02g08030	↓	Pmp3			
***GABA metabolism***						
glutaminase				An11g07960	**↑**	
γ-aminobutyrate transaminase				An17g00910	**↑**	Uga1
NAD(+)-dependent glutamate dehydrogenase				An02g14590	**↑**	Gdh2
GABA permease	An16g01920	**↑**				
transcription factor for GABA genes	An02g07950	↓				
***Transporter***						
mitochondrial phosphate translocator				An02g04160	**↑**	Mir1
mitochondrial ABC transporter during oxidative stress	An12g03150	**↓**	Mdl1			
vacuolar glutathione S-conjugate ABC-transporter				An13g02320	**↑**	Ycf1
plasma membrane Na^+^/K^+^-exchanging ATPase alpha-1 chain	**An09g00930**	**↓**		**An09g00930**	**↑**	
plasma membrane K^+^ transporter	An03g02700	**↓**	Trk1			
multidrug transporter	An01g05830	**↓**	Qdr1	An02g01480	**↑**	
low-affinity Fe(II) transporter of the plasma membrane	**An16g06300**	**↓**	**Fet4**	**An16g06300**	**↑**	**Fet4**
vacuolar H^+^-ATPase subunit, required for copper and iron homeostasis	An10g00680	**↓**	Vma3			
siderophore-iron transporter				An12g05510	**↓**	Taf1
mitochondrial carrier protein				An14g01860	**↓**	Rim2
vacuolar zinc transporter				An15g03900	**↓**	Zrc1
allantoin permease				An18g01220	**↓**	Dal5
oligopeptide transporter	An13g01760	**↑**	Opt1	An15g07460	**↓**	Opt1
oligopeptide transporter	An11g01040	**↓**	Opt1			
hexose/glucose transporter	An06g00560	**↑**	Hxt13	An09g04810	**↓**	
amino acid transporter	An03g00430	**↑**				
galactose transporter	An01g10970	**↓**	Gal2			
FAD transporter into endoplasmatic reticulum, CWI-related	An01g09050	**↓**	Flc2			
***Protein trafficking***						
GTPase activating protein involved in protein trafficking	An01g02860	**↓**	Gyp8	An15g01560	**↑**	Gyp7
t-SNARE protein important for fusion of secretory vesicles with the plasma membrane	An02g05390	**↓**	Sec9			
vacuolar protein important for endosomal-vacuolar trafficking pathway	An11g01810	**↓**	Rcr2			
***Actin localisation***						
polarisome component SpaA	An07g08290	↓	Spa2			
actin-binding protein involved in endocytosis	An03g01160	↓	Lsb4			
protein required for normal localization of actin patches	An16g02680	↓	Apd1			
Amphysin-like protein required for actin polarization	An17g01945	↓	Rvs161			
Amphysin-like protein required for actin polarization	An09g04300	↓	Rvs167			
***Other signaling processes***						
Ser/Thr protein kinase important for K^+^ uptake	An17g01925	**↓**	Sat4			
transcription factor for RNA polymerase II	An16g07220	**↓**	Tfg2			
negative regulator of Cdc42				An12g04710	**↓**	Vtc1
putative C_2_H_2_ zinc-finger transcription factor				An04g01500	**↑**	
SUN family protein involved in replication				An08g07090	**↓**	Sim1
similar to *A. nidulans* transcription factor RosA				An16g07890	**↓**	Ume6
**Others**						
hypothetical aspergillosis allergen rAsp	**An03g00770**	↓		**An03g00770**	↑	

Genes up-regulated are indicated with ↑, genes down-regulated with ↓. Differential gene expression was evaluated by moderated t-statistics using the Limma package [82] with a FDR threshold at 0.05 [83]. Identical ORFs which are differentially expressed in both Δ*racA* and *ramosa-1* are indicated in bold. Fold changes and statistical significance is given in Additional file 1 and 2.

* : Protein functions were predicted based on information inferred from the *Saccharomyces* genome data base SGD (http://www.yeastgenome.org/) and the *Aspergillus* genome database AspGD (http://www.aspergillusgenome.org/).

In the case of (phosho)lipid signaling, genes encoding enzymes for the synthesis of the key regulatory lipid molecules diacylglycerol (DAG) and inositolpyrophosphates (IP6 and IP7) were differentially expressed in both Δ*racA* and *ramosa-1*. These molecules play important roles in the regulation of actin polarisation, CWI and calcium signaling in lower and higher eukaryotes (see discussion). Notably, expression of An04g03870 predicted as ortholog of the *S. cerevisiae* Dpp1p (DAG pyrophosphate phosphatase) is affected in both strains; however, down-regulated in Δ*racA* but up-regulated in *ramosa-1*. The same opposite response was observed for two *A. niger* ORFs predicted to encode inositol hexaki-/heptaki-phosphate kinases synthesizing the signaling molecules inositol pyrophosphates IP6 or IP7: An14g04590 (Ksc1p ortholog) is down-regulated in Δ*racA*, whereas An16g05020 (Vip1p ortholog) is up-regulated in *ramosa-1*. Inositol polyphosphates IP4-IP6 are known to bind to the C2B domain of the calcium sensor protein synaptotagmin [39]. This binding inhibits exocytosis of secretory vesicles, whereas binding of calcium to the C2A domain of synaptotagmin activates exocytosis [40]. In the Δ*racA* strain, four calcium transporters are down-regulated compared to the wild-type situation, two of which (An01g03100, An05g00170) code for the ortholog of the *S. cerevisiae* Vcx1p protein, which is also differentially expressed under hyperbranching conditions in *ramosa-1* (Table 3). This observation hints at the possibility that reduced GFP-SncA fluorescence at Δ*racA* hyphal tips is somehow linked with changes in IP6/IP7 and calcium levels in Δ*racA*, which would be in agreement with a recent report which demonstrated that calcium spikes accompany hyphal branching in *Fusarium* and *Magnaporthe* hyphae [41]. Changes in the intracellular calcium distribution also affect the homeostasis of other ions. Congruently, 12 genes putatively encoding transport proteins for ions (Na^+^, K^+^, Fe^2+^) and small molecules (phospholipids, amino acids, peptides, hexoses) displayed differential transcription in Δ*racA* as also observed for *ramosa-1* (Table 3), suggesting that ion homeostatic and/or metabolic control systems are also important to maintain polar growth. Reduced exocytotic GFP-SncA signals at hyphal tips of the Δ*racA* strain would imply that less cell wall biosynthetic/remodeling enzymes are transported to the tip. Indeed, expression of ten ORFs encoding cell membrane and cell wall genes and three ORFs involved in protein trafficking were down-regulated in Δ*racA* (Table 3). Whatever the consequences of reduced expression of cell wall or ion homeostasis genes are, none of these changes led to increased sensitivity of the Δ*racA* strain towards cell wall stress agents (calcofluor white), different salts (MgCl_2_, KCl, NaCl) or oxidative conditions (H_2_O_2_, menadion; data not shown), suggesting that the integrity of the cell wall or cell membrane is not disturbed in the *racA* strain.

Inactivation of RacA, however, has considerable consequences on actin localization as previously reported [23]. Congruently, five ORFs involved in actin polarization were down-regulated in the Δ*racA* strain (Table 3). Of special importance are the polarisomal component SpaA, whose deletion has been shown to cause a hyperbranching phenotype and reduced growth speed in *A. niger* [23] and two ORFs which are homologous to the *S. cerevisiae* amphyphysin-like proteins Rvs161p and Rvs167p. The latter function as heterodimer in *S. cerevisiae*, bind to phospholipid membranes and have established roles in organization of the actin cytoskeleton, endocytosis and cell wall integrity [42].

### The transcriptomic fingerprint of apolar growth in RacA^G18V^

**Figure 5:**
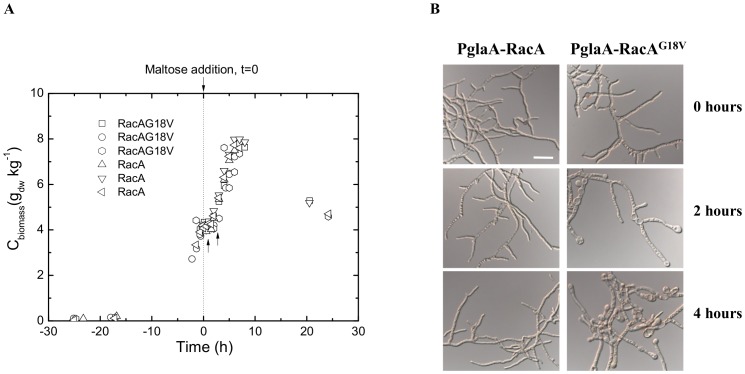
Biomass accumulation and morphology during submerged cultivation of P*glaA*-RacA^G18V^ and P*glaA*-RacA mutant strains. (A) Growth curve for both mutant strains. The dashed line indicates the time point when the inducing carbon source maltose was added. The two arrows indicate the time points at which samples for transcriptome analysis were taken. (B) Dispersed hyphal morphology at the time point of maltose addition as well as 2 and 4 h after maltose addition. Bar, 20 µm.

Next, we wished to dissect the transcriptomic adaptation of *A. niger* to dominant activation of RacA. Batch cultures of our previously established RacA mutant strains P*glaA*::*racA^G18V^* and P*glaA*::*racA* (reference strain) were started with xylose (0.75%) as repressing carbon source. After the cultures reached the exponential growth phase and xylose was consumed, maltose (0.75%) was added to induce expression of genes under control of P*glaA*. Hypothetically, a so far unknown RacA-dependent GAP ensures that the activity of RacA is spatially restricted to the hyphal apex in the P*glaA*-*racA* strain thereby maintaining a stable polarity axes even under inducing conditions (Fig. 5). However, this control mechanism is leveraged off in the P*glaA*-*racA^G18V^* mutant strain, as the GTPase-negative G18V mutation traps RacA in its active, GTP-bound form [24]. Hence, the switch from xylose to maltose leads to a loss of polarity maintenance in the P*glaA*-*racA^G18V^* strain and the formation of clavate-shaped hyphal tips and bulbous lateral branches (Fig. 5). RNA samples were extracted from duplicate cultures 2 and 4 h after the maltose shift and used for transcriptomic comparison. Expression of 3,757 (506) genes was modulated after 2 h (4 h) of induction, 1,906 (282) of which showed increased and 1,851 (224) decreased expression levels in the P*glaA*-*racA^G18V^* strain when compared to the P*glaA*-*racA* reference strain (FDR<0.05). The complete list of differentially expressed genes, including fold change and statistical significance is given in Tables S1 and S2. GO enrichment analysis using the FetGOat tool [43] discovered that most of these genes belong to primary metabolism, suggesting that both strains differed in their ability to quickly adapt to the new carbon source (Table S3).

**Figure 6:**
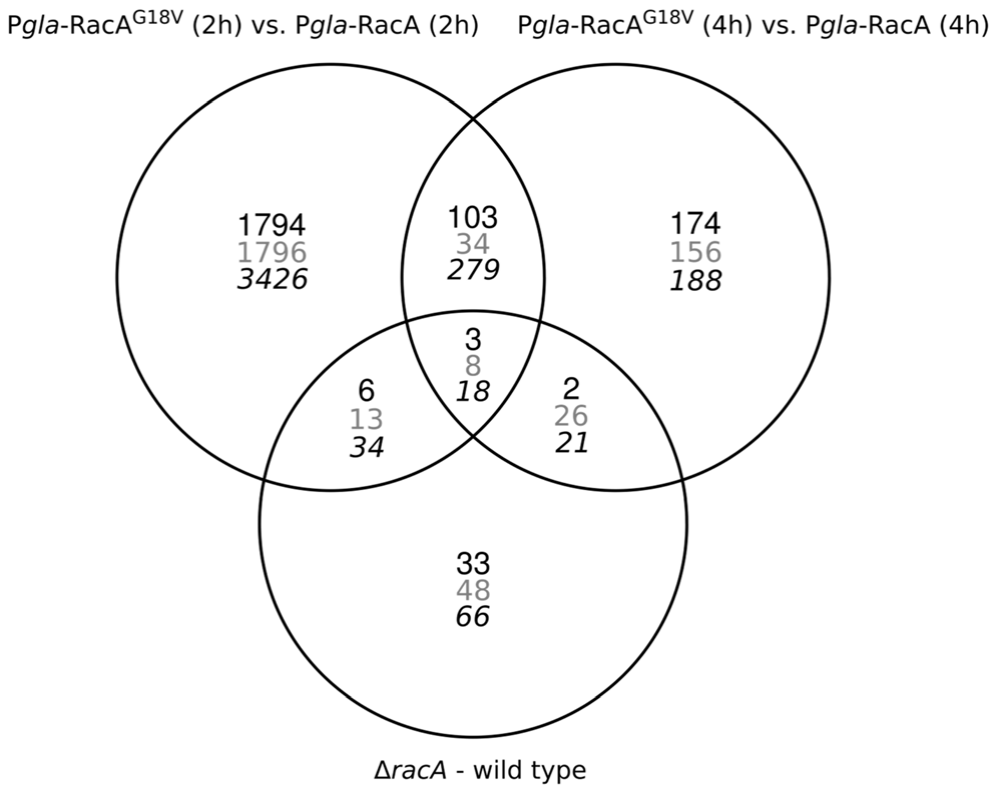
Venn diagrams of induced (black numbers), repressed (grey numbers) and up- or down-regulated (italics numbers) genes for the P*glaA*-RacA^G18V^/P*glaA*-RacA and Δ*racA*/N402 comparisons.

To identify those genes which only relate to the difference between polar to apolar morphology in P*glaA*-*racA^G18V^* but are independent from time and carbon source, a Venn diagram was constructed (Fig. 6) and the intersection determined representing genes which are differently expressed after both 2 and 4 h in P*glaA*-*racA^G18V^* when compared to the 2 h and 4 h data sets of the P*glaA*-*racA* reference strain. Overall, 148 genes showed different expression, 106 of which were up-regulated and 42 of which were down-regulated in P*glaA*-*racA^G18V^* (Table S4). Again, only a small set of genes (about 1% of the *A. niger* genome) show different expression levels during polar and apolar growth. Table 4 highlights the most interesting genes of this compilation, which could be grouped into several regulatory processes including (i) (phospho)lipid signaling, (ii) calcium signaling, (iii) CWI signaling and (iv) nitrogen signaling. In addition, metabolic processes including primary metabolism (amino acid biosynthesis) and secondary metabolism (polyketide synthesis, non-ribosomal peptide synthesis) were affected as well.

**Table 4:** Selected genes whose expression profiles differ between polar and apolar growth in P*glaA*-*racA^G18V^* versus P*glaA*-*racA* in a time- and carbon source-independent manner. Genes are ordered into different processes and functions.

Predicted protein function*	Open reading frame code	Up/Down	Closest *S. cerevisiae* ortholog
***(Phospho)lipid metabolism and signaling***			
dehydrogenase involved in sphingosin 1-phosphate breakdown	An01g09260	**↑**	Hfd1
Lysophospholipase, synthesis of glycerophosphocholine	An16g01880	**↑**	
plasma membrane flippase transporting sphingoid long chain bases	An02g06440	**↑**	Rsb1
glycerophosphocholine phosphodiesterase, synthesis of phosphocholine	An18g03170	**↑**	Gde1
lanosterol 14A-demethylase	An11g02230	**↓**	Erg11
C-14 sterol reductase, ergosterol synthesis	**An01g07000**	**↓**	**Erg24**
choline/ethanolamine permease	**An01g13290**	**↓**	**Hnm1**
***Calcium homeostasis and signaling***			
Ca^2+^/calmodulin dependent protein kinase	An02g05490	**↓**	Cmk2
***Cell wall remodeling and integrity***			
endo-glucanase EglA	An14g02760	**↑**	
endo-glucanase EglB	An16g06800	**↑**	
endo-glucanase similar to *Trichoderma reesei* egl4	An14g02670	**↑**	
alpha-glucanosyltransferase AgtA (GPI-anchored)	An09g03100	**↑**	
chitin synthase ChsL	An02g02340	**↑**	Chs3
chitin transglycosidase	An16g02850	**↑**	Crh1
chitinase	An01g05360	**↑**	Cts2
cell wall protein similar to *A. nidulans* PhiA	An14g01820	**↑**	
cell wall protein with internal repeats	An12g10200	**↑**	
cell wall protein (flocculin)	An12g00140	**↑**	Flo11
protein involved in β-1,3 glucan synthesis	An05g00130	**↓**	Knh1
α-1,2-mannosyltransferase	An04g06730	**↓**	Mnn2
***Primary metabolism***			
isocitrate lyase AcuD	An01g09270	**↑**	Icl1
citrate lyase	An11g00510	**↑**	
citrate lyase	An11g00530	**↑**	
succinate dehydrogenase	An16g07150	**↑**	Osm1
aspartate transaminase, synthesis of glutamate	An16g05570	**↑**	
acetyl-CoA carboxylase, synthesis of fatty acids	An12g04020	**↑**	
homo-isocitrate dehydrogenase, synthesis of lysine	An15g02490	**↓**	Lys12
arginosuccinate synthetase, synthesis of arginine	An15g02340	**↓**	Arg1
acetylornithine aminotransferase, synthesis of arginine	An15g02360	**↓**	Arg8
arginyl-tRNA synthetase	An02g04880	**↓**	
aspartic beta semi-aldehyde dehydrogenase, synthesis of threonine and methionine	An11g09510	**↓**	Hom2
homoserine kinase, synthesis of threonine	An17g02090	**↓**	Thr1
threonine synthase	An16g02520	**↓**	Thr4
phosphoribosylglycinamide formyltransferase, synthesis of purines	An02g02700	**↓**	Ade8
***Secondary metabolism***			
polyketide synthase	An11g07310	**↑**	
similar to plant zeaxanthin epoxidase ABA2	An03g06500	**↑**	
similar to enniatin synthase esyn1 of *Fusarium scirpi*	An13g03040	**↑**	
similar to enoyl reductase LovC of the lovastatin biosynthesis *A. terreus*	An13g02940	**↑**	
similar to enoyl reductase LovC of the lovastatin biosynthesis *A. terreus*	An09g01880	**↑**	
similar to HC-toxin peptide synthase HTS of *Cochliobolus carbonum*	An16g06720	**↑**	
***Transporter***			
polyamine transporter	An11g07300	**↑**	Tpo3
polyamine transporter	An12g07400	**↑**	Tpo3
polyamine transporter	An13g03220	**↓**	Tpo1
vacuolar basic amino acid transporter	An06g00770	**↑**	Vba5
oligopeptide transporter	**An11g01040**	**↓**	**Opt1**
hexose transporter	An02g07610	**↑**	Hxt5
galactose transporter	**An01g10970**	**↓**	**Gal2**
low-affinity Fe(II) transporter of the plasma membrane	**An16g06300**	**↓**	**Fet4**
siderophore transporter	An03g03560	**↓**	Arn1
plasma membrane multidrug transporter	An07g01250	**↑**	Pdr5
multidrug transporter	An13g03060	**↑**	Snq2
***Protein trafficking***			
protein kinase involved in exocytosis	An08g03360	**↑**	Kin1
***Other signaling processes***			
zinc finger transcriptional repressor	An04g08620	**↑**	Oaf3
protein recruiting the SAGA complex to promoters	An07g04540	**↑**	Cti6
histidine kinase osmosensor	An14g02970	**↑**	Sln1
transcriptional regulator involved in nitrogen repression	An02g11830	**↑**	Ure2
transcription factor similar to *A. nidulans* MedA	An02g02150	**↑**	
transcription factor	An02g06180	**↑**	
transcription factor important for salt stress resistance	An12g09020	**↑**	Hal9
DNA damage checkpoint protein during replication	An03g06930	**↓**	Rad24
SUN family protein involved in replication	An08g07090	**↓**	Sim1
transcription factor important for Zn^2+^ homeostasis	An08g01860	**↓**	Zap1
alpha subunit of the translation initiation factor	An18g04650	**↓**	Gcn3
**Others**			
pathogenesis-related protein	An08g05010	↑	Pry1
hypothetical aspergillosis allergen rAsp	**An03g00770**	↓	

Genes up-regulated are indicated with ↑, genes down-regulated with ↓. Differential gene expression was evaluated by moderated t-statistics using the Limma package [82] with a FDR threshold at 0.05 [83]. Identical ORFs which are differentially expressed in P*glaA*-*racA^G18V^* and Δ*racA* are indicated in bold. Fold changes and statistical significance is given in Additional file 1 and 2.

* : Protein functions were predicted based on information inferred from the *Saccharomyces* genome data base SGD (http://www.yeastgenome.org/) and the *Aspergillus* genome database AspGD (http://www.aspergillusgenome.org/).

The transcriptomic fingerprint indicated that the turgor pressure is increased during apolar growth and thereby an osmotic and cell wall stress is sensed in the P*glaA*-*racA^G18V^* strain. An14g02970, an ORF with strong similarity to the Sln1p histidine kinase osmosensor of *S. cerevisiae* which forms a phosphorelay system to activate the Hog1 MAP kinase cascade [44], showed increased expression. In agreement, some cell wall genes which have been shown to respond to caspofungin-induced cell wall stress in *A. niger* [45] were up-regulated as well: *agtA*, a GPI-anchored alpha-glucanosyltransferase and two putative cell wall protein encoding genes *phiA* and An12g10200. In addition, other proteins, known to be up-regulated under cell wall and osmotic stress in *S. cerevisiae* showed enhanced expression in P*glaA*-*racA^G18V^*: An16g02850 (ortholog of the chitin transglycosylase Crh1p, [46], An02g05490 (Ca^2+^/calmodulin dependent protein kinase Cmk2p, [47] and An07g01250 (ortholog of the multidrug transporter Pdr5p, [48]. Furthermore, increased expression was also observed for An03g06500 encoding an ortholog of the plant zeaxanthin epoxidase, which catalyses one step in the biosynthetic pathway of the plant hormone abscisic acid, known to protect plant cells against dehydration under high-salinity stress [49].

The expression of many transporters and permeases (iron, hexoses, amino acids and peptides) was also modulated in P*glaA*-*racA^G18V^* as well as the expression of several amino acid biosynthetic genes, most of which were down-regulated (lysine, arginine, threonine, methionine; Table 4). As also some ORFs predicted to function as important activators in replication (An03g06930, An08g07090) and translation (An18g04650) displayed decreased expression suggests that reduced tip extension during apolar growth slows down basic cellular processes. In this context, it is interesting to note that An01g09260 predicted to break down sphingosin 1-phosphate (S1P) showed increased expression during apolar growth. S1P is a sphingolipid acting as second messenger in lower and higher eukaryotes regulating respiration, cell cycle and translation [50] and is also important for the sole sphingolipid-to-glycerolipid metabolic pathway [51]. As also expression of An02g06440, a predicted ortholog of the *S. cerevisiae* sphingosin flippase Rsb1p which extrudes sphingosin into the extracytoplasmic side of the plasma membrane, was increased during apolar growth might suggest that reduced levels of S1P are present in apolar growing hyphal tips (see discussion).

Interestingly, none of the extracted 148 genes (Table S4) showed any link to actin filament organization, although actin polarization is lost in the P*glaA*-*racA^G18V^* strain and actin patches are randomly scattered intracellularly or at the cell periphery [24]. One explanation might be that transcription of actin-related genes was immediately altered after maltose addition which induced the switch from polar to apolar growth in P*glaA*-*racA^G18V^*. To still get a glimpse on genes involved in actin patch formation, the 2 h dataset of P*glaA*-*racA^G18V^* versus P*glaA*-*racA* was screened for the presence of enriched GO terms related to actin (Table S3). Using this approach, 10 actin-related genes were extracted which are summarized in Table 5. Most interestingly, An18g04590, an ortholog of the *S. cerevisiae* Rho GDP dissociation inhibitor Rdi1p displayed increased expression. Rdi1p regulates the Rho GTPases Cdc42p, Rho1p and Rho4p, localizes to polarized growth sites at specific times of the cell cycle and extracts all three proteins from the plasma membrane to keep them in an inactive cytosolic state [52]–[54]. Overexpression of Rdi1p causes slightly rounder cell morphology in *S. cerevisiae* [55]. Another interesting actin-related gene showing increased expression in strain P*glaA*-*racA^G18V^* is An11g02840, the predicted homolog of the *S. cerevisiae* Slm2 protein. Slm2p binds to the second messenger phosphatidylinositol-4,5-bisphosphate (PIP2) and to the TORC2 signaling complex and integrates inputs from both signaling pathways to control polarized actin assembly and cell growth [56]. In addition, it is also a target of sphingolipid and calcium signaling during heat stress response in *S. cerevisiae* and promotes cell survival by coordinating cell growth and actin polarization [57]. Both An18g04590 (Rdi1p ortholog) and An11g02840 (Slm2p ortholog) might thus be two key proteins important to sustain tip growth and proper actin polarization in *A. niger*. The other eight GO enriched proteins of strain P*glaA*-*racA^G18V^* are orthologs of *S. cerevisiae* proteins with a function in cortical actin patch formation (Table 5). For example, subunits of the Arp2/3 complex which is required for the motility and integrity of cortical actin patches are up-regulated in apolar growing hyphal tips of strain P*glaA*-*racA^G18V^*, one of which (An18g06590, Arc40p ortholog) also responds to caspofungin-induced loss of cell polarity in *A. niger* [45]. Another interesting gene showing increased expression was An04g09020, the ortholog of twinfilin. Twf1p has been shown to localize to cortical actin patches in *S. cerevisiae*, forms a complex with the capping protein Cap2 (An01g05290, up-regulated in P*glaA*-*racA^G18V^*), sequesters actin monomers to sites of actin filament assembly and is regulated by PIP2 [58], providing an additional hint that re-structuring of the actin cytoskeleton in P*glaA*-*racA^G18V^* might be orchestrated by PIP2 signaling. Taken together, the transcriptomic fingerprint of *A. niger* hyphae expressing dominant active RacA suggests that several signaling pathways and secondary messengers might orchestrate the morphological switch from polar to apolar growth.

**Table 5:** GO term enriched actin-related genes whose expression responds to the switch from polar to apolar growth in P*glaA*-*racA^G18V^*.

Predicted protein function*	Open reading frame code	Up/Down	Closest *S. cerevisiae* ortholog
Rho GDP dissociation inhibitor	An18g04590	↑	Rdi1
TORC2 and phosphoinositide PI4,5P(2) binding protein	An11g02840	↑	Slm2
Arp2/3 complex subunit	An02g06360	↑	Arc15
Arp2/3 complex subunit	An01g05510	↑	Arc35
Arp2/3 complex subunit	An18g06590	↑	Arc40
tropomyosin 1	An13g00760	↑	Tpm1
actin cortical patch component	An02g14620	↑	Aip1
twinfilin	An04g09020	↑	Twf1
actin-capping protein	An01g05290	↑	Cap2
protein recruiting actin polymerization machinery	An10g00370	↑	Bzz1

Genes up-regulated are indicated with ↑. Differential gene expression was evaluated by moderated t-statistics using the Limma package [82] with a FDR threshold at 0.05 [83]. Fold changes and statistical significance is given in Additional file 1 and 2.

* : Protein functions were predicted based on information inferred from the *Saccharomyces* genome data base SGD (http://www.yeastgenome.org/) and the *Aspergillus* genome database AspGD (http://www.aspergillusgenome.org/).

### The RacA effector gene set

We finally compared the transcriptomic dataset of Δ*racA* versus wt with the dataset of P*glaA*-*racA^G18V^* versus P*glaA*-*racA* (4 h after maltose addition) to identify those genes whose transcription is generally affected by morphological changes independently whether provoked by RacA inactivation or by RacA hyperactivation. Overall, 38 genes fulfill this criterion (Fig. 6, Table S4) and are summarized in Table 6. The affected gene list covered processes such as (i) (phospho)lipid signaling, (ii) CWI and remodeling, (iii) actin localization, (iv) transport phenomena and (v) protein trafficking. Most interestingly, 12 out of the 38 genes were also differentially expressed during apical branching in *ramosa-1* [25], including two orthologs of diacylglycerol pyrophosphate phosphatase (Dpp1p), which synthesizes the secondary messenger diacylglycerol (DAG), the activator of mammalian and fungal protein kinase C, which in fungi is a component of the CWI pathway localized upstream of the MAP kinase kinase Mkk1/2 (MkkA in *A. niger*; for review see [59]). Targets of the CWI signaling pathway are cell wall remodeling genes, five of which were differentially expressed in RacA hyper- or inactivation strains (Table 6). From these five genes, three are of special importance as these were also effector genes in the hyperbranching mutant *ramosa-1* [25]. Although calcium signaling genes seemed not be among the extracted 38 genes, its indirect involvement might be conceivable. For example, An18g01090 encoding the predicted ortholog of the *S. cerevisiae* phospholipase B (Plb3p) is among this gene set. Plb3p is activated at high concentrations of Ca^2+^ and specifically accepts phosphatidylinositol as a substrate to keep its concentration on the outer membrane leaflet low [60]. Finally, 17 RacA effector genes encode proteins of unknown function, most of which have no predicted orthologs in *S. cerevisiae*. As their function, however, seems to be important for morphological changes in *A. niger*, they are highly interesting candidate genes for future analyses.

**Table 6:** Complete list of genes whose expression respond to hyperbranching in Δ*racA* versus wild-type and to the switch from polar and apolar growth in P*glaA*-*racA^G18V^* versus P*glaA*-*racA* (4 h after induction).

Predicted protein function*	Open reading frame code	Up/Down in P*glaA*-*racA^G18V^* versus P*glaA*-*racA* (4 h)	Up/Down in Δ*racA* versus wt	Closest *S. cerevisiae* ortholog
***(Phospho)lipid metabolism and signaling***				
phospholipase B, synthesis of glycerophosphocholine	An18g01090	↓	↓	Plb3
diacylglycerol pyrophosphate phosphatase, synthesis of DAG	An02g01180	↓	↓	**Dpp1**
diacylglycerol pyrophosphate phosphatase, synthesis of DAG	**An04g03870**	↓	↓	**Dpp1**
sterol 24-C-methyltransferase, ergosterol synthesis	An04g04210	↑	↑	Erg6
C-14 sterol reductase, ergosterol synthesis	**An01g07000**	↓	↓	**Erg24**
transcription factor important for sterol uptake	An02g07950	**↓**	**↓**	Upc2
transcription factor important for sterol uptake	An12g00680	**↓**	**↓**	Upc2
***Cell wall remodeling and integrity***				
α-1,3-mannosyltransferase	An15g04810	↓	↓	Mnt2
endo-mannanase (GPI-anchored), DfgE	**An16g08090**	↓	↓	**Dfg1**
β-1,4-glucanase	**An03g05530**	↓	↓	
cell wall protein	**An11g01190**	**↓**	**↓**	**Sps22**
plasma membrane protein	An02g08030	**↓**	**↓**	Pmp3
***Actin localization***				
amphysin-like protein required for actin polarization	An17g01945	↓	↓	Rvs161
actin-binding protein involved in endocytosis	**An03g01160**	↓	↓	**Lsb4**
***Transporter***				
choline/ethanolamine permease	An01g13290	↓	↓	Hnm1
low-affinity Fe(II) transporter	**An16g06300**	↓	↓	**Fet4**
oligopeptide transporter	An11g01040	↓	↓	**Opt1**
galactose/glucose permease	An01g10970	↓	↓	Gal2
***Protein trafficking***				
GTPase activating protein involved in protein trafficking	An01g02860	↓	↓	Gyp8
protein important for endosomal-vacuolar trafficking	**An11g01810**	↓	↓	**Rcr2**
peptidase	An03g02530	↓	↓	
***Others***				
hypothetical aspergillosis allergen rAsp	**An03g00770**	↓	↓	
cytochrome P450 protein	An11g02990	↓	↓	Dit2
isoamyl alcohol oxidase	An03g06270	↓	↓	
protein with strong similarity to penicillin V amidohydrolase	An12g04630	↓	↓	
oxidoreductase	An03g00280	↑	↑	
protein with nucleotide binding domain	An01g08150	↑	↑	Irc24
protein with methyltransferase domain	An09g00160	↑	↑	
protein with unknown function	**An15g03880**	↓	↓	
protein with unknown function	**An01g10900**	**↓**	**↓**	
protein with unknown function	**An07g05820**	↓	↓	
protein with unknown function	An18g00810	↓	↓	
protein with unknown function	An04g04630	**↓**	**↓**	
protein with unknown function	An06g00320	**↓**	**↓**	
protein with unknown function	An07g04900	↓	↓	
protein with unknown function	An01g13320	↓	↓	
protein with unknown function	An16g07920	↑	↑	
protein with unknown function	An01g03780	↓	↓	

Genes up-regulated are indicated with ↑, genes down-regulated with ↓. Differential gene expression was evaluated by moderated t-statistics using the Limma package [82] with a FDR threshold at 0.05 [83]. Identical ORFs or proteins with predicted similar function being also differentially expressed in *ramosa-1* are indicated in bold. Fold changes and statistical significance is given in Additional file 1 and 2.

* : Protein functions were predicted based on information inferred from the *Saccharomyces* genome data base SGD (http://www.yeastgenome.org/) and the *Aspergillus* genome database AspGD (http://www.aspergillusgenome.org/).

## Discussion

The fungal actin cytoskeleton is highly dynamic and fulfils multiple functions important for cell polarity regulation, endocytosis, exocytosis and septation. Central regulators of actin polymerization and depolymerization are Rho GTPases whose activity is regulated by their membrane-cytoplasmatic shuttling which itself is modulated by external or internal morphogenetic signals. Actin dynamics is thus controlled by a network of signaling pathways that sense and integrate different stimuli [61]. We have recently proposed that the *A. niger* GTPases RacA and CftA (Cdc42p) can substitute each other with respect to actin assembly but that actin disassembly is mainly under control of RacA. A *racA* deletion mutant is thus not affected in actin polymerization (because is secured by CftA) but impaired in actin disassembly. In consequence, maintenance of apical dominance can become frequently lost in the *racA* deletion strain resulting in a hyperbranching phenotype. In contrast, RacA trapped in its active, GTP-bound form (RacA^G18V^) provokes the formation of higher-order actin structures, i.e. actin patches, which cause loss of polarity maintenance and the formation of round, apolar growing cells [24]. The purpose of the current study was to identify the transcriptional signature associated with morphological changes in hyphal tip growth of *A. niger*. The transcriptional response of *A. niger* provoked by inactivation and hyperactivation of RacA, respectively, was determined and compared with the transcriptomic fingerprint of the apical branching transcriptome of the *ramosa-1* mutant [25]. The data obtained allowed us to reconstruct the transcriptomic network that helps *A. niger* to adapt to abnormal morphologies and to secure the integrity of its cell wall.

### A transcriptomic perspective on the morphogenetic network of *A. niger*

**Figure 7:**
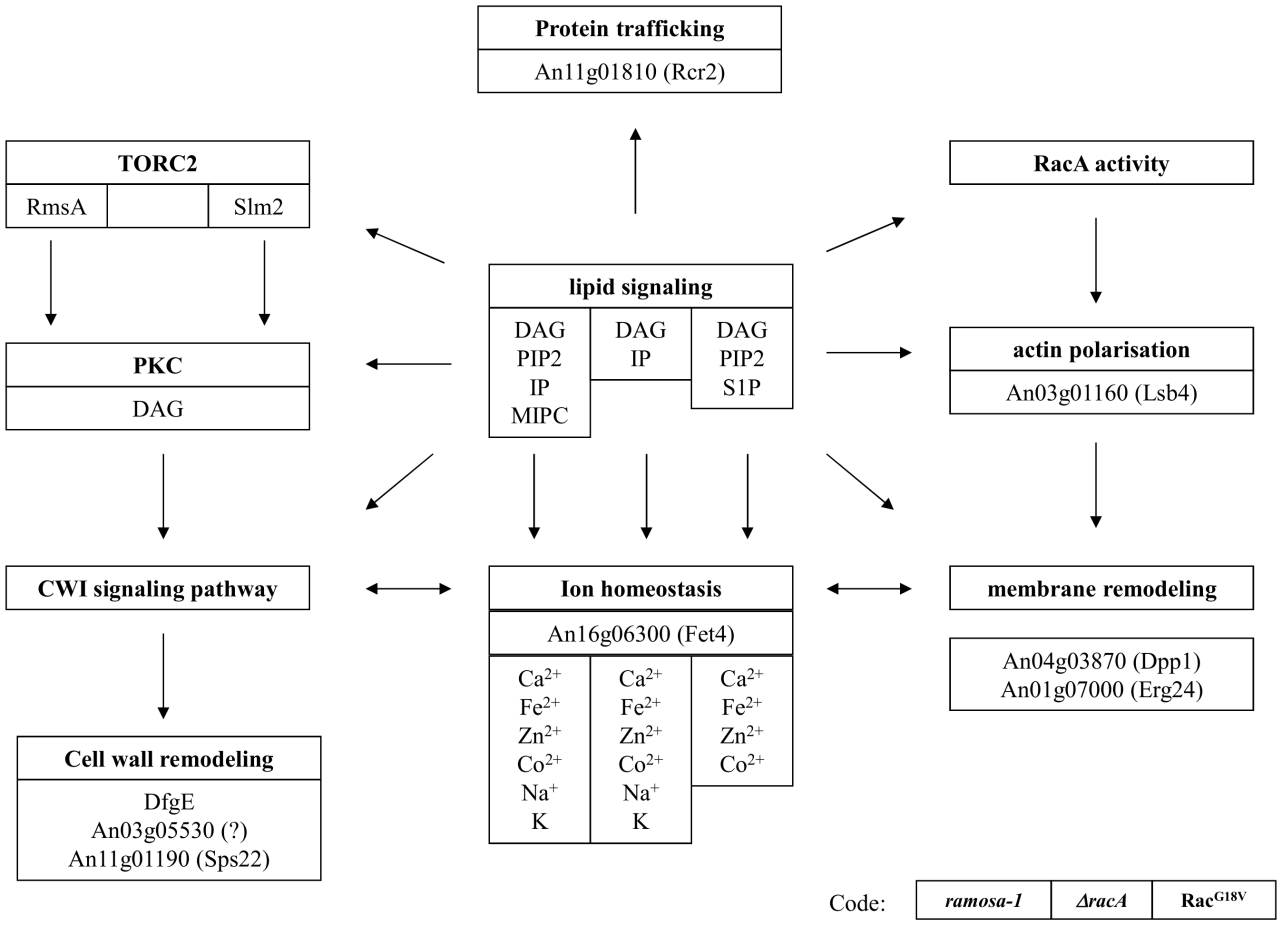
A reconstructed model for the morphogenetic network of *A. niger* based on the transcriptomic fingerprints determined for the apical branching mutant *ramosa-1* [25], the apical branching mutant Δ*racA* (this work) and the apolar growing mutant P*glaA*-RacA^G18V^ (this work). The model also rests on cell biological and phenotypic data obtained for all three strains (this work and [25]) as well as on literature data for conserved mechanisms from yeast to humans (see discussion for references). Indicated are signalling and metabolic processes, which showed transcriptional responses in all three strains, some deduced key players and the hypothetical connection of these processes.

A central result of our comparative transcriptomics approach is the finding that several lipid molecules are likely involved in the maintenance of polar growth in *A. niger* (Fig. 7). The synthesis of important phospho- and sphingolipid molecules (phosphatidic acid, DAG, PIP2, inositolphosphates (IP), glycerophosphocholine, mannose-inositol-phosphoceramide (MIPC) and S1P seem to be modulated during apical branching (Δ*racA*, *ramosa-1*) and apolar growth (P*glaA*-*racA^G18V^*), as genes encoding respective synthetic or degrading enzymes showed differential expression in comparison to the wild-type (Tables 3, 4 and 6). Many of these molecules function as secondary messengers in eukaryotes (DAG, PA, IP, PIP2, S1P), others are essential components of fungal membranes (plasma membrane, organelles, lipid droplets), whereby sphingolipids (e.g. MIPC) and ergosterol are worth highlighting as they concentrate to form lipid rafts in plasma membranes which organize and regulate signaling cascades involved in polar growth control of *S. cerevisiae* [62]. Lipid rafts have been shown to form ordered subdomains of eukaryotic plasma membranes into which monomeric and trimeric G proteins associate in a dynamic and selective manner to organize signal transduction complexes [63]. It is therefore intriguing that expression of An01g07000, the ortholog of the ergosterol synthesizing enzyme Erg24p, is modulated in all three strains Δ*racA*, *ramosa-1* and P*glaA*-*racA^G18V^*, and being also among the cell wall stress responsive genes when *A. niger* is exposed to caspofungin or fenpropimorph [45]. This suggests that ergosterol metabolism is of main importance for polarized growth and cell wall integrity in *A. niger*.

Unfortunately, data on fungal lipid signaling networks are sparse. So far, it is known that sphingolipids play a key role in pathogenicity in *Cryptococcus neoformans*, that the quorum sensing molecule farnesol is involved in mycelial growth, biofilm formation and stress response of *Candida albicans*, that both sphingolipids and farnesol are important for maintaining cell wall integrity and virulence of *A. fumigatus* (for review see [59]) and that the activity of two ceramide synthases is important for the formation of a stable polarity axis in *A. nidulans* [64] (Li et al.). In *Schizosaccharomyces pombe*, MIPC was shown to be required for endocytosis of a plasma-membrane-localized transporter and for protein sorting into the vacuole [65]. As Δ*racA* is affected in endocytosis (see below) and the MIPC synthesizing enzyme Sur1p (An05g02310) is down-regulated as well might suggest that MIPC has a similar function in *A. niger*. Notably, the sphingolipid synthesizing protein inositol-phosphoryl ceramide synthase (Ipc1) plays a major role in both establishment and maintenance of cell polarity in *A. nidulans* by regulating actin dynamics [66], [67]. However, it is not known whether this is mediated by the sphingolipid inositol-phosphoryl ceramide (IPC) or by other products of the ceramide synthetic pathway such as DAG, MIPC or sphingosines. Anyhow, inhibition of sphingolipid synthesis in *A. nidulans* caused wider hyphal cells, abnormal branching and tip splitting and is not suppressible by the addition of sorbitol [66], [67] - observations which also hold true for Δ*racA* and *ramosa-1* [24], [25], suggesting that sphingolipid mediated control of hyphal cell polarity is not mediated by the CWI pathway in *Aspergillus*. Still, *S. cerevisiae* strains defective in CWI signaling (e.g. *pkc1*Δ, *mpk1*Δ) also exhibit severe defects in lipid metabolism, including accumulation of phosphatidylcholine, DAG, triacylglycerol, and free sterols as well as aberrant turnover of phosphatidylcholine, suggesting that CWI signaling and lipid homeostasis are nevertheless closely linked in fungi [68].

A second important outcome of this study is that not only calcium signaling seems to be of utmost importance for morphological decisions in all three mutant strains Δ*racA*, *ramosa-1* and P*glaA*-*racA^G18V^*, but ion homeostasis in general (Fig. 7). Many ion transport proteins are differentially expressed in all three strains when compared to the wild-type situation including transport proteins for Na^+^, K^+^, Ca^2+^, Fe^2+^, Zn^2+^ and Co^2+^. Of special importance is An16g06300, a predicted Fe(II) transporter, homologous to the *S. cerevisiae* plasma membrane transporter Fet4p, whose transcriptional regulation is affected in all three strains. Fet4p is a low-affinity Fe(II) transporter also transporting Zn^2+^ and Co^2+^ and is under combinatorial control of iron (Atf1p transcriptional activator), zinc (Zap1p transcriptional factor) and oxygen (Rox1p repressor) [69], being for example important for *S. cerevisiae* to tolerate alkaline pH [70]. It has been postulated that changes in the phospholipid composition govern the function of membrane-associated zinc transporters such as Fet4p [71]. Vice versa, the transcriptional factor Zap1p controls not only expression of zinc-related transporters but also expression of the DAG pyrophosphate phosphatase Dpp1p [71]. This is a very interesting observation in view of the fact that one predicted Dpp1p ortholog (An04g03870) shows differential expression in all three morphological mutant strains of *A. niger*, an analogy which might suggest that polarized growth of *A. niger* might be orchestrated by (phospho)lipid signaling which is somehow interconnected with zinc metabolism.

Finally, our transcriptomics comparison uncovered that endocytotic processes are likely to be involved in the morphogenetic network of *A. niger*. In all three strains, Δ*racA*, *ramosa-1* and P*glaA*-*racA^G18V^*, expression of An03g01160, a predicted ortholog of the *S. cerevisiae* Lsb4p was modulated (Table 6, Fig. 7). Lsb4p is an actin-binding protein, conserved from yeast to humans, binds to actin patches and promotes actin polymerization together with the WASP protein Lsb17 in an Arp2/3-independent pathway thereby mediating inward movement of vesicles during endocytosis [72]. Lsb4p is also a PIP binding protein due to a phosphoinositide-binding domain (SYLF), which is highly conserved from bacteria to humans. The human homolog of Lsb4p (SH3YL1) binds to PIP3 and couples the synthesis of PIP2 with endocytotic membrane remodeling, whereas Lsb4p binds directly to PIP2 (note that PIP3 is believed to be absent in yeast). Thus, Lsb4p homologs are predicted to couple PIP2 with actin polymerization to regulate actin and membrane dynamics involved in membrane ruffling during endocytosis [73]. Beside An03g01160 (Lsb4p ortholog), An17g01945 is worth highlighting in this context as well. An17g01945 encodes an ortholog of the amphysin-like lipid raft protein Rvs161p and is differentially expressed in both Δ*racA* and P*glaA*-*racA^G18V^*. Rvs161p affects the membrane curvature in *S. cerevisiae* and mediates in conjunction with Rvs167 and PIP2 membrane scission at sites of endocytosis [74].

Taken together, the transcriptomic signature of the three morphological mutants predicts that the morphological changes are brought about the interconnection of several signaling and metabolic pathways. Remarkably, the responding gene set in Δ*racA* and *ramosa-1* seems to be, although substantially overlapping, oppositely regulated. One explanation might be that inactivation of RacA and TORC2 induces dichotomous branching in different manners. As the subcellular distribution of actin is different in both strains (the *ramosa-1* mutant shows scattered actin patches at hyphal tips, whereas actin is concentrated at hyphal apices in the Δ*racA* mutant) might suggest that different causes (loss of actin polarization/actin hyperpolarization) provoke different responding transcriptional changes, which, however, eventually result in the same phenotypic response, namely tip splitting. Similarly puzzling is the observation that the core set of 38 genes which are responsive in both Δ*racA* and P*glaA*-*racA^G18V^* respond in the same direction (Table 6), although they are associated with excessive polar growth (hyperbranching) in the Δ*racA* strain but with the absence of polar growth (tip swelling) in strain P*glaA*-*racA^G18V^*. A plausible explanation might be that loss of polarity maintenance in both strains is connected with similar transcriptional changes controlling actin dynamics and vesicle flow, but that reestablishment of polar growth in the *racA* deletion strain requires genes which are not important for tip swelling in the P*glaA*-*racA^G18V^* strain.

### Does a hyperbranching strain secrete more proteins?

In filamentous fungi, it is believed that protein secretion occurs at the hyphal tip. This holds true for glucoamylase (GLA), the most abundant secreted enzyme in *A. niger* [11], [12]. Another example is α-amylase, the major secretory protein of *A. oryzae* [14]. Hence, one might expect that a higher branching frequency would result in higher secretion yields. However, our study demonstrated that more tips in the Δ*racA* strain do not necessarily increase protein secretion; instead, protein yields were the same in both mutant and wild-type (Table 2). The most logical explanation is that the same amount of secretory vesicles is merely distributed to more tips in the Δ*racA* strain, resulting in less secretory vesicles per individual tip. The quantitative data obtained for the exocytotic marker GFP-SncA and the endocytotic marker AbpA-CFP and SlaB-YFP clearly demonstrate that fewer vesicles are transported to the apex of an individual tip (Fig. 3) and that endocytosis is slowed down as well – the endocytotic ring seems to be less well defined and the fluorescence intensity of both endocytotic markers is decreased (Fig. 4). This data is corroborated by the transcriptomic fingerprint of the Δ*racA* strain. The transcription of genes predicted to function in protein trafficking and actin localization is down-regulated as well as expression of genes governing phospholipid signaling and cell wall remodeling. Remarkably, biomass formation is the same in both Δ*racA* and wt. This suggests that the amount of secreted vesicles is adjusted in both strains just to ensure hyphal tip growth but that the capacity of a hyphal tip growing apparatus to accommodate vesicles is much higher (at least in Δ*racA*). Hence, challenging the Δ*racA* strain to overexpress a certain protein of interest might increase the number of secretory vesicles thus resulting in higher secretion yields. We currently run respective experiments to test this hypothesis. In any case, the hyperbranching Δ*racA* mutant could already be of value for high-density cultivation during industrial processes: it forms a less shear stress-sensitive, compact macromorphology but does not form pellets. It thus exhibits improved rheological properties without any apparent disadvantages with respect to growth rate and physiology.

## Conclusions

The transcriptomic signature of the three individual mutants Δ*racA*, *ramosa-1* and P*glaA*-*racA^G18V^* uncovered specific and overlapping responses to the morphological changes induced and suggests the participation as well as interconnectedness of several regulatory and metabolic pathways in these processes. The data obtained predict a role for different signaling pathways including phospholipid signaling, sphingolipid signaling, TORC2 signaling, calcium signaling and CWI signaling in the morphogenetic network of *A. niger*. These pathways likely induce different physiological adaptations including changes in sterol, zinc and amino acid metabolism and changes in ion transport and protein trafficking. Central to the morphological flexibility of *A. niger* is the actin cytoskeleton whose dynamics can be precisely controlled in these mutants. Future attempts are necessary to address important issues which cannot be resolved by transcriptomics. For example, how is the lipid composition in apical and subapical regions of *A. niger* hyphae? Which morphogenetic proteins are also parts of the network whose expression is regulated post-transcriptionally and can thus not be detected by transcriptomics approaches? Where are they localized during (a)polar growth? What are the metabolic prerequisites to sustain fast polar growth coupled with high secretion rates? Clearly, a comprehensive understanding of the morphogenetic network of *A. niger* will require and integrated systems biology approach where transcriptomics analyses will be combined with proteomics, metabolomics and lipidomics approaches and linked with cell biological studies.

## Materials and Methods

### Strains, culture conditions and molecular techniques

*Aspergillus* strains used in this study are given in Table 7. Strains were grown on minimal medium (MM) [75] containing 1% (w v^−1^) glucose and 0.1% (w v^−1^) casamino acids or on complete medium (CM), containing 0.5% (w v^−1^) yeast extract in addition to MM. When required, plates were supplemented with uridine (10 mM). Transformation of *A. niger* and fungal chromosomal DNA isolation was performed as described [76]. All molecular techniques were carried out as described earlier [77].

**Table 7:** Strains used in this work.

Strain	Relevant genotype	Source
N402	*cspA1* (derivative of ATCC9029)	[86]
AB4.1	*pyrG^−^*	[87]
MA70.15	Δ*kusA pyrG^−^* (derivative of AB4.1)	[88]
MA80.1	Δ*kusA*, Δ*racA::AopyrG*	[24]
FG7	Δ*kusA pyrG^+^ egfp::sncA* (derivative of MA70.15)	Kwon et al, submitted
MA1.8	P*glaA::racA* (derivative of AB4.1)	[24]
MA60.15	P*glaA::racAG18V* (derivative of AB4.1)	[24]
MK5.1	Δ*kusA, slaB::eyfp* (derivative of MA70.15)	This work
MK6.1	Δ*kusA, abpA::ecfp* (derivative of MA70.15)	This work
MK7.1	Δ*kusA*, Δ*racA, slaB::eyfp* (derivative of MA80.1)	This work
MK8.1	Δ*kusA*, Δ*racA, abpA::ecfp* (derivative of MA80.1)	This work

### Bioreactor cultivation conditions

Maltose-limited batch cultivation was initiated by inoculation of 5 L (kg) ammonium based minimal medium with conidial suspension to give 10^9^ conidia L^−1^. Maltose was sterilized separately from the MM and final concentration was 0.8% (w/v). Temperature of 30°C and pH 3 were kept constant, the latter by computer controlled addition of 2 M NaOH or 1 M HCl, respectively. Acidification of the culture broth was used as an indirect growth measurement [78]. Submerged cultivation was performed with 6.6 L BioFlo3000 bioreactors (New Brunswick Scientific, NJ, USA). A more detailed description of the fermentation medium and cultivation is given in [79]. Batch cultivation for P*glaA*-RacA^G18V^ or P*glaA*-RacA were run similarly as the maltose-limited batch cultivations of Δ*racA* cultures except that 0.75% xylose was used as a initial carbon source instead of maltose. When the exponential growth phase was over (indicated by a sharp rise of the dissolved oxygen tension and the pH value), 0.75% maltose was added to induce expression of P*glaA*-RacA^G18V^ or P*glaA*-RacA, respectively. Samples for the analysis of morphological characteristics, biomass formation, protein yield and RNA were taken every hour.

### Analysis of culture broth

Dry weight biomass concentration was determined by weighing lyophilized mycelium separated from a known mass of culture broth. Culture broth was filtered through GF/C glass microfiber filters (Whatman). The filtrate was collected and frozen for use in solute analyses. The mycelium was washed with demineralised water, rapidly frozen in liquid nitrogen and lyophilized. Glucose concentration was measured as previously described [80] with slight modifications: 250 mM triethanolamine (TEA) was used as buffer (pH 7.5). Extracellular protein concentration was determined using the Quick Start Bradford Protein Assay (Bio-Rad) using BSA as standard. The total organic carbon in the culture filtrate was measured with a Total Organic Carbon Analyzer (TOC-Vcsn; Shimadzu, Japan) using glucose as standard.

### Microarray analysis

Total RNA extraction, RNA quality control, labeling, Affymetrix microarray chip hybridization and scanning were performed as previously described [25]. Background correction, normalization and probe summarization steps were performed according to the default setting of the robust multi-array analysis (RMA) package as recently described [81]. Differential gene expression was evaluated by moderated t-statistics using the Limma package [82] with a threshold of the Benjamini and Hochberg False Discovery Rate (FDR) of 0.05 [83]. Fold change of gene expression from different samples was calculated from normalized expression values. Geometric means of the expression values as well as fold change for all strains and comparisons are summarized in Tables S1 and S2 and have been deposited at the GEO repository (http://www.ncbi.nlm.nih.gov/geo/info/linking.html) under the accession number GSE42258. Transcriptomic data for the exponential growth phase of the reference strain N402 was published recently [84].

### Gene Ontology (GO) and enrichment analysis

Over-represented GO terms in sets of differentially expressed genes were determined by Fisher's exact test [85] as implemented in FetGOat [43] using a FDR of q<0.05. An improved GO annotation for *A. niger* CBS513.88 was applied based on ontology mappings from *A.nidulans* FGSCA4 [43].

### Construction of AbpA-CFP and SlaB-YFP expression cassettes

Standard PCR and cloning procedures were used for the generation of the constructs [77]. All PCR amplified DNA sequences and cloned fragments were confirmed by DNA sequencing (Macrogene). Primers used in this study are listed in Table S5. Correct integrations of constructs in *A. niger* were verified by Southern analysis [77].The expression vectors, AbpA-CFP and SlaB-YFP were constructed using the fusion PCR approach as described previously [23] with slight modifications. Plasmid pVM3-1 [23] harboring the GA_5_ peptide linker followed by the CFP, T_trpC_ and the selection marker *pyrG* from *A. oryzae* was used as starting point. A second T_trpC_ terminator sequence was generated by PCR and ligated via a *Sal*I restriction site into pVM3-1 which would later allow looping out of the *pyrG* marker by FOA counter-selection [76]. The resulting plasmid was named pMK3. For the fusion PCR, three separate fragments were amplified by PCR: the C-terminal part of *abpA* ORF (∼1 kb), the module containing CFP-T*_trpc_*-*AopyrG* (∼3.5 kb) and the terminator region of *abpA* (∼1 kb). Subsequently, the three individual fragments were fused together by a fusion PCR and the resulting amplicon (∼5.6 kb) was cloned into pJET (Fermentas) to give plasmid pMK5. SlaB-YFP was also constructed in a similar way and the final plasmid was named pMK6.

### Microscopy

Light microscopic pictures were captured using an Axioplan 2 (Zeiss) equipped with a DKC-5000 digital camera (Sony). For light and fluorescence images for SlaB-YFP and AbpA-CFP transformants, pictures were captured with 40× C-apochromatic objective on an inverted LSM5 microscope equipped with a laser scanning confocal system (Zeiss Observer). The observation conditions for the life-imaging of hyphae were described previously [24]. To determine branching frequencies, the lengths of hyphae and branches were measured and evaluated using the program Image J. For the quantification of GFP-SncA, AbpA-CFP and SlaB-YFP signals, a single section of individual hyphal tip was captured (n>20).

## Supporting Information

Table S1 Complete transcriptome data containing RMA expression values (log2 scale), mean expression values, p-values, q-values and fold changes.
(XLSX)

Table S2 Subset of the transcriptome data containing up-down-regulated gene sets.
(XLSX)

Table S3 ZIP archive file containing FetGOat enrichment results for the up- and down-regulated gene sets of all six comparisons.
(ZIP)

Table S4 Subset of the transcriptome data for selected intersection of the Venn diagram.
(XLSX)

Table S5 Primers used in this study.
(DOCX)
