# Correction: The Transcriptomic Signature of RacA Activation and Inactivation Provides New Insights into the Morphogenetic Network of *Aspergillus niger*

**DOI:** 10.1371/annotation/ffb09608-0f66-4985-97a9-3da9d498b3bf

**Published:** 2014-01-10

**Authors:** Min Jin Kwon, Benjamin M. Nitsche, Mark Arentshorst, Thomas R. Jørgensen, Arthur F. J. Ram, Vera Meyer

Supplementary Table S3 is incorrect. Please view the correct Supplementary Table S3 here: http://plosone.org/corrections/pone.0068946.s003.cn.zip

