# Supplementary figures and images for: The Transcriptomic Signature of RacA Activation and Inactivation Provides New Insights into the Morphogenetic Network of Aspergillus niger
Source: PLoS One. 2013 Jul 24;8(7):e68946. doi: 10.1371/journal.pone.0068946 (PMC3722221; doi:10.1371/journal.pone.0068946)

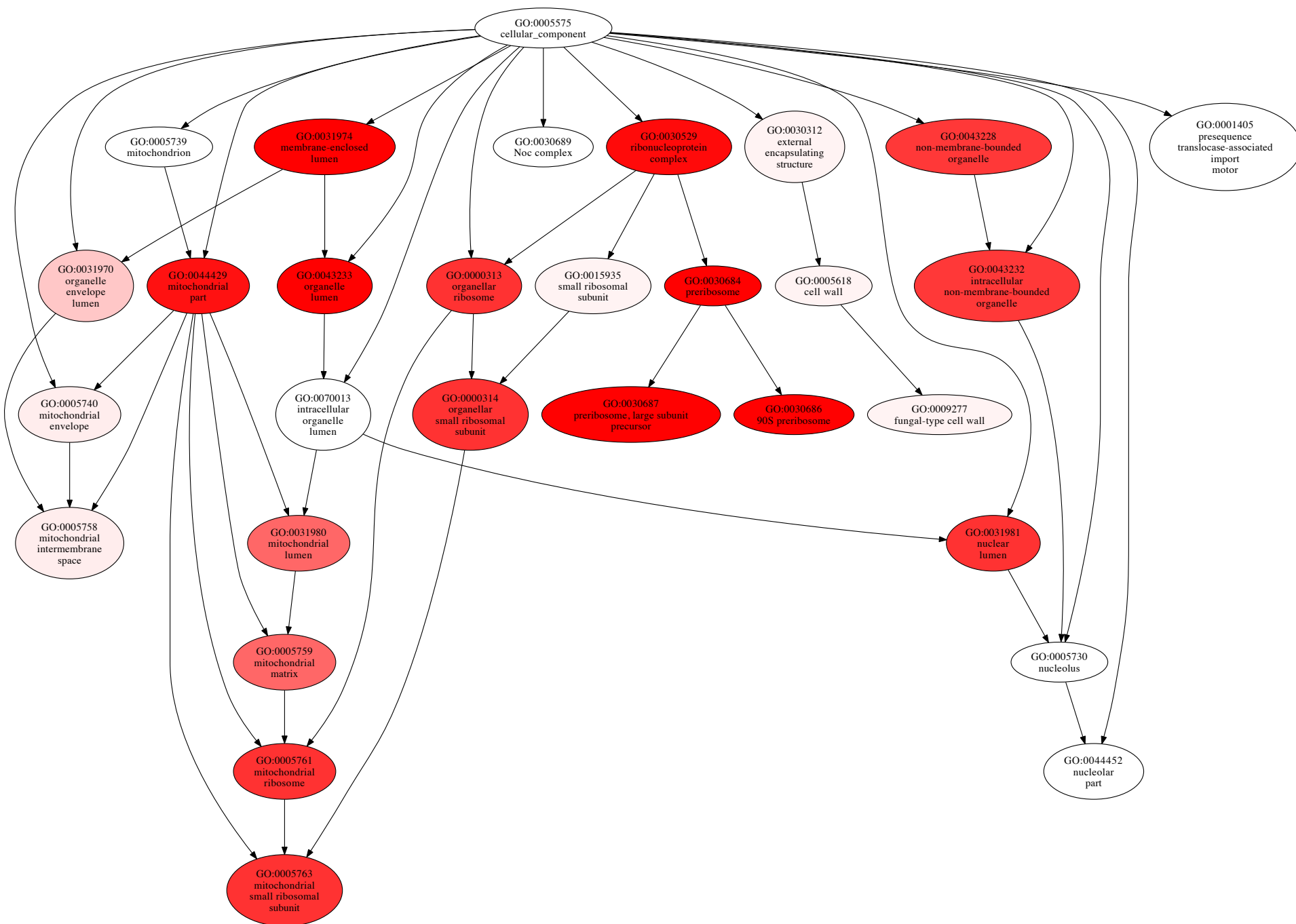

Supplement: Table S3 — ZIP archive file containing FetGOat enrichment results for the up- and down-regulated gene sets of all six comparisons. (ZIP) [file pone.0068946.s003.zip › FetGOat/04.DOWN/plots/04.down.txt_CCover.pdf]

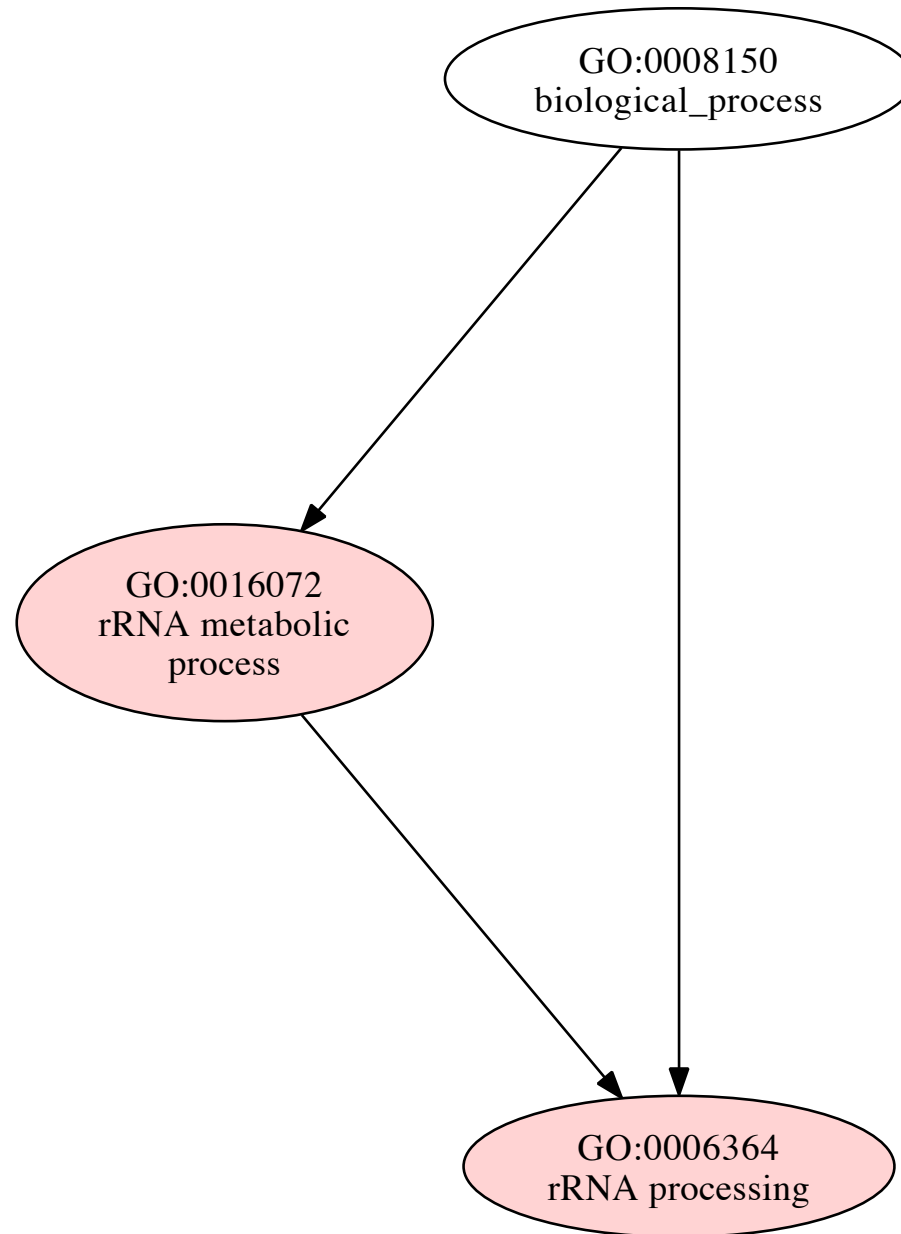

Supplement: Table S3 — ZIP archive file containing FetGOat enrichment results for the up- and down-regulated gene sets of all six comparisons. (ZIP) [file pone.0068946.s003.zip › FetGOat/04.DOWN/plots/04.down.txt_BPover.pdf]

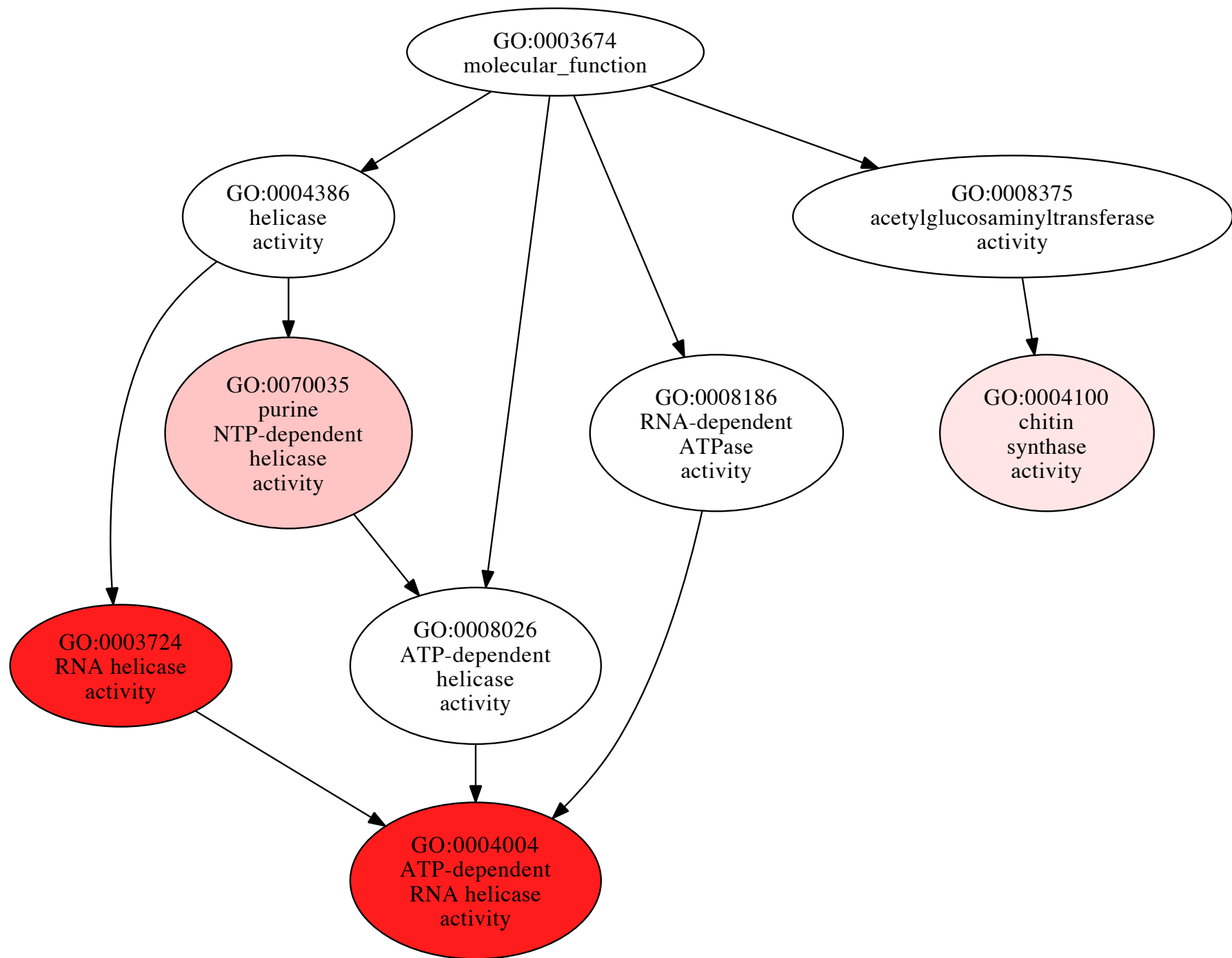

Supplement: Table S3 — ZIP archive file containing FetGOat enrichment results for the up- and down-regulated gene sets of all six comparisons. (ZIP) [file pone.0068946.s003.zip › FetGOat/04.DOWN/plots/04.down.txt_MFover.pdf]

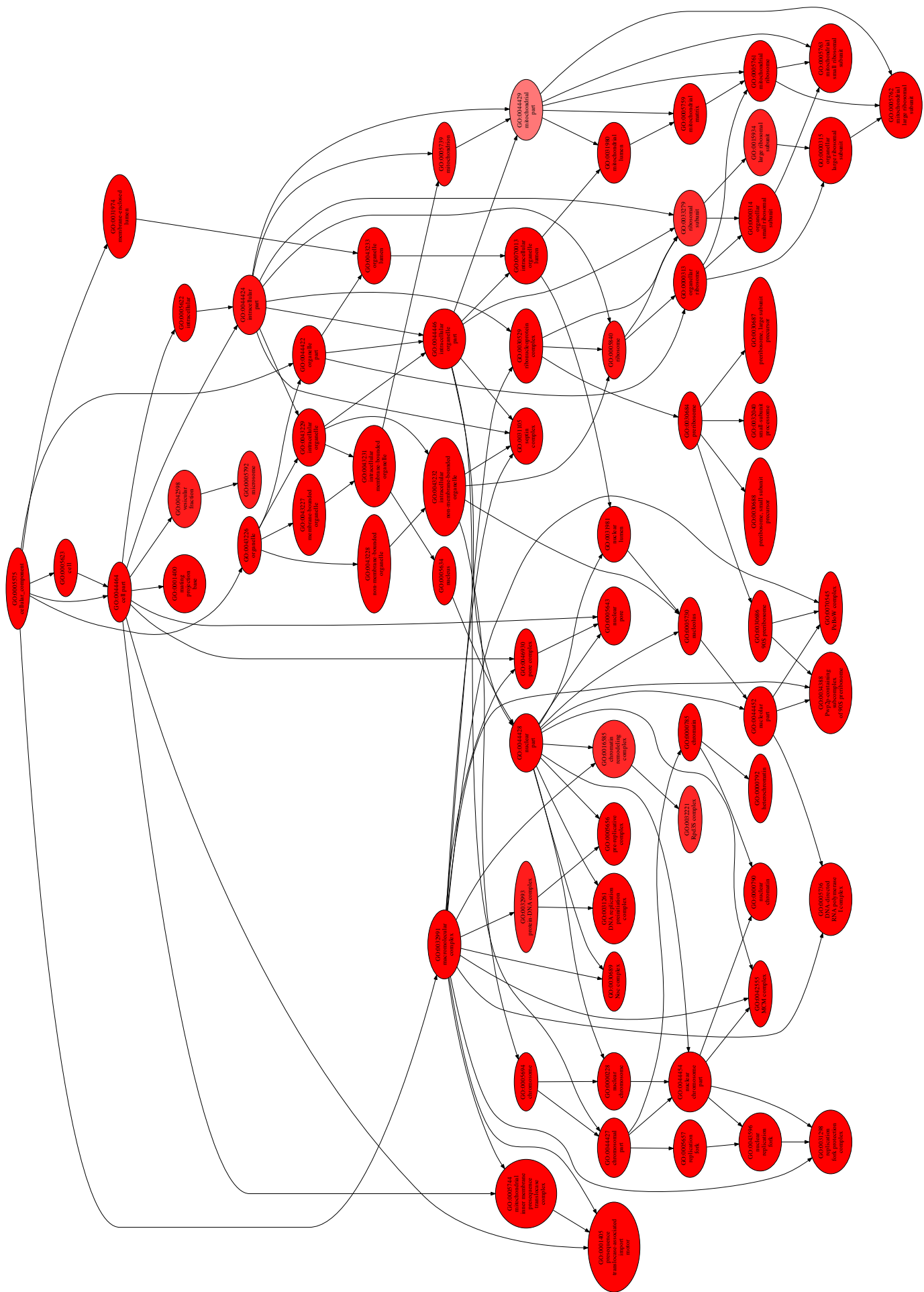

Supplement: Table S3 — ZIP archive file containing FetGOat enrichment results for the up- and down-regulated gene sets of all six comparisons. (ZIP) [file pone.0068946.s003.zip › FetGOat/01.DOWN/plots/01.down.txt_CCover.pdf]

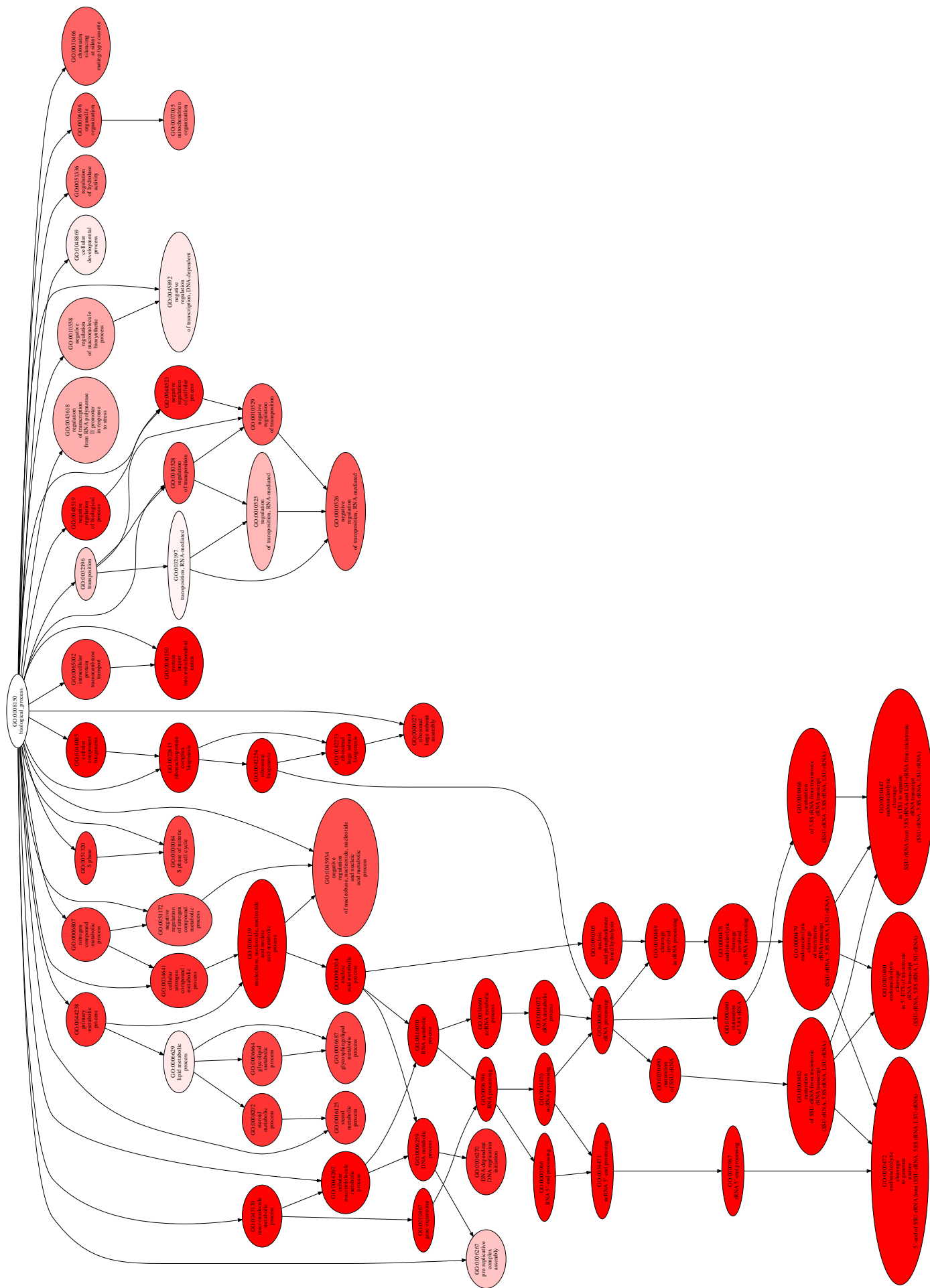

Supplement: Table S3 — ZIP archive file containing FetGOat enrichment results for the up- and down-regulated gene sets of all six comparisons. (ZIP) [file pone.0068946.s003.zip › FetGOat/01.DOWN/plots/01.down.txt_BPover.pdf]

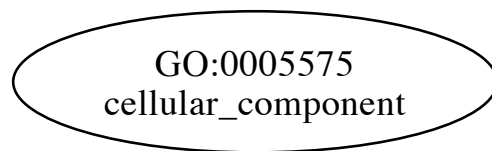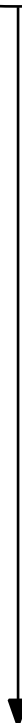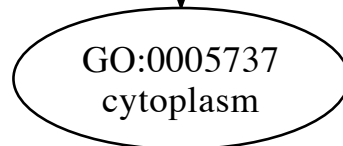

Supplement: Table S3 — ZIP archive file containing FetGOat enrichment results for the up- and down-regulated gene sets of all six comparisons. (ZIP) [file pone.0068946.s003.zip › FetGOat/05.UP/plots/05.up.txt_CCover.pdf]

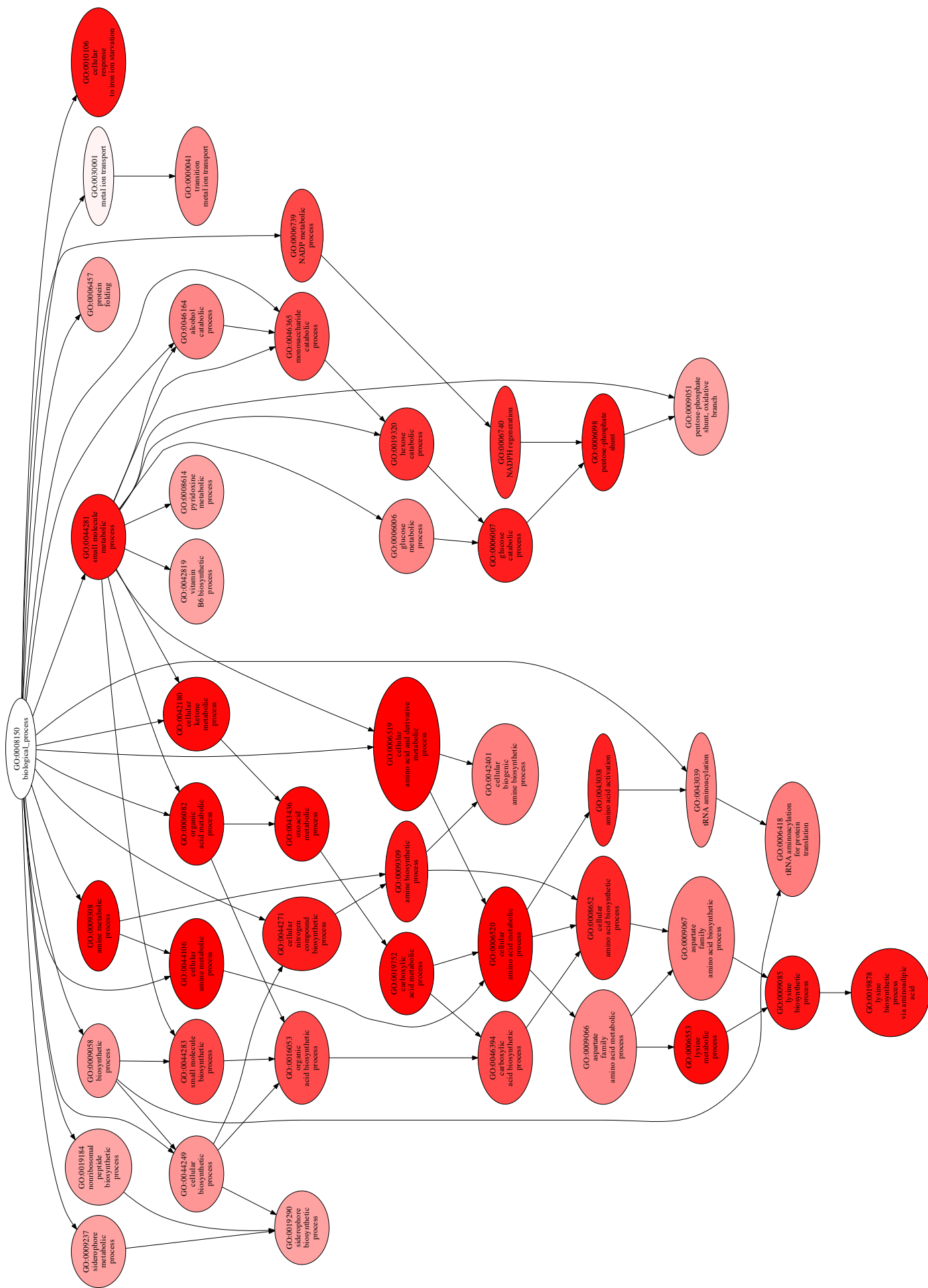

Supplement: Table S3 — ZIP archive file containing FetGOat enrichment results for the up- and down-regulated gene sets of all six comparisons. (ZIP) [file pone.0068946.s003.zip › FetGOat/05.UP/plots/05.up.txt_BPover.pdf]

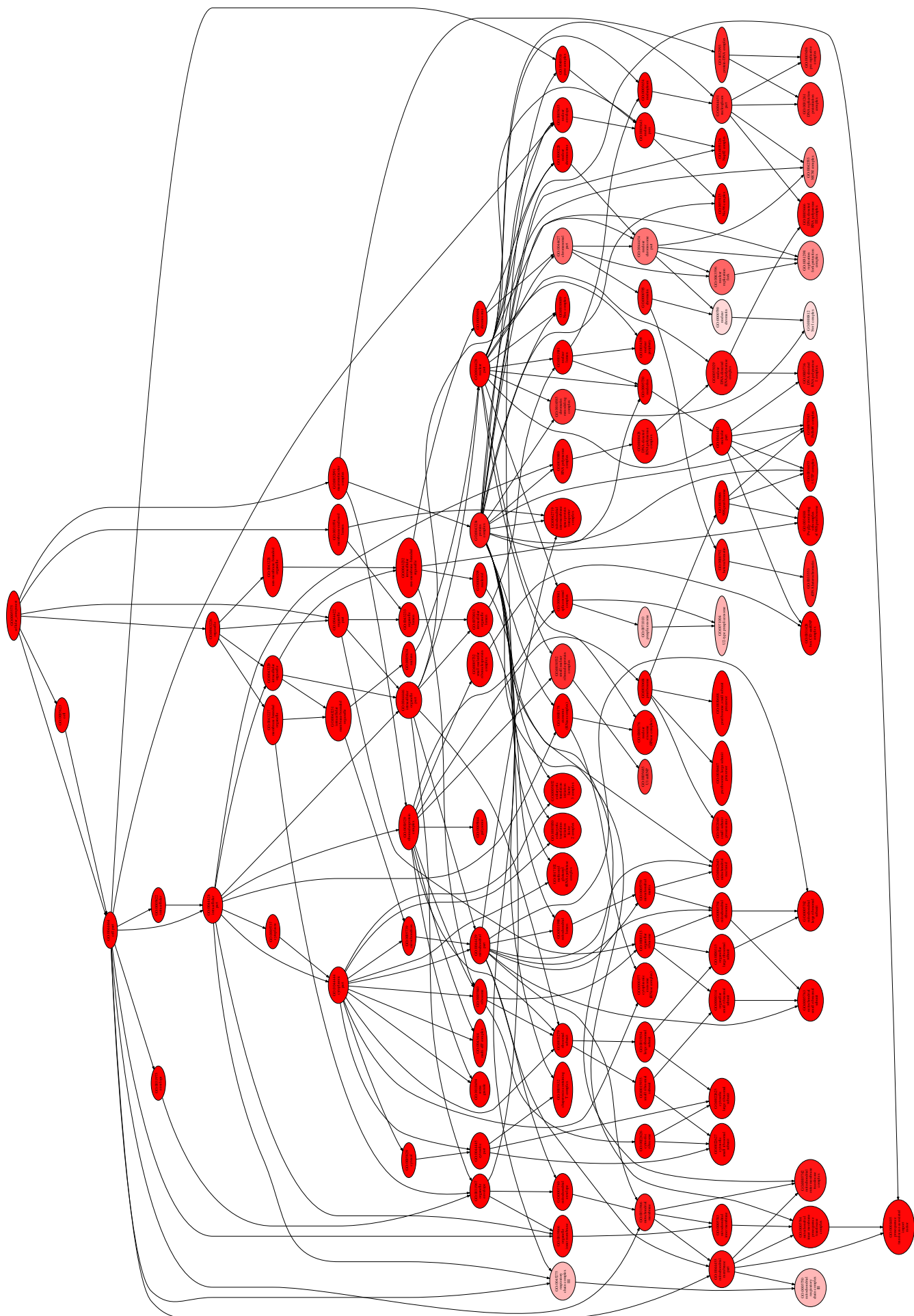

Supplement: Table S3 — ZIP archive file containing FetGOat enrichment results for the up- and down-regulated gene sets of all six comparisons. (ZIP) [file pone.0068946.s003.zip › FetGOat/04.UP/plots/04.up.txt_CCover.pdf]

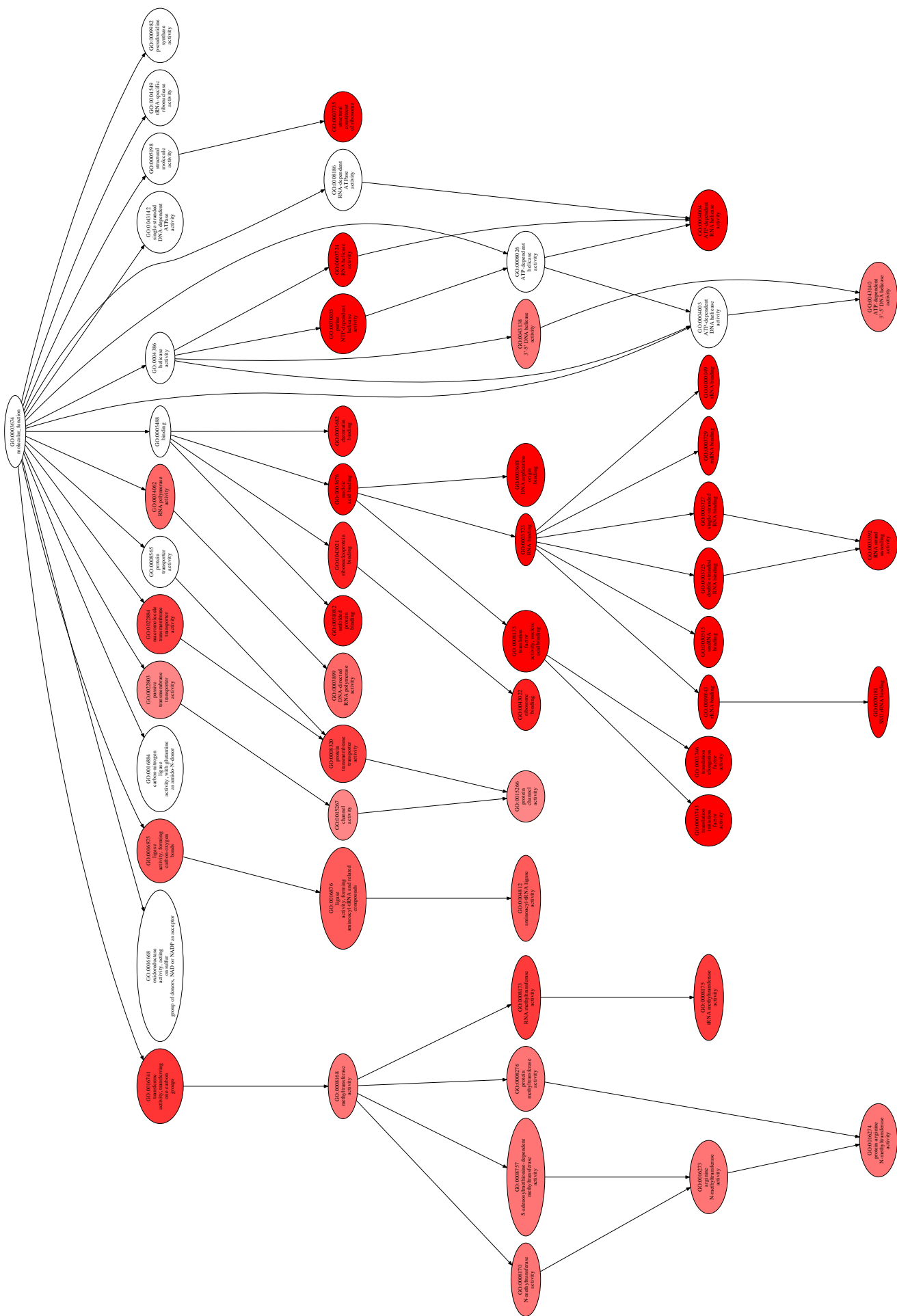

Supplement: Table S3 — ZIP archive file containing FetGOat enrichment results for the up- and down-regulated gene sets of all six comparisons. (ZIP) [file pone.0068946.s003.zip › FetGOat/04.UP/plots/04.up.txt_MFover.pdf]

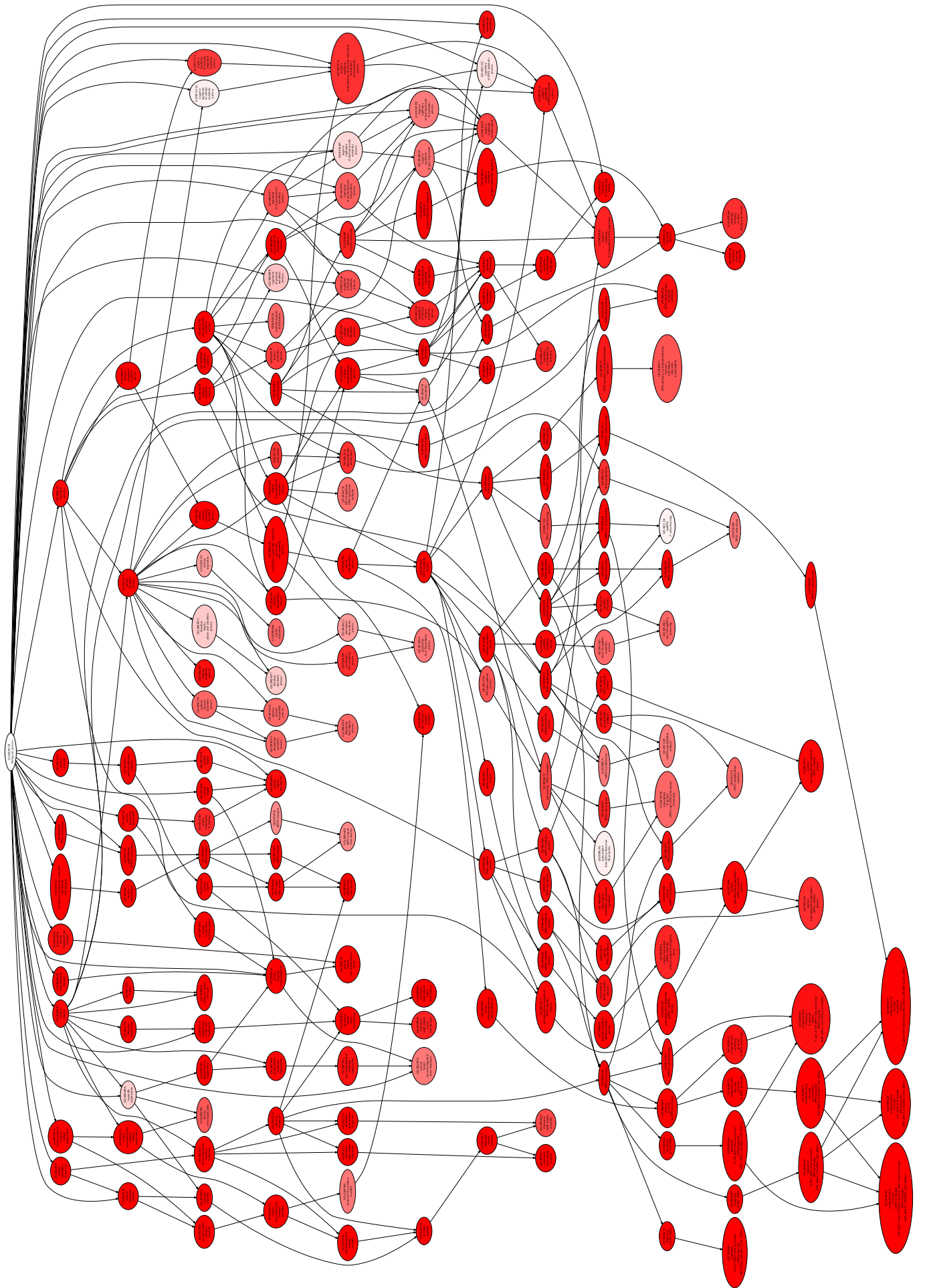

Supplement: Table S3 — ZIP archive file containing FetGOat enrichment results for the up- and down-regulated gene sets of all six comparisons. (ZIP) [file pone.0068946.s003.zip › FetGOat/04.UP/plots/04.up.txt_BPover.pdf]

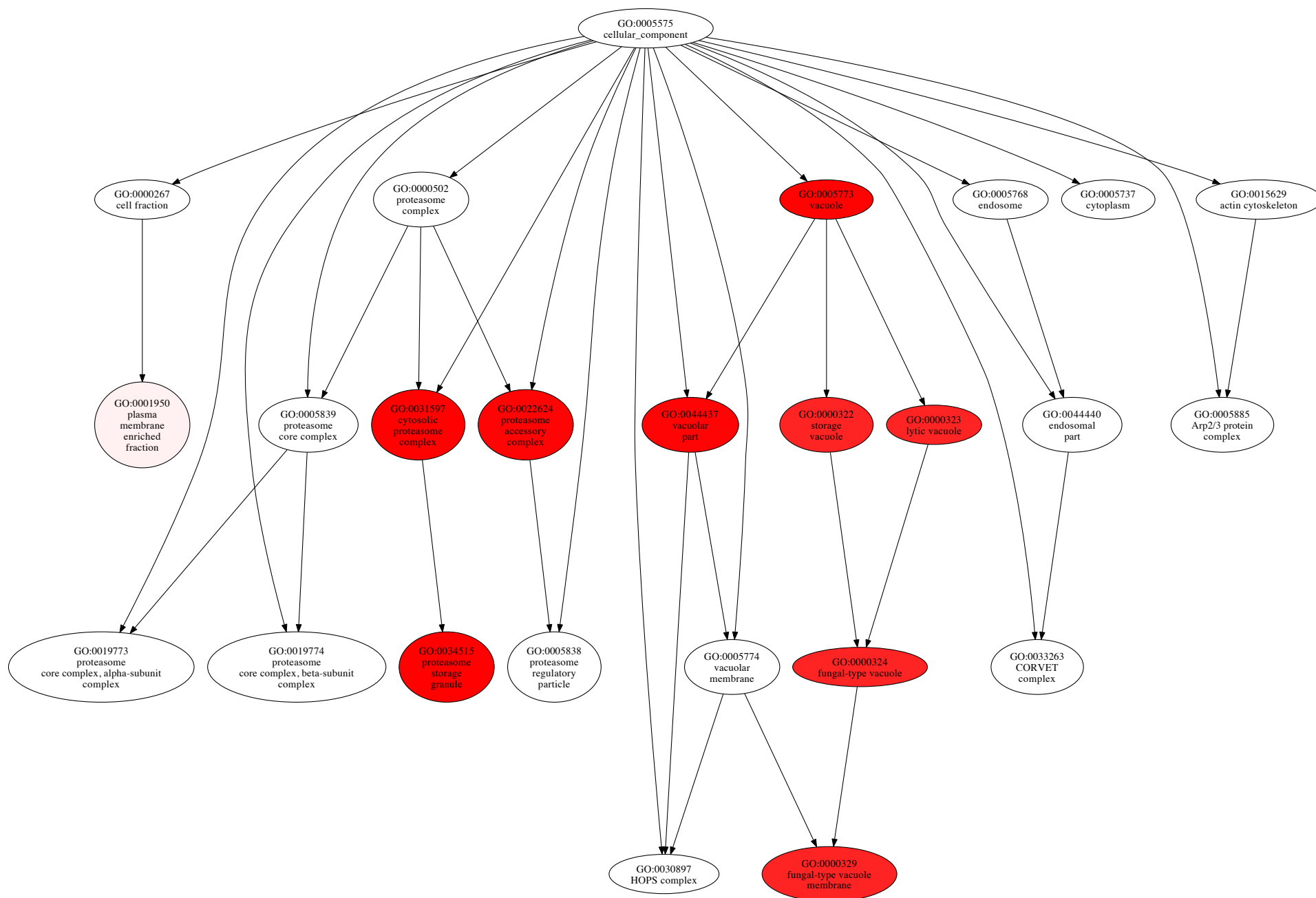

Supplement: Table S3 — ZIP archive file containing FetGOat enrichment results for the up- and down-regulated gene sets of all six comparisons. (ZIP) [file pone.0068946.s003.zip › FetGOat/06.UP/plots/06.up.txt_CCover.pdf]

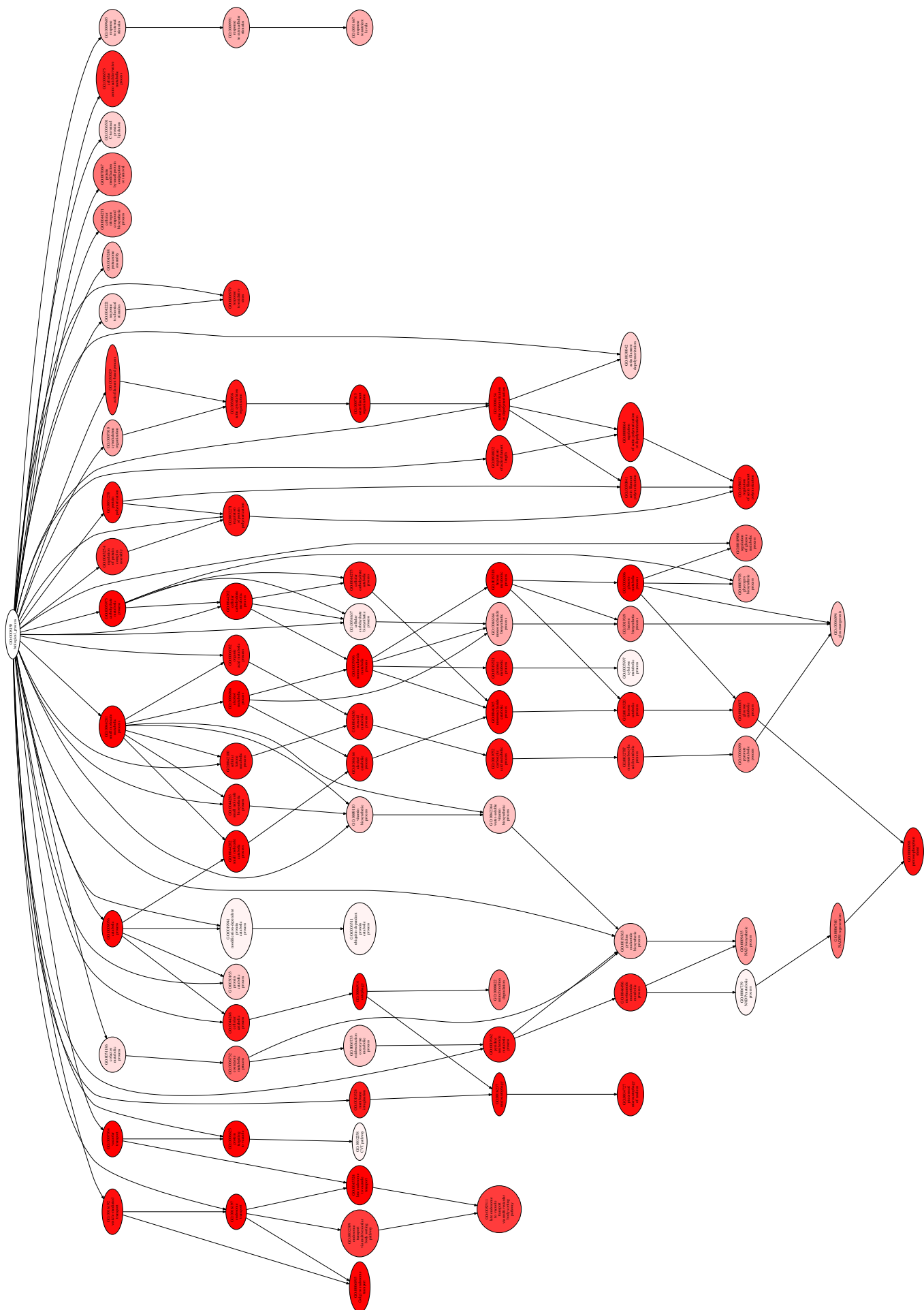

Supplement: Table S3 — ZIP archive file containing FetGOat enrichment results for the up- and down-regulated gene sets of all six comparisons. (ZIP) [file pone.0068946.s003.zip › FetGOat/06.UP/plots/06.up.txt_BPover.pdf]

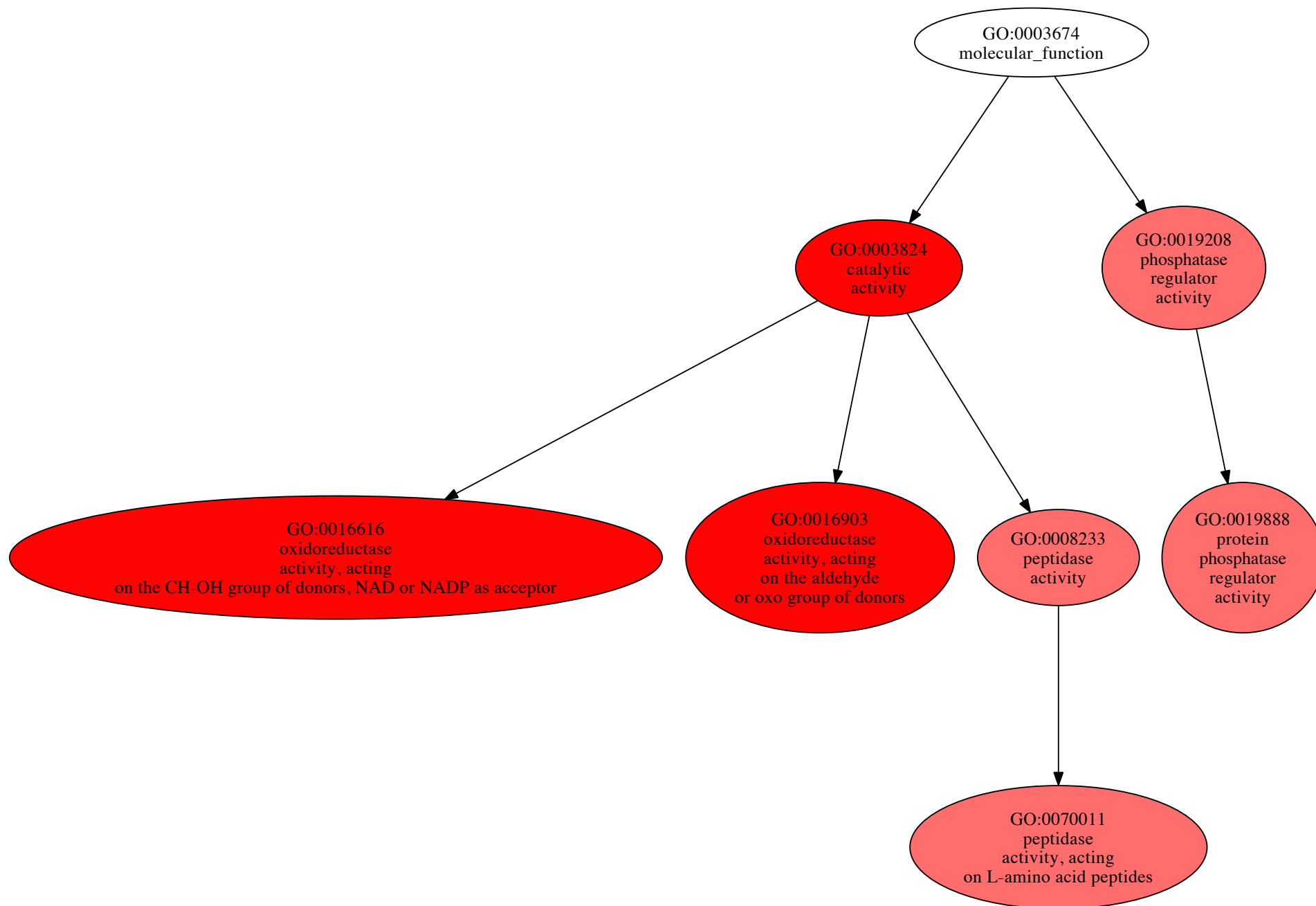

Supplement: Table S3 — ZIP archive file containing FetGOat enrichment results for the up- and down-regulated gene sets of all six comparisons. (ZIP) [file pone.0068946.s003.zip › FetGOat/03.DOWN/plots/03.down.txt_MFover.pdf]

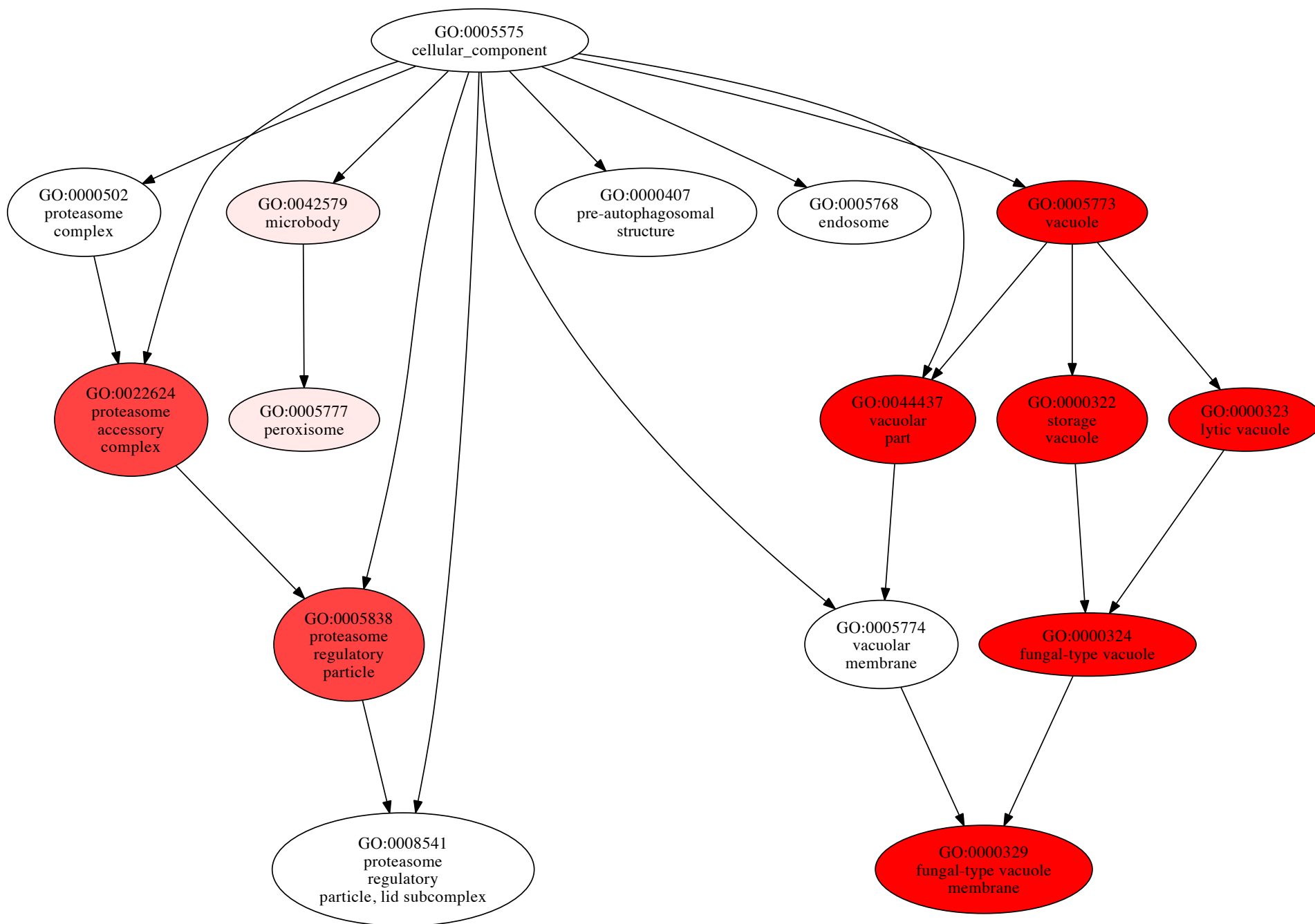

Supplement: Table S3 — ZIP archive file containing FetGOat enrichment results for the up- and down-regulated gene sets of all six comparisons. (ZIP) [file pone.0068946.s003.zip › FetGOat/03.DOWN/plots/03.down.txt_CCover.pdf]

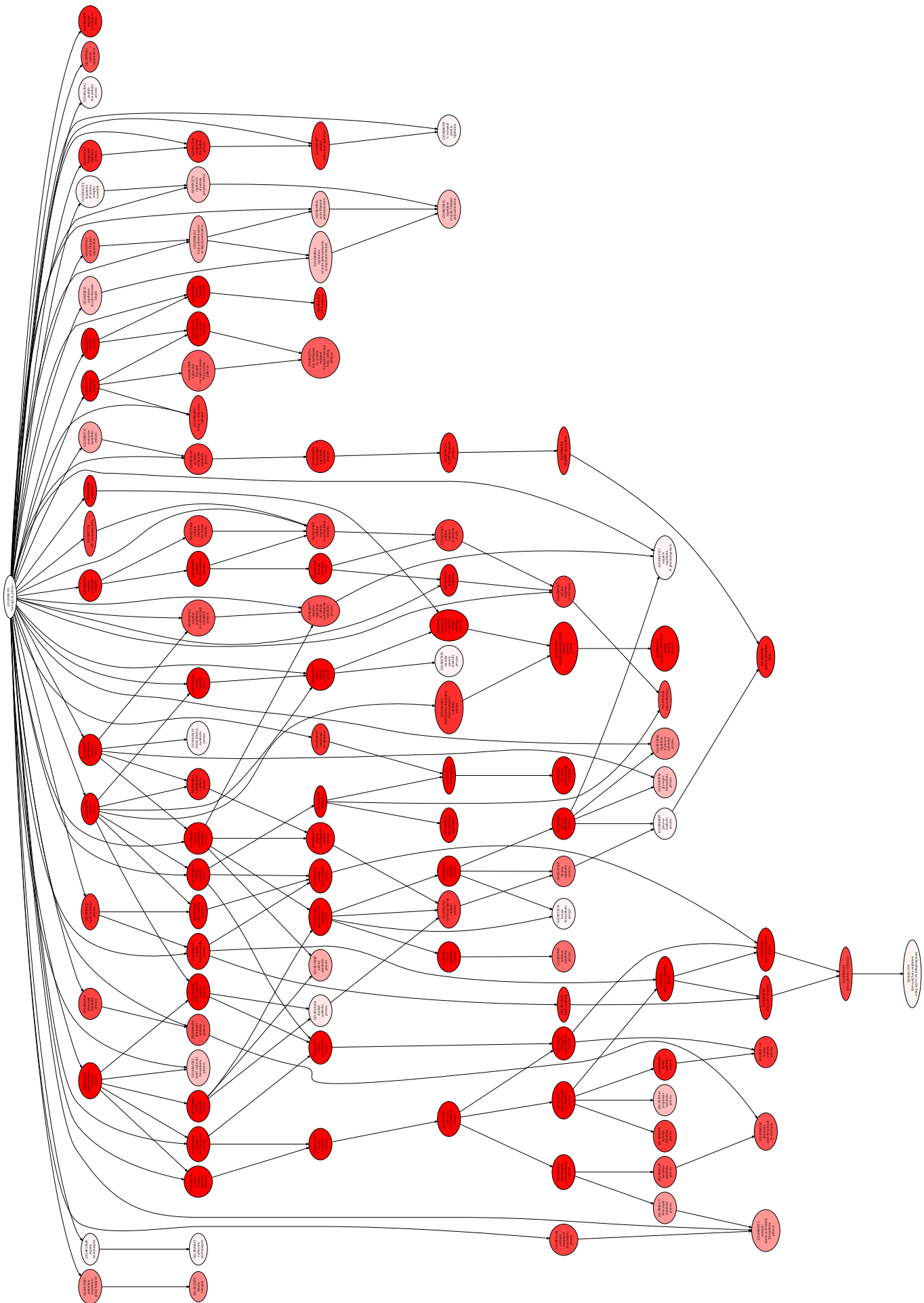

Supplement: Table S3 — ZIP archive file containing FetGOat enrichment results for the up- and down-regulated gene sets of all six comparisons. (ZIP) [file pone.0068946.s003.zip › FetGOat/03.DOWN/plots/03.down.txt_BPover.pdf]

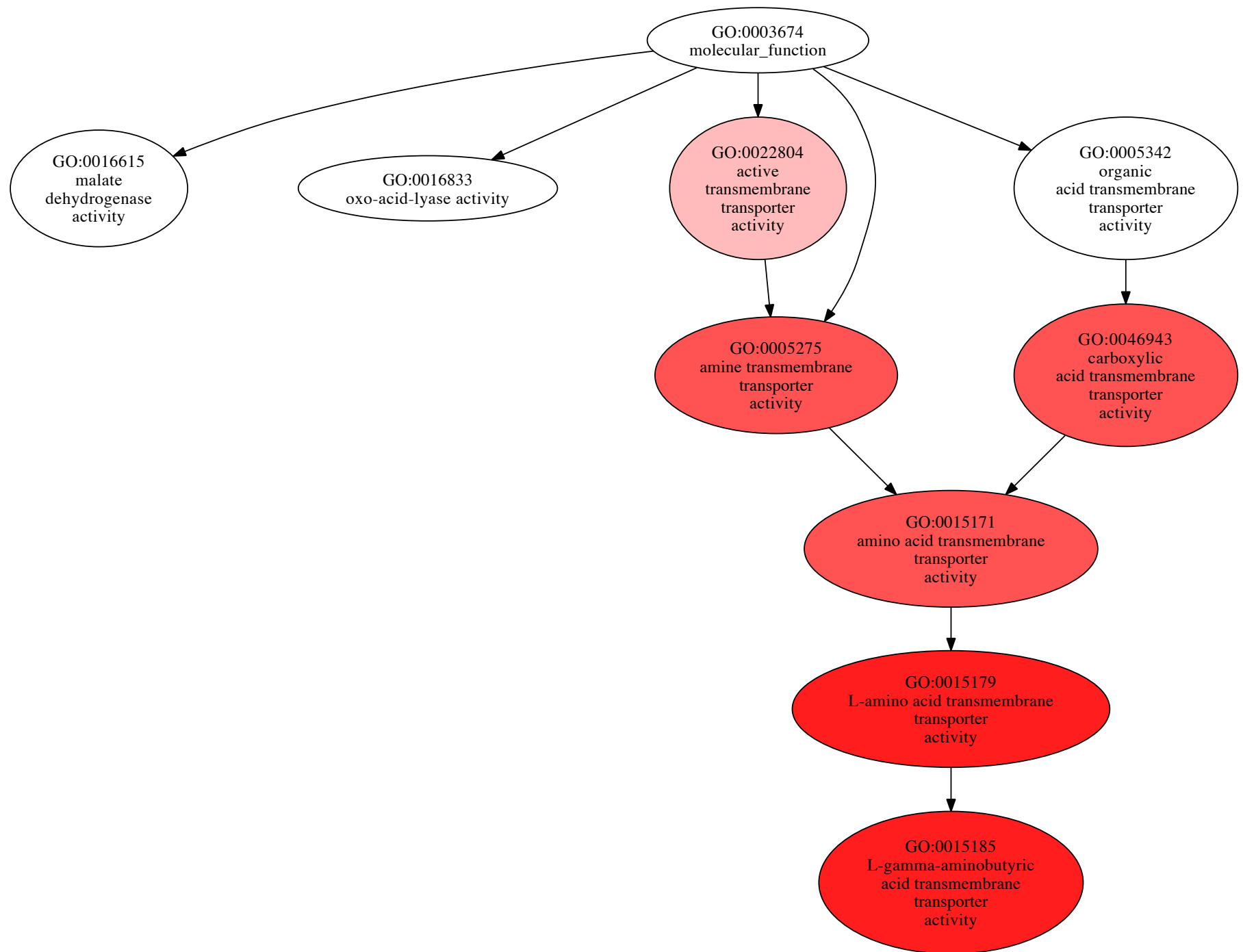

Supplement: Table S3 — ZIP archive file containing FetGOat enrichment results for the up- and down-regulated gene sets of all six comparisons. (ZIP) [file pone.0068946.s003.zip › FetGOat/06.DOWN/plots/06.down.txt_MFover.pdf]

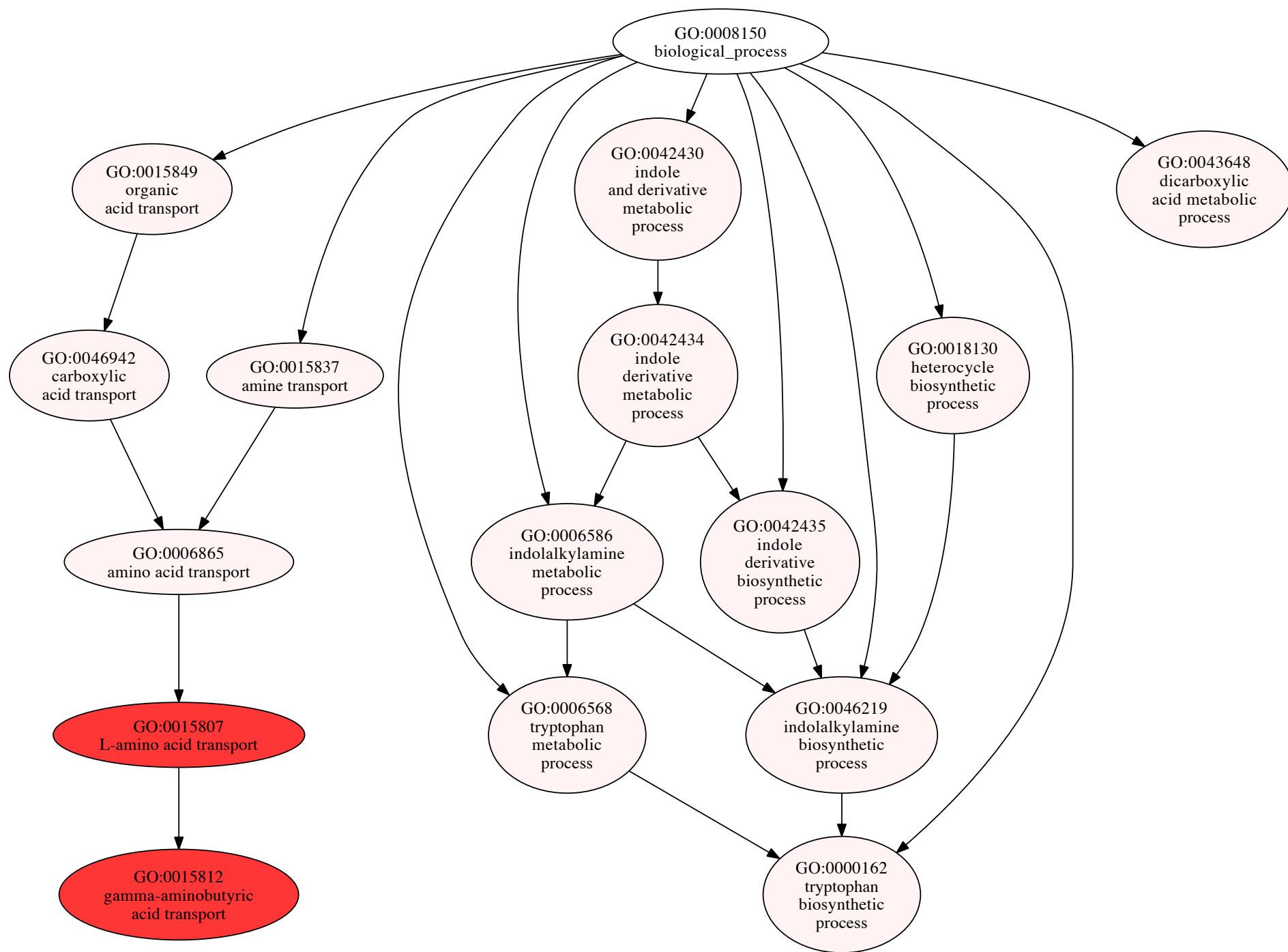

Supplement: Table S3 — ZIP archive file containing FetGOat enrichment results for the up- and down-regulated gene sets of all six comparisons. (ZIP) [file pone.0068946.s003.zip › FetGOat/06.DOWN/plots/06.down.txt_BPover.pdf]

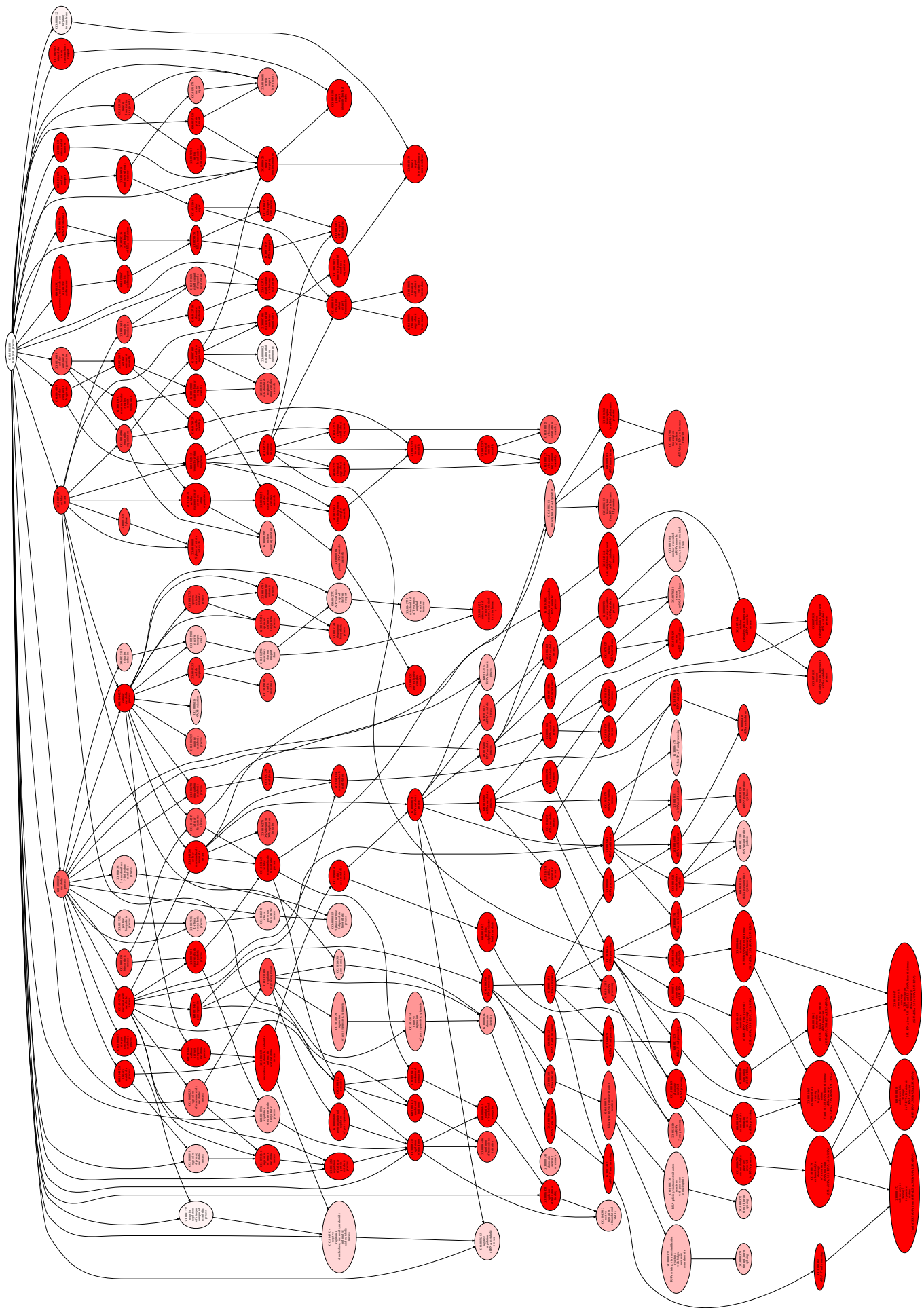

Supplement: Table S3 — ZIP archive file containing FetGOat enrichment results for the up- and down-regulated gene sets of all six comparisons. (ZIP) [file pone.0068946.s003.zip › FetGOat/05.DOWN/plots/05.down.txt_BPover.pdf]

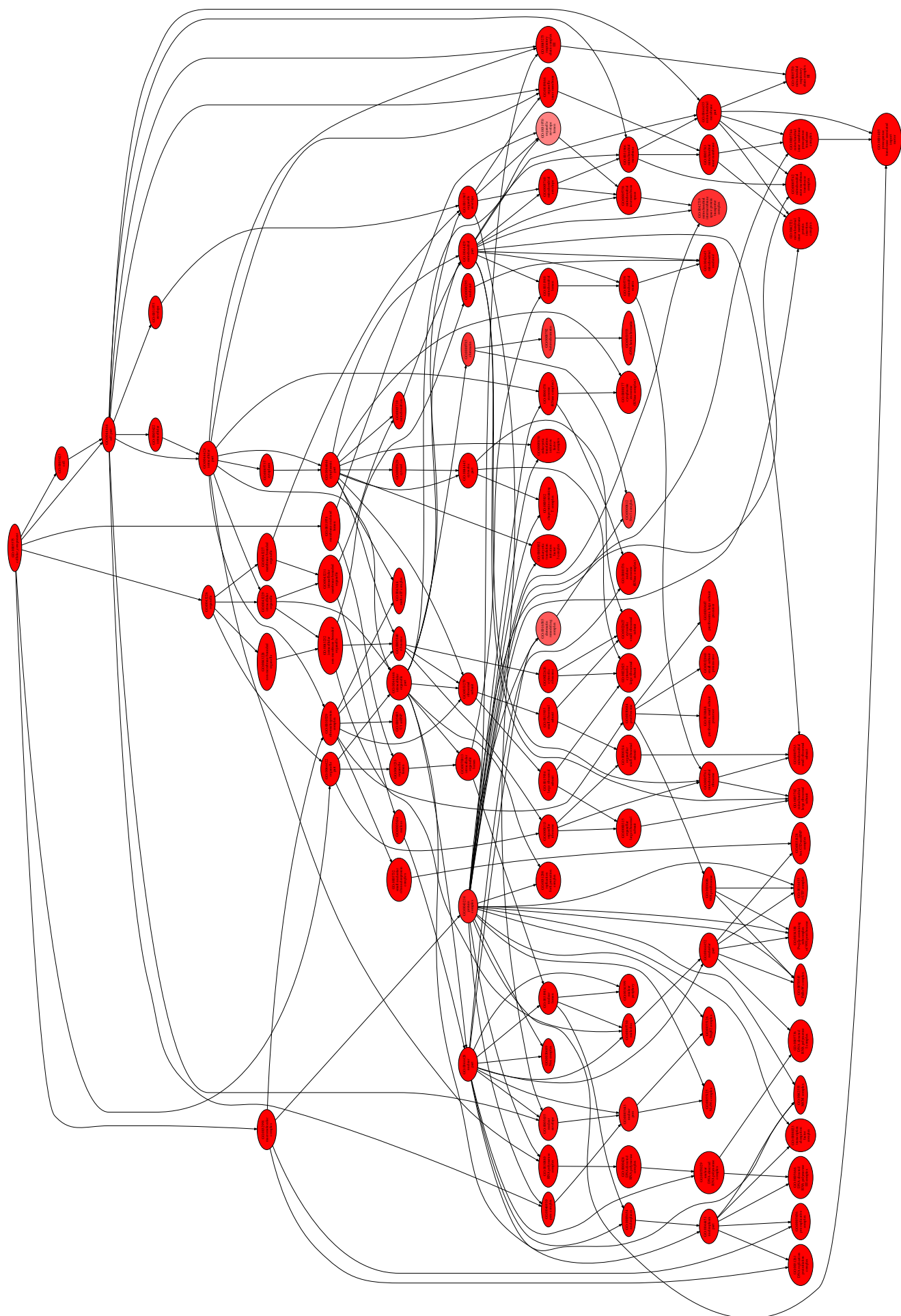

Supplement: Table S3 — ZIP archive file containing FetGOat enrichment results for the up- and down-regulated gene sets of all six comparisons. (ZIP) [file pone.0068946.s003.zip › FetGOat/05.DOWN/plots/05.down.txt_CCover.pdf]

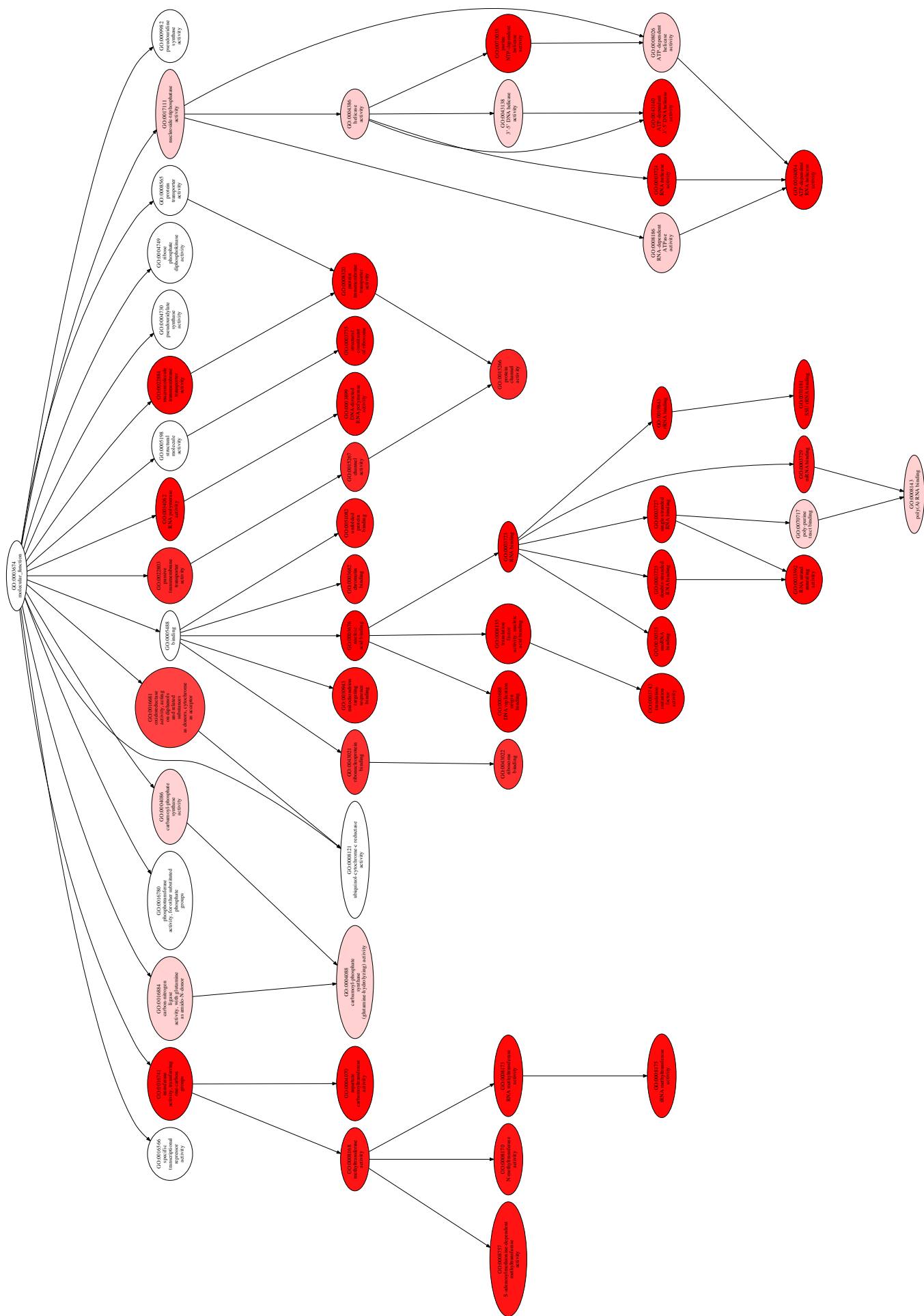

Supplement: Table S3 — ZIP archive file containing FetGOat enrichment results for the up- and down-regulated gene sets of all six comparisons. (ZIP) [file pone.0068946.s003.zip › FetGOat/05.DOWN/plots/05.down.txt_MFover.pdf]

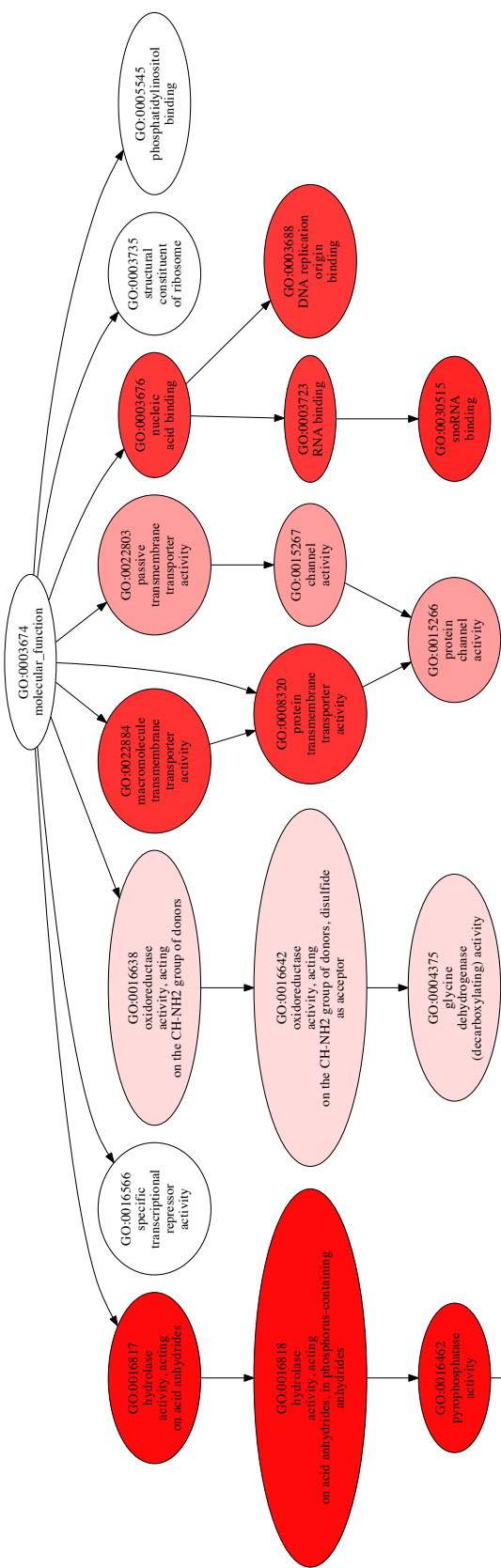

Supplement: Table S3 — ZIP archive file containing FetGOat enrichment results for the up- and down-regulated gene sets of all six comparisons. (ZIP) [file pone.0068946.s003.zip › FetGOat/02.UP/plots/02.up.txt_MFover.pdf]

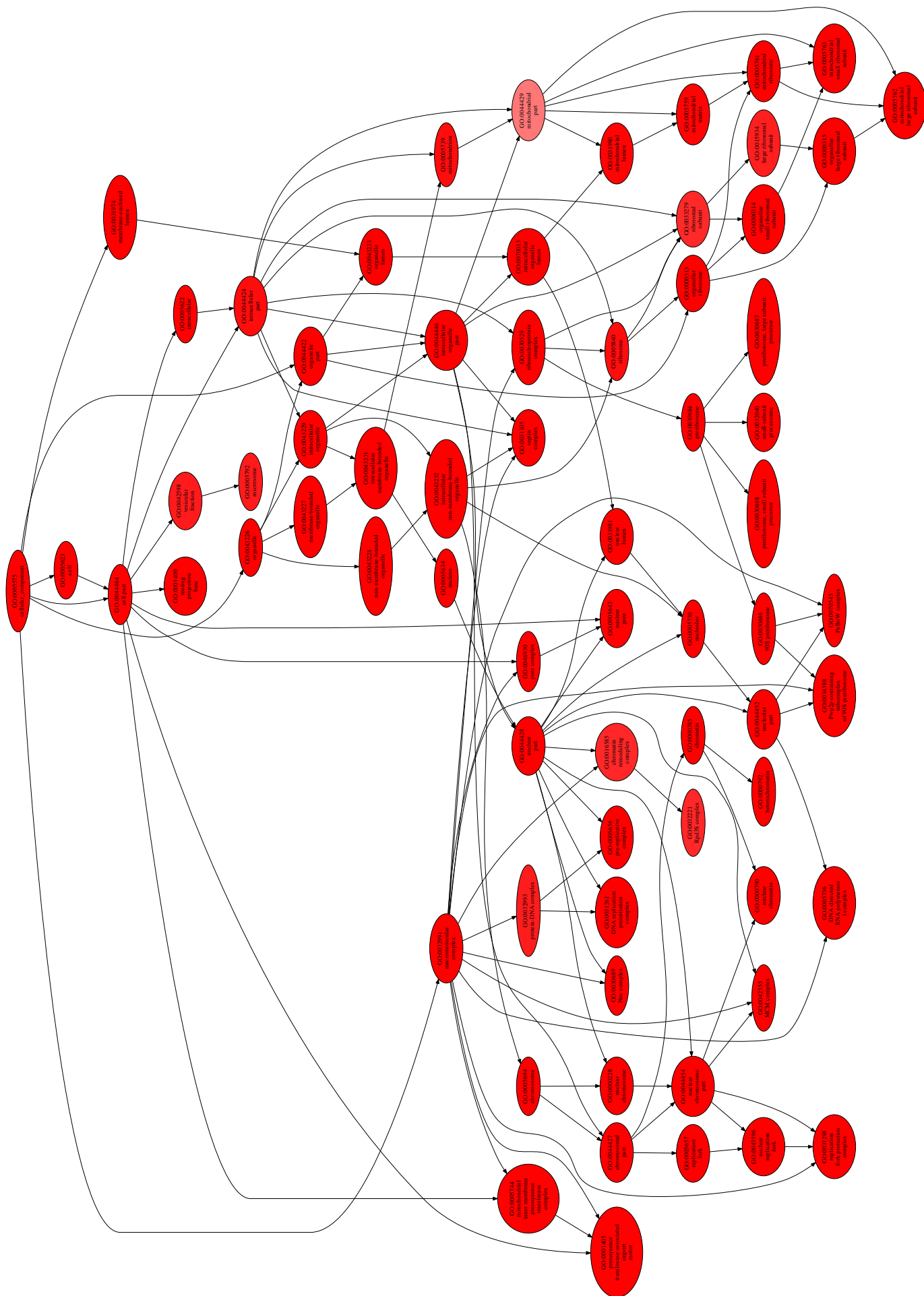

Supplement: Table S3 — ZIP archive file containing FetGOat enrichment results for the up- and down-regulated gene sets of all six comparisons. (ZIP) [file pone.0068946.s003.zip › FetGOat/02.UP/plots/02.up.txt_CCover.pdf]

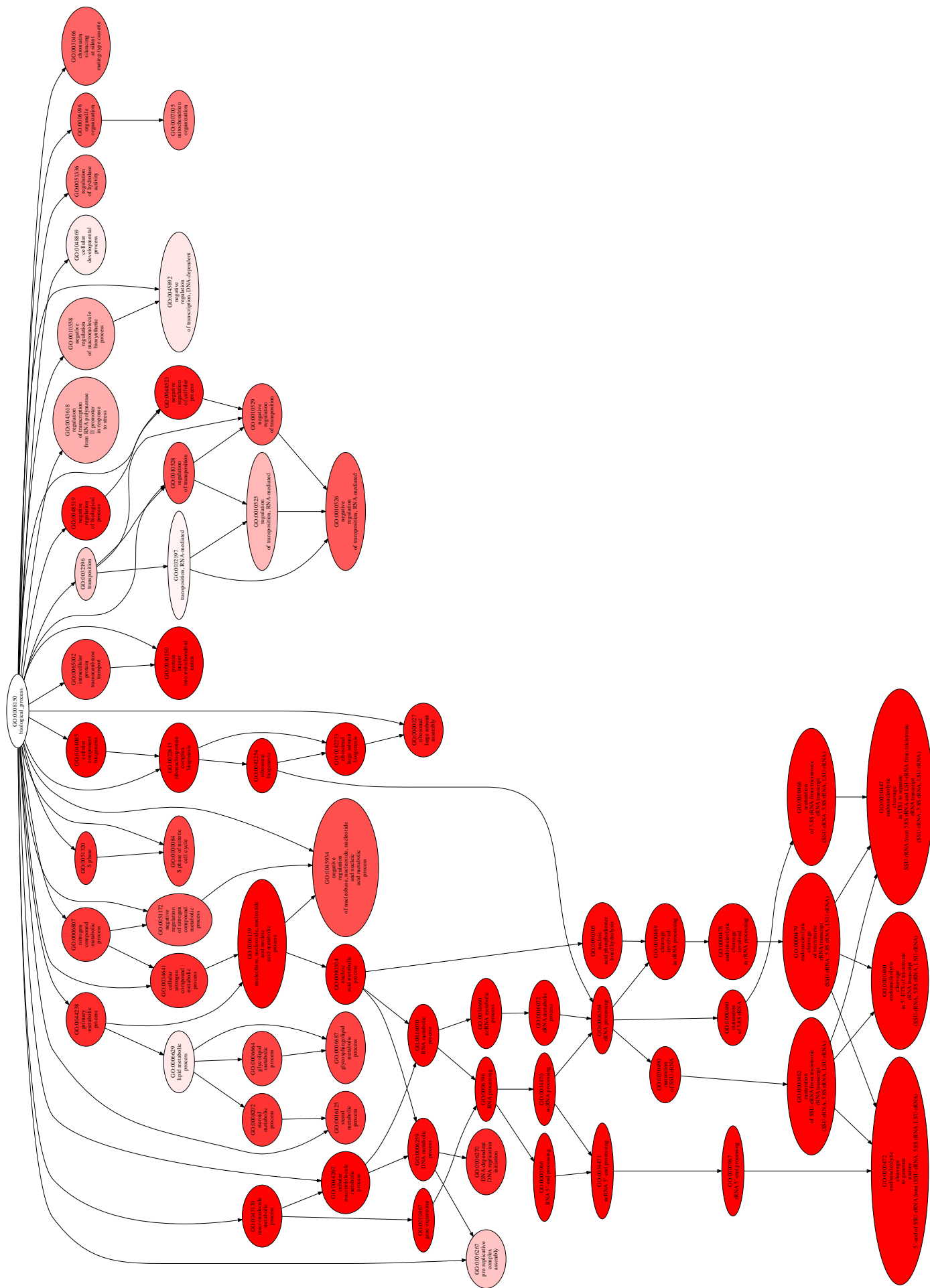

Supplement: Table S3 — ZIP archive file containing FetGOat enrichment results for the up- and down-regulated gene sets of all six comparisons. (ZIP) [file pone.0068946.s003.zip › FetGOat/02.UP/plots/02.up.txt_BPover.pdf]

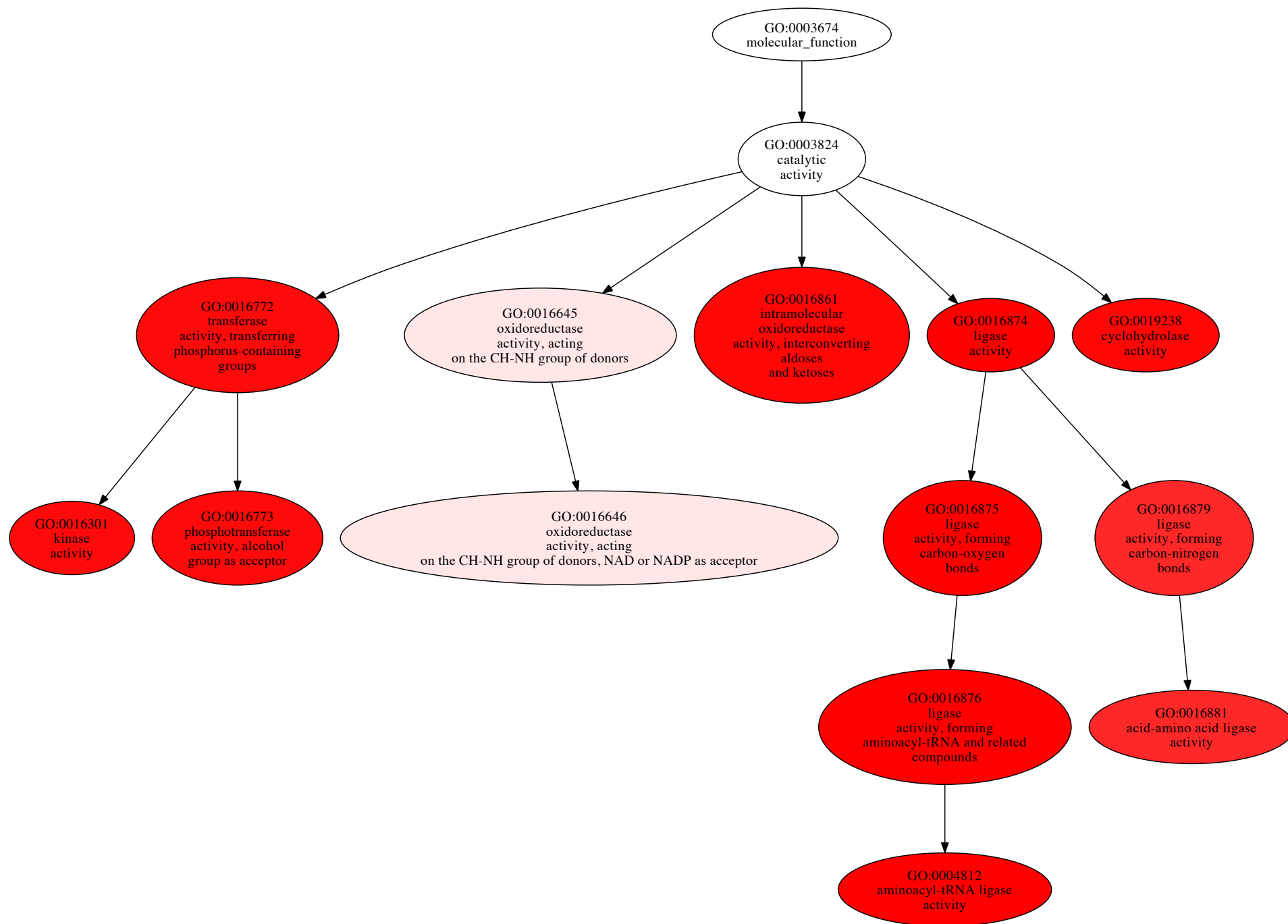

Supplement: Table S3 — ZIP archive file containing FetGOat enrichment results for the up- and down-regulated gene sets of all six comparisons. (ZIP) [file pone.0068946.s003.zip › FetGOat/01.UP/plots/01.up.txt_MFover.pdf]

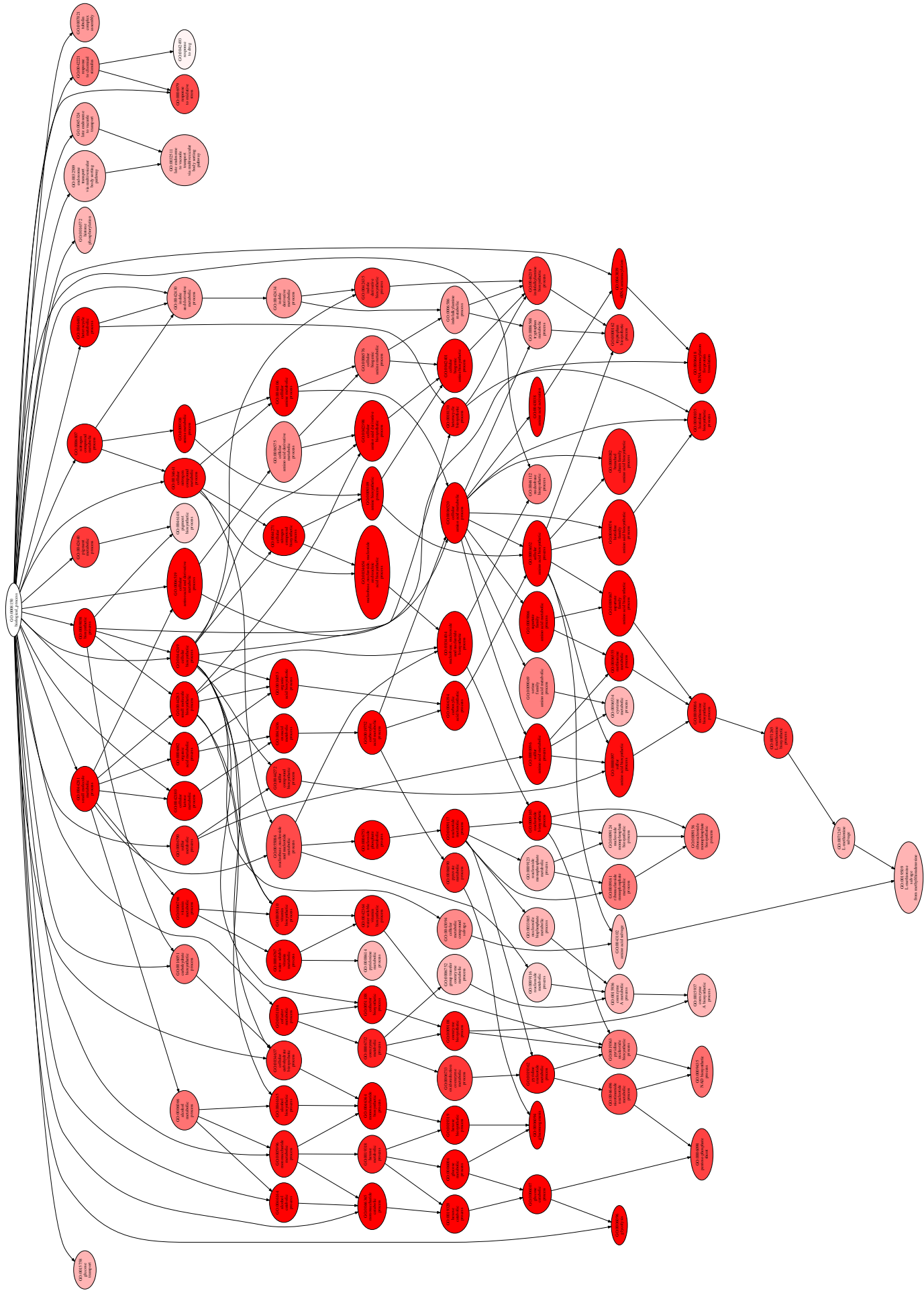

Supplement: Table S3 — ZIP archive file containing FetGOat enrichment results for the up- and down-regulated gene sets of all six comparisons. (ZIP) [file pone.0068946.s003.zip › FetGOat/01.UP/plots/01.up.txt_BPover.pdf]

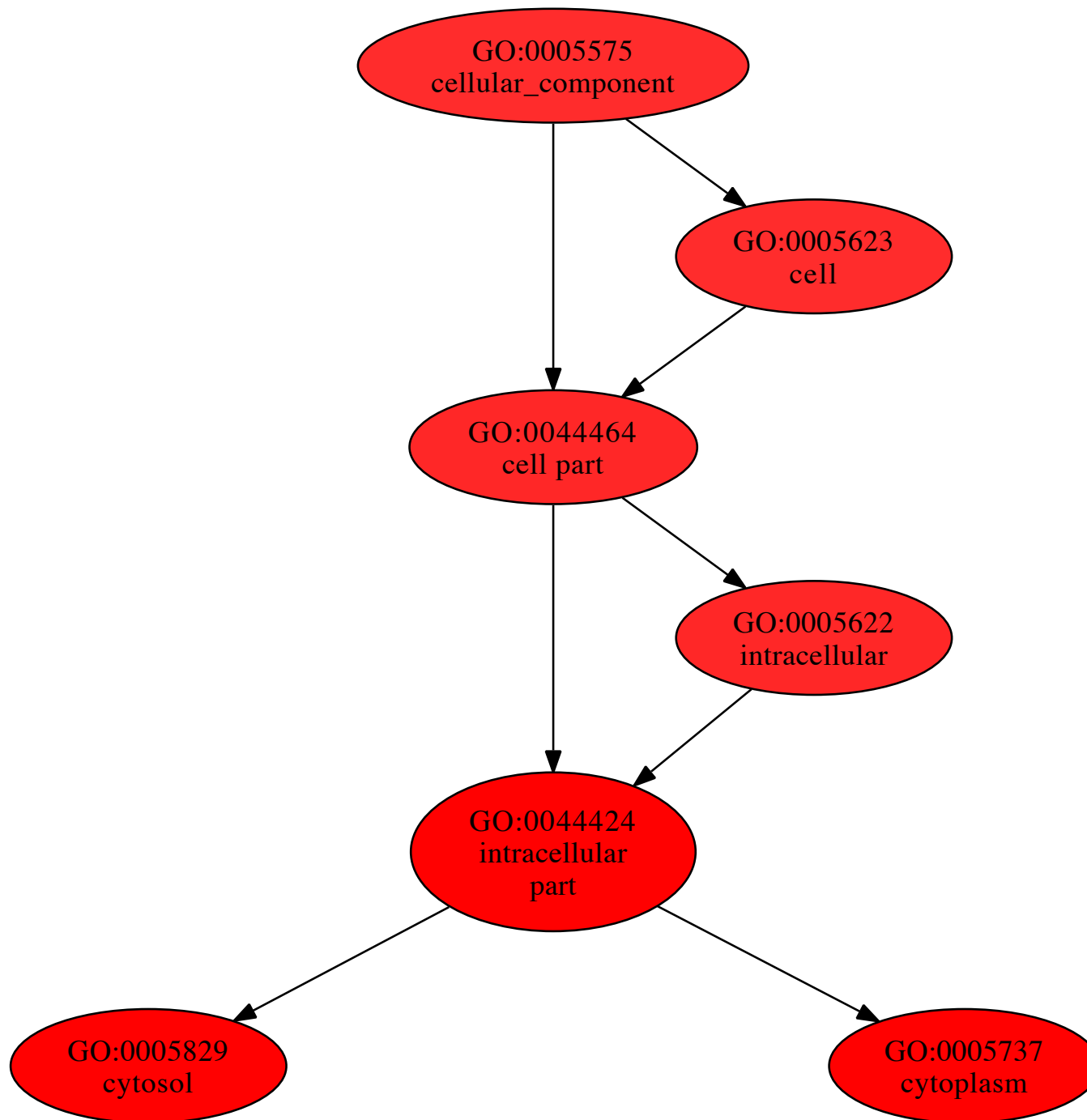

Supplement: Table S3 — ZIP archive file containing FetGOat enrichment results for the up- and down-regulated gene sets of all six comparisons. (ZIP) [file pone.0068946.s003.zip › FetGOat/01.UP/plots/01.up.txt_CCover.pdf]

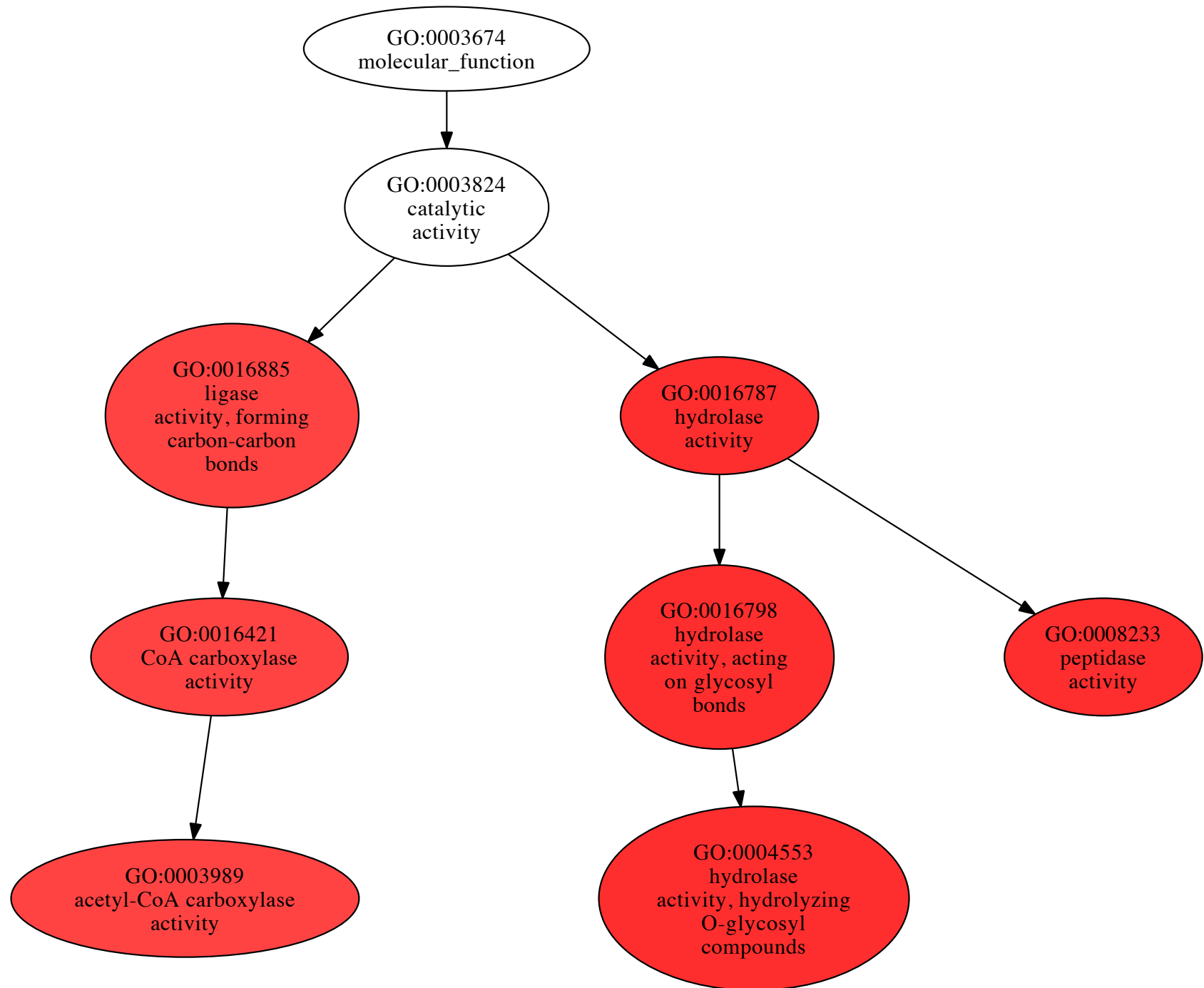

Supplement: Table S3 — ZIP archive file containing FetGOat enrichment results for the up- and down-regulated gene sets of all six comparisons. (ZIP) [file pone.0068946.s003.zip › FetGOat/03.UP/plots/03.up.txt_MFover.pdf]

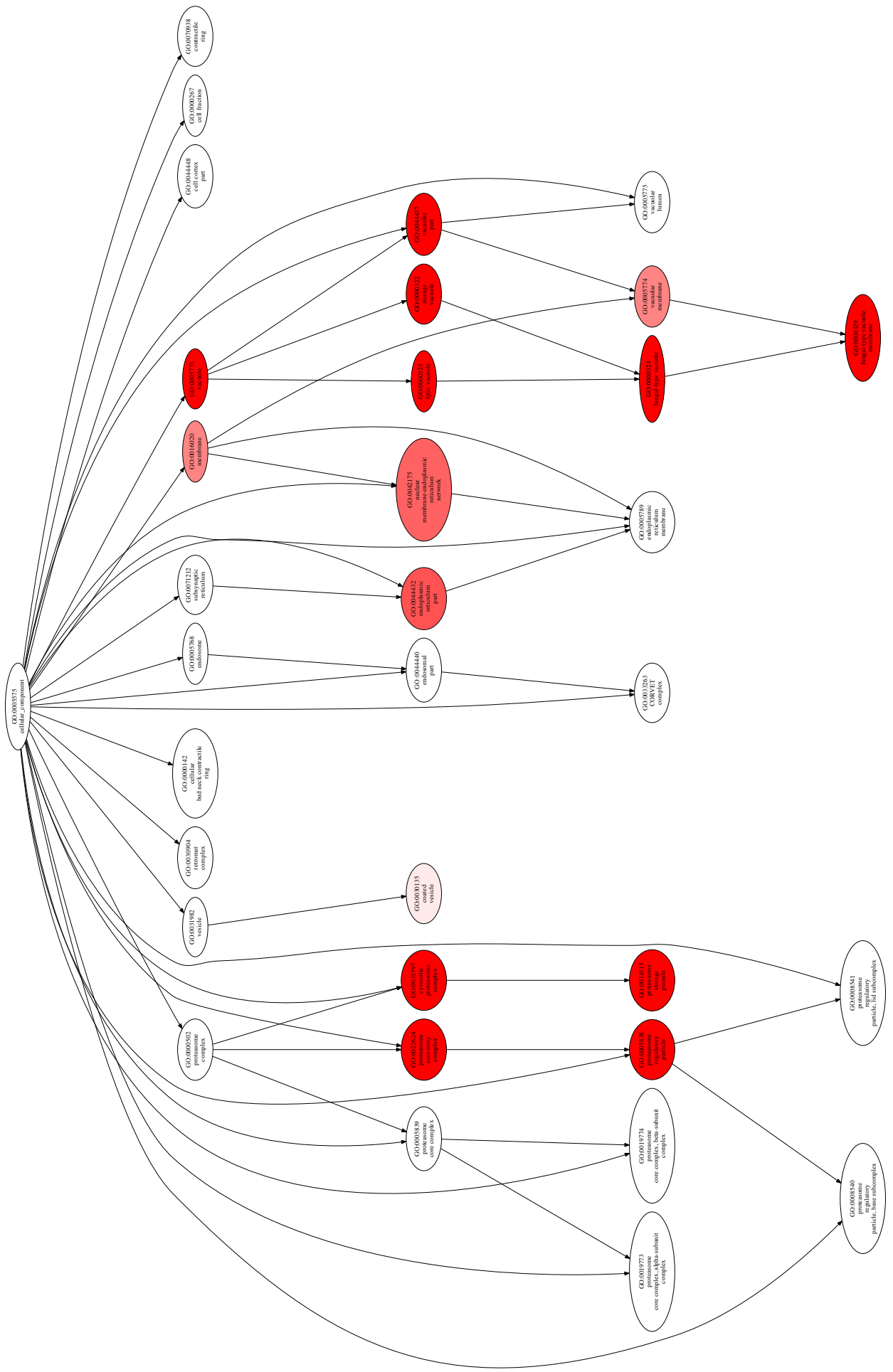

Supplement: Table S3 — ZIP archive file containing FetGOat enrichment results for the up- and down-regulated gene sets of all six comparisons. (ZIP) [file pone.0068946.s003.zip › FetGOat/03.UP/plots/03.up.txt_CCover.pdf]

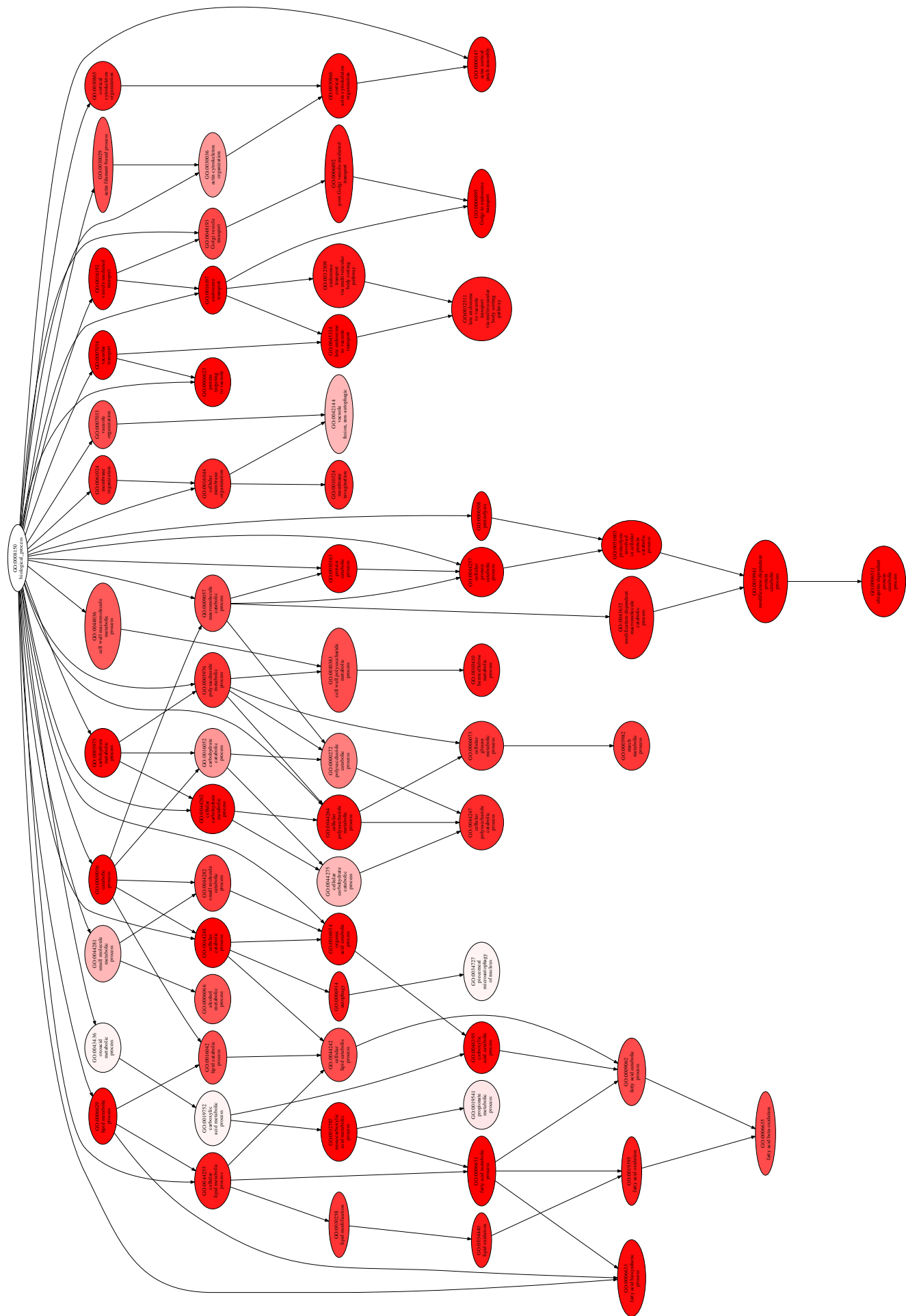

Supplement: Table S3 — ZIP archive file containing FetGOat enrichment results for the up- and down-regulated gene sets of all six comparisons. (ZIP) [file pone.0068946.s003.zip › FetGOat/03.UP/plots/03.up.txt_BPover.pdf]

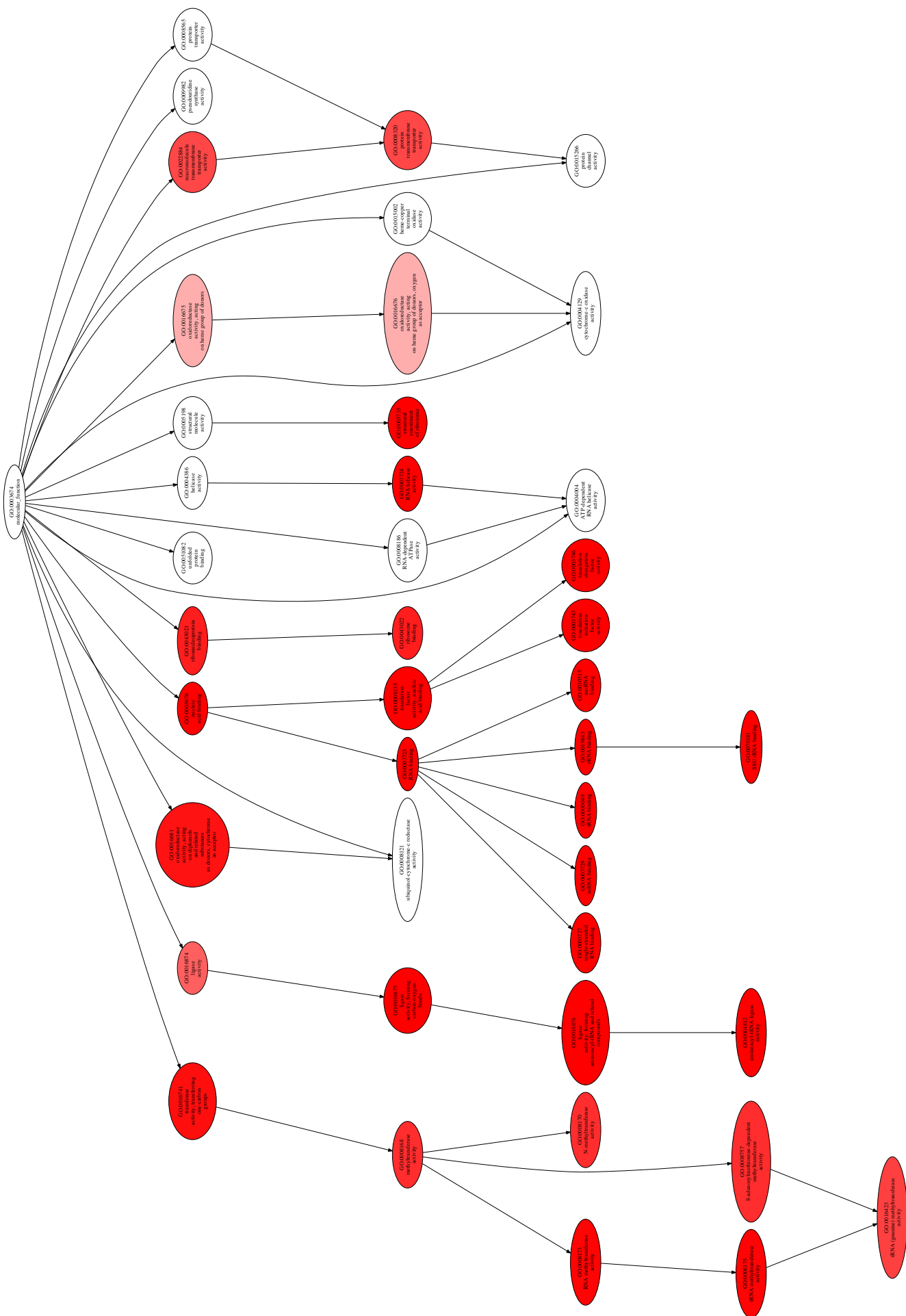

Supplement: Table S3 — ZIP archive file containing FetGOat enrichment results for the up- and down-regulated gene sets of all six comparisons. (ZIP) [file pone.0068946.s003.zip › FetGOat/02.DOWN/plots/02.down.txt_MFover.pdf]

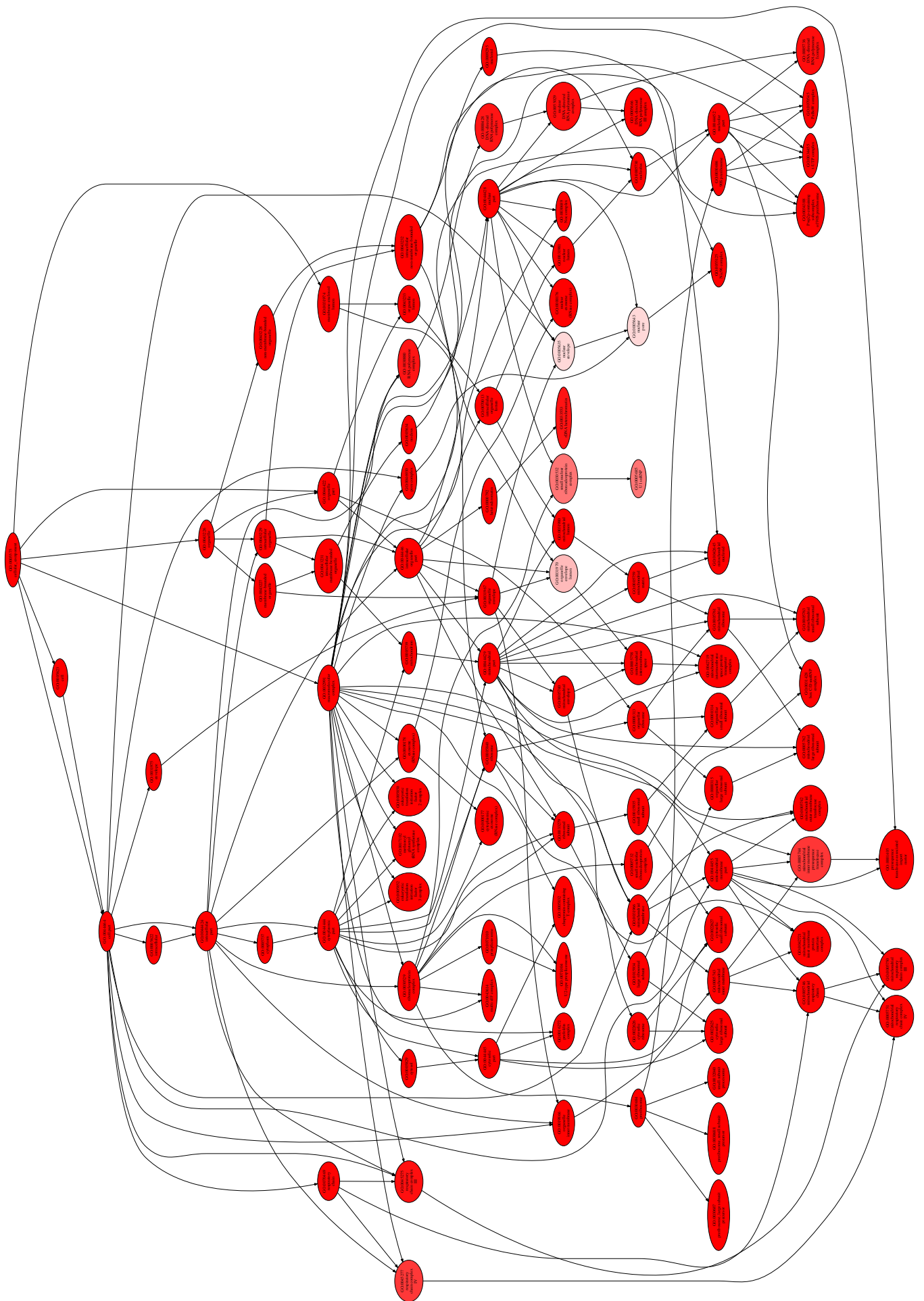

Supplement: Table S3 — ZIP archive file containing FetGOat enrichment results for the up- and down-regulated gene sets of all six comparisons. (ZIP) [file pone.0068946.s003.zip › FetGOat/02.DOWN/plots/02.down.txt_CCover.pdf]

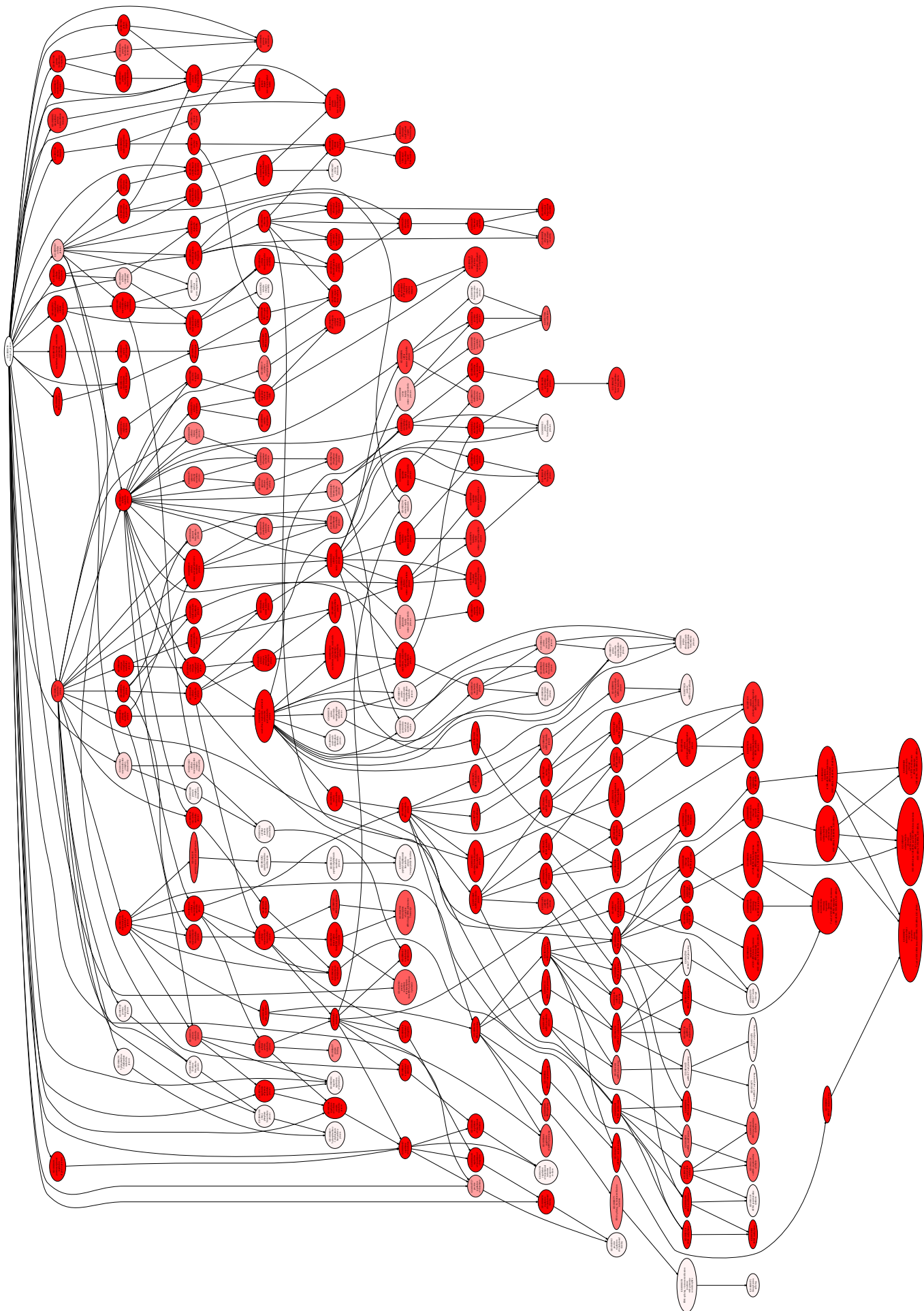

Supplement: Table S3 — ZIP archive file containing FetGOat enrichment results for the up- and down-regulated gene sets of all six comparisons. (ZIP) [file pone.0068946.s003.zip › FetGOat/02.DOWN/plots/02.down.txt_BPover.pdf]
