# Supplementary material for: The Transcriptomic Signature of RacA Activation and Inactivation Provides New Insights into the Morphogenetic Network of Aspergillus niger
Source: PLoS One. 2013 Jul 24;8(7):e68946. doi: 10.1371/journal.pone.0068946 (PMC3722221; doi:10.1371/journal.pone.0068946)
Supplement: Table S5 — Primers used in this study. (DOCX) [file pone.0068946.s005.docx]

**Table S5**. Primers used in this study. Restriction enzymes added are underlined and homologous sequences for fusion PCR are italic.

| Target | Name | Forward/reverse primers (5' 🡪 3') |
| --- | --- | --- |
| T_trpC_ | TrpC-sal for | ACGCGTCGACTAGTGATTTAATAGCTCCATGTC |
|  | TrpC-sal rev | ACGCGTCGACTGGGTGTTACGGAGCATTCACTAGGC |
| AbpA-CFP | AbpA-P1-for | CGATAATGACGTTCCGCAGC |
|  | AbpA-P2-rev | *GGCACCGGCGCCAGCACCAGCGCCGGCACC*TTTCGCAAGTTGCACATAGT |
|  | P3 | *GGTGCCGGCGCTGGTGCTGGCGCCGGTGCC*ATGGTGAGCA AGGGCGAG |
|  | P4 | TGGGTGTTAC GGAGCATTCA CTAGGCAACC |
|  | AbpA-P5-for | *GGTTGCCTAGTGAATGCTCCGTAACACCCA*GCAGTGCACTCCTTGCCTGC |
|  | AbpA-P6-rev | GGATGATTTG CCGGACGAC |
| SlaB-YFP | SlaB-P1-for | TTAACGCGGTTCGCCAAC |
|  | SlaB-P2-rev | *GGCACCGGCGCCAGCACCAGCGCCGGCACC*GTCCTCCTGGTAAGAAATC |
|  | SlaB-P5-for | *GGTTGCCTAGTGAATGCTCCGTAACACCCA*GTGGCTGCCACCATGTTTTT G |
|  | SlaB-P6-rev | ATCTGCACAGGCTGGGACA |
|  |  |  |
