# Supplementary material for: The Transcriptomic Signature of RacA Activation and Inactivation Provides New Insights into the Morphogenetic Network of Aspergillus niger
Source: PLoS One. 2013 Jul 24;8(7):e68946. doi: 10.1371/journal.pone.0068946 (PMC3722221; doi:10.1371/journal.pone.0068946)
Supplement: Table S3 — ZIP archive file containing FetGOat enrichment results for the up- and down-regulated gene sets of all six comparisons. (ZIP) [file 01.down.txt_MFover.pdf › FetGOat/01.DOWN/plots/01.down.txt_MFover.pdf]

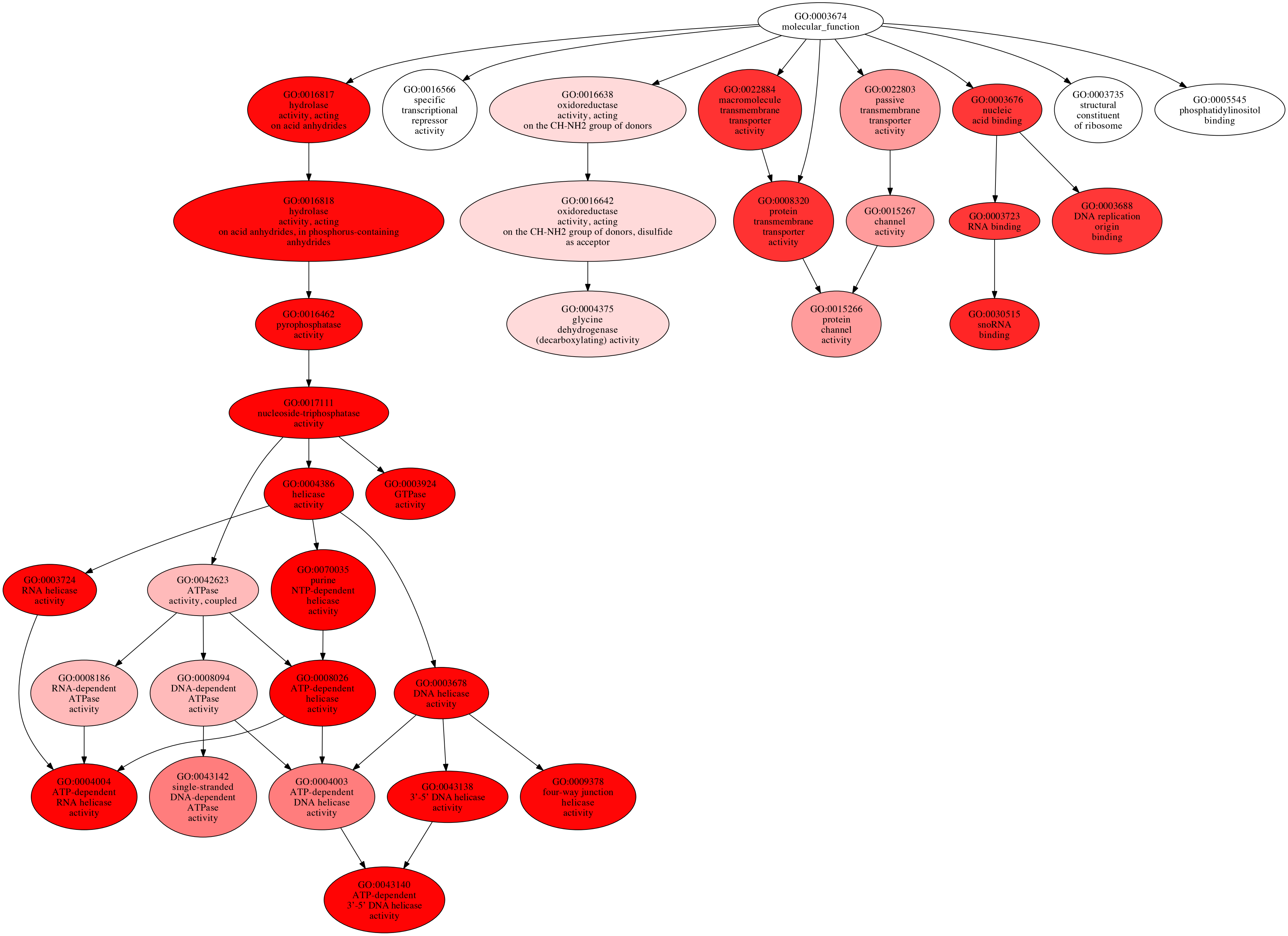
GO:0003674 molecular_function
GO:0016817 hydrolase activity, acting on acid anhydrides
GO:0016566 specific transcriptional repressor activity
GO:0016638 oxidoreductase activity, acting on the CH-NH2 group of donors
GO:0022884 macromolecule transmembrane transporter activity
GO:0022803 passive transmembrane transporter activity
GO:0003676 nucleic acid binding
GO:0003735 structural constituent of ribosome
GO:0005545 phosphatidylinositol binding
GO:0016818 hydrolase activity, acting on acid anhydrides, in phosphorus-containing anhydrides
GO:0016642 oxidoreductase activity, acting on the CH-NH2 group of donors, disulfide as acceptor
GO:0008320 protein transmembrane transporter activity
GO:0015267 channel activity
GO:0003723 RNA binding
GO:0003688 DNA replication origin binding
GO:0016462 pyrophosphatase activity
GO:0004375 glycine dehydrogenase (decarboxylating) activity
GO:0015266 protein channel activity
GO:0030515 snoRNA binding
GO:0017111 nucleoside-triphosphatase activity
GO:0004386 helicase activity
GO:0003924 GTPase activity
GO:0003724 RNA helicase activity
GO:0042623 ATPase activity, coupled
GO:0070035 purine NTP-dependent helicase activity
GO:0008186 RNA-dependent ATPase activity
GO:0008094 DNA-dependent ATPase activity
GO:0008026 ATP-dependent helicase activity
GO:0003678 DNA helicase activity
GO:0004004 ATP-dependent RNA helicase activity
GO:0043142 single-stranded DNA-dependent ATPase activity
GO:0004003 ATP-dependent DNA helicase activity
GO:0043138 3'-5' DNA helicase activity
GO:0009378 four-way junction helicase activity
GO:0043140 ATP-dependent 3'-5' DNA helicase activity
